# Mutually reinforcing and transpiration-dependent propagation of H_2_O_2_ and variation potential in plants revealed by fiber organic electrochemical transistors

**DOI:** 10.1016/j.xinn.2025.100800

**Published:** 2025-01-06

**Authors:** Hanqi Wen, Lingxuan Kong, Xinlu Zhu, Yansong Miao, Xing Sheng, Xiaodong Chen, Yuxin Liu, Peng Chen

**Affiliations:** 1 School of Chemistry, Chemical Engineering and Biotechnology, Nanyang Technological University, Singapore 637457, Singapore; 2 Institute of Flexible Electronics Technology of THU, Jiaxing 314000, China; 3 School of Biological Sciences, Nanyang Technological University, Singapore 637551, Singapore; 4 Institute for Digital Molecular Analytics and Science (IDMxS), Nanyang Technological University, Singapore 636921, Singapore; 5 Department of Electronic Engineering, Beijing National Research Center for Information Science and Technology, Laboratory of Flexible Electronics Technology, Tsinghua University, Beijing 100084, China; 6 Innovative Center for Flexible Devices (iFLEX), Max Planck-NTU Joint Laboratory for Artificial Senses, School of Materials Science and Engineering (MSE), Nanyang Technological University, Singapore 639798, Singapore; 7 Department of Biomedical Engineering (BME), National University of Singapore, Singapore 117583, Singapore; 8 The N.1 Institute for Health, National University of Singapore, Singapore 117456, Singapore; 9 Institute for Health Innovation and Technology (iHealthtech), National University of Singapore, Singapore 117599, Singapore

**Keywords:** organic electrochemical transistors, variation potential, H_2_O_2_ wave, transpiration, plant sensors

## Abstract

Plants use hydrogen peroxide (H_2_O_2_) and variation potential (VP) waves as well as chemical transport by transpiration-driven xylem flow to facilitate cell signaling, cell-to-cell communication, and adaptation to environmental stresses. The underlying mechanisms and complex interplay among H_2_O_2_, VP, and transpiration are not clearly understood because of the lack of bioengineering tools for continuous *in planta* monitoring of the dynamic biological processes. Here, we tackle the challenge by developing microfiber-shaped organic electrochemical transistors (fOECTs) that can be threaded into the plants. The sensorized microfiber revealed that both H_2_O_2_ and VP waves propagate faster toward the leaves than toward the roots because of the directional long-distance transport of H_2_O_2_ in the xylem. In addition, the revealed interplays among VP, H_2_O_2_, and xylem flow strongly suggest a transpiration- and intensity-dependent H_2_O_2_-VP mutual-reinforcing propagation mechanism. The microfiber electronics offer a versatile platform for the *in situ* study of dynamic physiological processes in plants with high temporospatial resolution.

## Public summary

• Plants use hydrogen peroxide (H₂O₂), variation potential (VP), and xylem flow for cell signaling and adaptation.
• Microfiber-shaped organic electrochemical transistors (fOECTs) reveal interplays among VP, H₂O₂, and xylem flow.
• A transpiration- and intensity-dependent H₂O₂-VP mutual-reinforcing propagation mechanism is proposed.
• Microfiber electronics enable the *in situ* study of plant physiological processes with high temporospatial resolution.

## Graphical abstract

**Figure:**
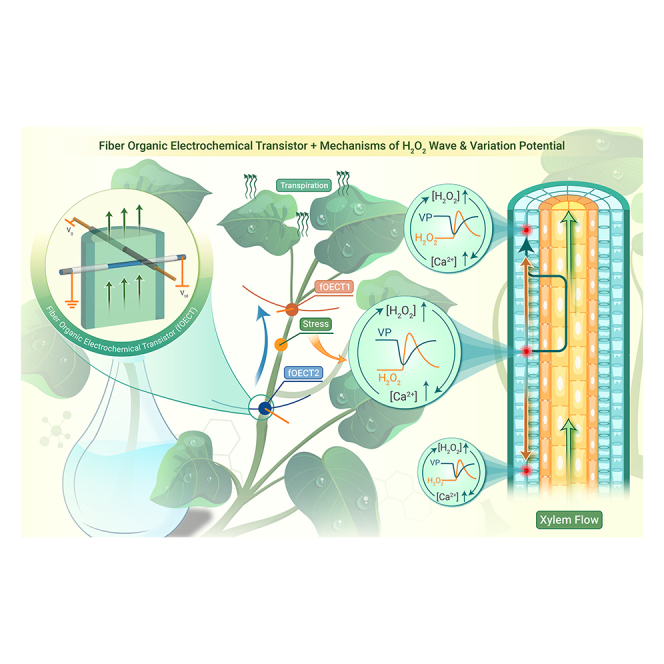

## Introduction

Initially only considered as the inevitable toxic product of metabolic and enzymatic activities, H_2_O_2_, the major reactive oxygen species (ROS) in plants, has been increasingly recognized as the crucial signaling molecule playing roles in various plant physiological processes and responses to biotic or abiotic stresses (e.g., wounding), thanks to its long lifespan.^1^^,^^2^ Some studies^3^^,^^4^^,^^5^ have suggested that the propagation of the H_2_O_2_ signal is coupled with Ca^2+^ signaling and propagating variation potential (VP) to produce coordinated responses to stresses through some not-well-understood mechanisms. We further reason that transpiration-driven xylem flow may play a key role in long-distance H_2_O_2_ signaling for cell-to-cell communication. The mechanisms of these dynamic processes and the coupling between the H_2_O_2_ wave, VP, and transpiration are still under debate, largely due to the lack of probing tools with high sensitivity, sufficient temporal and spatial resolution, minimal invasiveness, and compatibility with plant tissues.

Currently, H_2_O_2_ is monitored using fluorescence probes,^6^^,^^7^^,^^8^ but they are not able to report the H_2_O_2_ dynamics because their interaction with H_2_O_2_ is irreversible or slow, and long-term monitoring is not possible due to photobleaching.^9^ Additionally, it is difficult to calibrate the exact H_2_O_2_ concentration because the fluorescence signal can be significantly interfered with by the physiological conditions in plant tissues, such as pH.^10^ In the recent seminal study by Strano et al., the wound-induced H_2_O_2_ wave on the leaves of various plant species was spatiotemporally monitored for the first time using H_2_O_2_-selective fluorescent single-walled carbon nanotubes.^11^ But still, it shares some problems with other fluorescence imaging methods. First, the measurement is compromised by the undefined diffusion and distribution of the fluorescence probe. Secondly, image acquisition is too slow to resolve the fast signaling events. Thirdly, deep tissue monitoring is prevented by the limited light penetration depth.

The xylem flow rate is often determined by the heat dissipation method (HDM) or isotope tracing technique.^12^ The former, which involves the insertion of large probes and heating, is highly invasive, inherently only applicable to large woody plants, and of low temporal resolution. The latter requires a special experimental facility and condition. Existing solid-state electrodes for VP recording exhibit high and unstable impedance at the electrode-fluid interface and substantial mechanical mismatch with soft plant tissue, which cause adverse biological reactions, high noise, and motion artifacts.^13^

To address the abovementioned research gaps and technical challenges, we report microfiber-shaped organic electrochemical transistors (fOECTs). The conductive polymer-based devices allow long-term and continuous *in planta* recording with a stable bioelectronic interface.^14^ Leveraging its dual electronic-ionic conductivity and excellent biological mechanical compatibility,^15^^,^^16^^,^^17^ microfiber electronics was used to record the transpiration-driven xylem flow, H_2_O_2_ wave, and VP with sub-second temporal resolution. The generalizable microfiber electronics platform enables the observation of temporospatial dynamics and interplay between these processes and further leads to a proposed mechanism of transpiration- and intensity-dependent H_2_O_2_-VP mutual-reinforcing propagation.

## Materials and methods

### Preparation and characterization of M^+^-fOECT and H_2_O_2_-fOECT

Poly(3,4-ethylenedioxythiophene) polystyrene sulfonate (PEDOT:PSS) solution (PH1000), dimethyl sulfoxide (DMSO), ethylene glycol (EG), 4-dodecylbenzenesulfonic acid (DBSA), 5% Nafion solution, chloroplatinic acid hexahydrate (H_2_PtCl_6_·6H_2_O), diphenyleneiodonium chloride (DPI), GdCl_3_, and 3% w/w H_2_O_2_ solution were purchased from Sigma-Aldrich. 0.2% w/w DBSA, 5% w/w EG, and 5% w/w DMSO were added into the PEDOT:PSS solution with stirring, followed by sonication for 30 min and filtering by a 0.45 μm nylon syringe filter to remove aggregates. Silk fiber was cleaned by sonication in deionized (DI) water, ethanol, and acetone successively and then dried in a 70°C oven. The fiber was then dip coated in the prepared PEDOT:PSS solution and drawn by a tensile meter at a speed of 2 cm s^−1^ to maintain a constant shear rate of 50 s^−1^, thereby forming a uniform conductive layer on the surface. The coated fiber was dried in an oven at 75°C for 30 min and then further heated at 125°C for 30 min. Silver paste (Dycotech) was applied to both ends of PEDOT:PSS fibers except for the middle (5 mm), which acted as the sensing region. Except for the two contact ends, a layer of Ecoflex (BASF) was coated on the silver paste as an insulating layer.

Two of the obtained conductive microfibers constitute an M^+^-fOECT. The H_2_O_2_-fOECT consists of a conductive microfiber as the source-drain channel and a Pt nanoparticle (PtNP)-coated Au wire as the gate electrode. An Au wire (0.25 mm) was successively cleaned by DI water, isopropyl alcohol, and acetone. After drying in a 70°C oven, it was further cleaned by 10 cycles of cyclic voltammetry (CV) scanning in 0.5 M H_2_SO_4_ from 0 to 1 V with a scan rate of 100 mV/s. PtNPs were electrochemically deposited on gold wire by CV sweep from 0 to 1.1 V (with a saturated calomel electrode as reference) in an electrolyte containing 0.5 M H_2_SO_4_ and 1 mM H_2_PtCl_6_. After being rinsed by DI water, the gate electrode was immersed in Nafion (5% in ethanal) for 30 min and naturally dried in a fume hood. All fOECT experiments were conducted on a semiconductor analyzer (Keithley 4200 SCS). To obtain the output curve, V_sd_ was swept from 0 to −1 V under various gate voltages (V_g_). For the transfer and transconductance (g_m_) curves, V_g_ was swept from −0.2 to 0.6 V with a step size of 0.05 V and V_sd_ at −0.2 V.

### Plant material, growth condition, and *in situ* measurements

Devil’s ivy (*Epipremnum aureum*) was collected from a campus nursery and grown for 1 week at 21°C and 80% relative humidity under a 14-h-light/10-h-dark photoperiod, 50 μmol s^−1^ m^−2^ light intensity. The plants for experiments have only branches with 2–5 leaves (total surface area of ∼100 cm^2^). To have convenient control of the cultivation and experimental conditions, the plants were kept hydroponically. Microfibers were threaded through the plant stem using a sewing needle. Wounding was inflicted using a needle (diameter of 0.45 mm unless stated otherwise). To induce heat stress, 50°C water was continuously dropped onto the plant stem for 30 s. The temperature of the heated region was determined to be 45°C by an infrared (IR) camera. VP was recorded by a differential amplifier (BrainVision). For the light stimulation experiment, before the plant was irradiated by a solar light simulator (94023A, Newport), it was kept in a dark room for 12 h. Agents were infused into the plant stem through a previously created small hole (inflicted 30 min earlier). The take-off point is when the signal amplitude reaches 5× of the baseline fluctuation. The rise time is defined as the time gap between the take-off point and when the signal reaches 63.2% of the peak. By measuring the distance and the take-off time-point differences of two sensors, the velocity of signal propagation is calculated.

## Results and discussion

### fOECT device designed for continuous *in planta* monitoring

**Figure 1:**
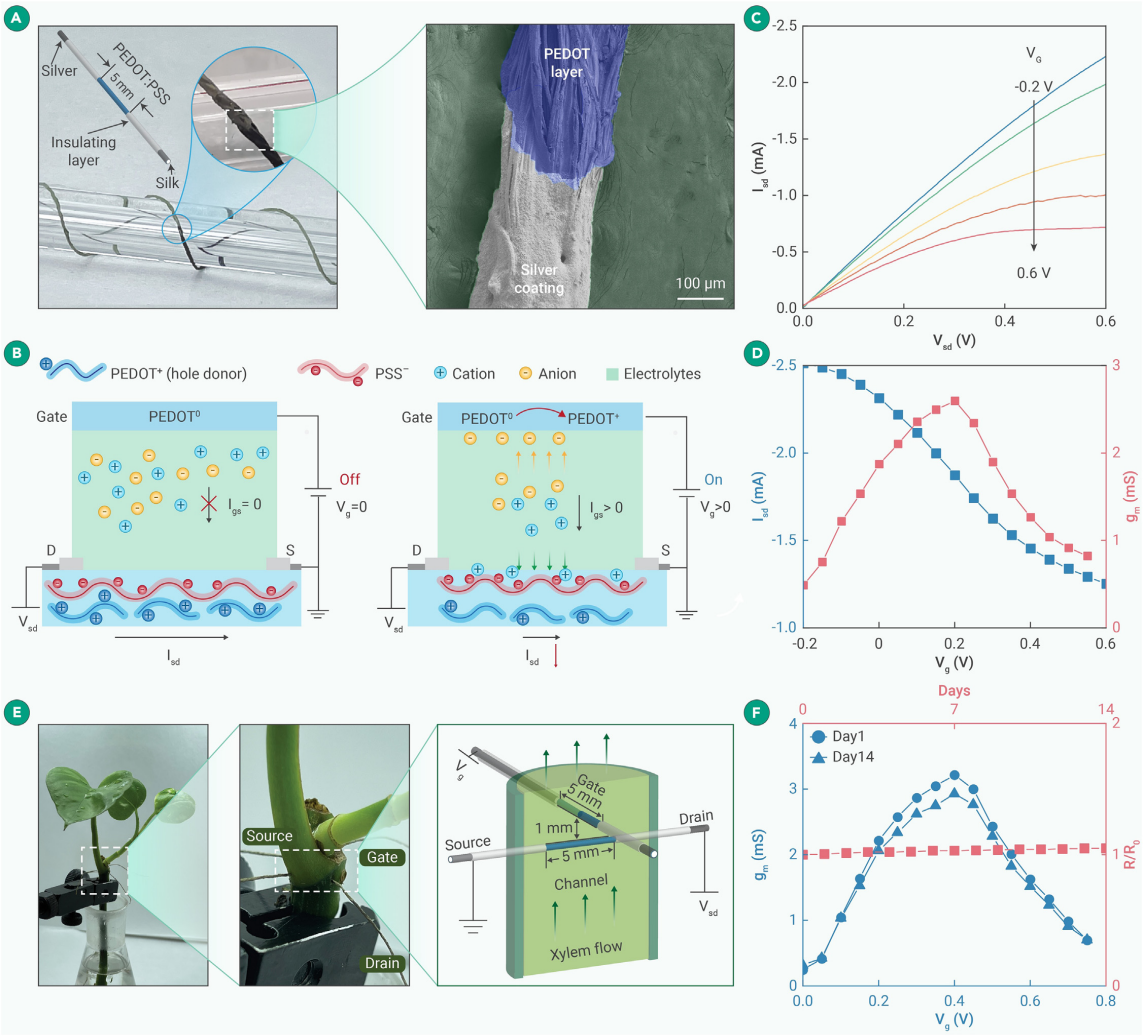
Characterizations of the M^+^-fOECT (A) Schematic, optical (left), and scanning electron microscopy (SEM; right; pseudo-coloring applied to PEDOT:PSS layer) images of a microfiber. (B) Cation-responsive mechanism of M^+^-fOECT. (C) Output curve of M^+^-fOECT in agarose gel containing 75 mM KCl solution. (D) Transfer (black) and transconductance (red) curves of M^+^-fOECT in agarose gel containing 75 mM KCl solution. (E) fOECT threaded in a devil’s ivy. (F) Transconductance curves of M^+^-fOECT (black) immediately and 14 days after implantation and resistance variation (red) over the course.

Conventional OECT devices are fabricated on planar substrates (e.g., silicon).^18^ To minimize tissue invasiveness, one-dimensional (1D) microfiber-shaped OECTs can be developed.^19^^,^^20^^,^^21^ Herein, degummed silk fiber was used as the substrate material due to its small diameter (∼240 μm), hydrophilicity, excellent biocompatibility, low Young’s modulus, good mechanical strength, and high flexibility (Figure 1A).^22^ A thin layer of PEDOT:PSS (∼5 μm) was dip coated on the fiber to form a conducting channel. Assisted by surfactant DBSA, PEDOT:PSS chains electrostatically interact with the hydrophilic silk fiber, allowing uniform coating along the fiber (as evidenced in Figure S1), and polar molecules (DMSO and EG) were used to further improve the conductivity by weakening the electrostatic interaction between PEDOT and PSS, consequently straightening coiled PEDOT chains to give a lower energy barrier for inter-grain charge hopping. After the silver paste was coated on both ends as the source and drain, the fiber was insulated with Ecoflex polymer except for the middle sensing region (Figure 1A).

A cation-responsive fOECT (M^+^-fOECT) was constructed by two PEDOT:PSS microfibers. When the gate fiber is biased by a positive voltage (V_g_), it is oxidized, while in the channel fiber, PEDOT is reduced and thus dedoped (Figure 1B).^23^ Cations in the electrolyte (M^+^) are driven in to neutralize the decoupled polyanion (PSS^−^):

gate: PEDOT^0^ – e^−^ = PEDOT^+^ and

channel: PEDOT^+^PSS^−^ + e^−^ + M^+^ = PEDOT^0^ + PSS^−^M^+^.

Consequently, the decreased hole density on the channel fiber gives a lower source-drain current (I_sd_) in a cation-concentration-dependent manner. Owing to the reversible doping/dedoping process,^24^ the M^+^-fOECT exhibits good cycling stability with <10% current variation over 3,000 cycles (Figure S2), and its small response time constant (0.11 s) ensures the capability to resolve fast dynamic biological processes (Figure S3). Furthermore, the M^+^-fOECT is not responsive to H_2_O_2_ (Figure S4A).

The performance of the M^+^-fOECT was first evaluated in an artificial tissue model, i.e., agarose gel (mimicking plant tissue and interstitial fluid) covered with a water-impermeable parafilm (mimicking plant cuticle). With a small V_sd_ (−0.2 V) being applied, the V_g_ negatively modulates I_sd_ in a depletion mode (Figure 1C). g_m_, which is the partial derivative between I_sd_ and V_g_, characterizes the sensitivity of an OECT. As shown by the transfer and g_m_ curves (Figure 1D), the highest g_m_ (2.6 mS) was achieved at V_g_ = ∼0.2 V, which is thus used for all cation recording experiments. The device-to-device variation in g_m_ is small, indicating the high fabrication consistency (Figure S5A), and the g_m_ change of the M^+^-fOECT was <10% even at a high bending angle of 135° (Figure S6), indicating its accommodation to tissue bending. The long-term implantation of the M^+^-fOECT (Figure 1E) in the plant model of this study (devil’s ivy) did not trigger obvious adverse responses (Figure S7; Discussion S1) because of the minimal invasiveness and good biological/mechanical compatibility of the conductive PEDOT:PSS microfiber (Young’s modulus of 580 MPa). In contrast, the implantation of a rigid stainless-steel needle of a similar diameter caused serious necrosis and the formation of a callus due to the huge mismatch between the Young’s moduli of steel and soft plant tissue (100 GPa scale vs. 10 MPa scale). If a rigid implantable device was used, then the tissue damage would lead to false observations, and the callus surrounding it would comprise the signal quality.^25^ The conductivity and g_m_ of the M^+^-fOECT also remained stable, promising long-term *in planta* monitoring (Figure 1F).

### Transpiration-driven xylem flow monitored by M^+^-fOECT

**Figure 2:**
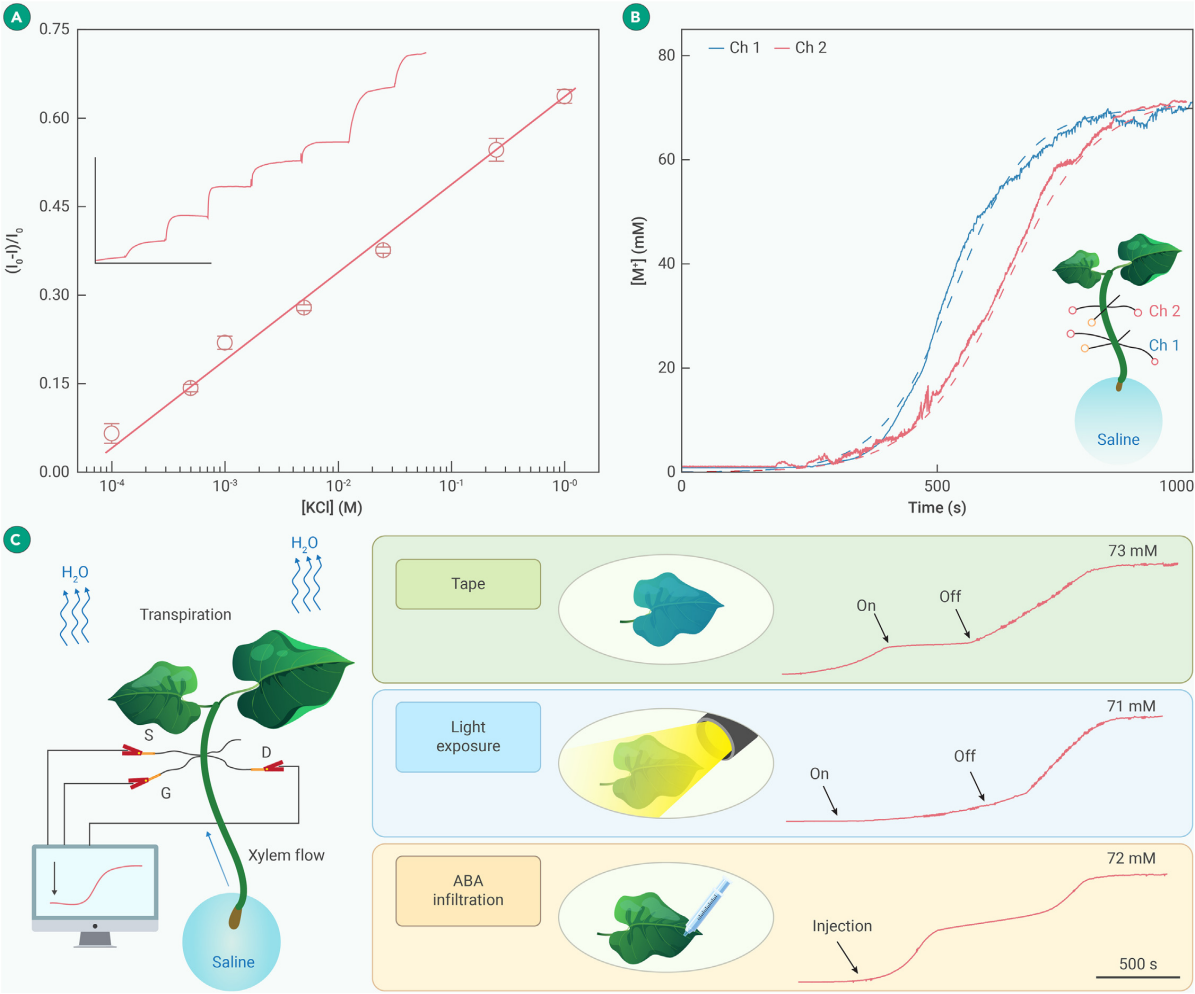
Monitoring cation variation, xylem transport, and transpiration by the M^+^-fOECT (A) Calibration curve of percentage change of I_sd_ vs. variation of K^+^ concentration obtained from 4 different fOECTs (error bars represent standard deviations). Inset shows the I_sd_ percentage change of a fOCET-M^+^ responding to the drop-wise addition of KCl solution to various concentrations (scale bar: vertical = 0.3 mA, horizontal = 500 s). (B) Cation concentration increase due to xylem flow transport after immersing the rootless plant in 75 mM KCl solution, monitored by 2 fOECTs implanted 1 and 2 cm above the solution. Dashed curves are the fittings by the 1D convection model. (C) Cation concentration variation due to transpiration-driven xylem flow measured at 2 cm above the solution responding to covering stomata using tape, exposure to simulated sunlight (200 μmol m^−2^ s^−1^), and infiltration of abscisic acid (ABA; 10 μM, 1 mL).

Currently, the cation concentration is determined using the destructive extraction of plant sap, which is not continuous and thus unable to resolve the rapid physiological fluctuation of cations. In contrast, the M^+^-fOECT can quickly respond to cation variation (Figure 2A), with the percentage of I_ds_ change being linearly proportional to the logarithmic increase of the cation concentration with a sensitivity of 116.7 μA/dec.

Plant transpiration involves water transport (together with ions and nutrients) from roots through xylem vessels driven by the negative pressure resulting from water vapor evaporation through stomata on leaves. In order to monitor transpiration-driven xylem flow in a rootless plant cultivated in 75 mM KCl solution, two fOECTs were threaded into the stem (Figure 2B). Cation concentrations at both recording sites reached an equilibrium (∼70 mM) slightly below the K^+^ concentration in the cultivation solution with a τ of ∼355 s, which is much faster than the τ calculated based on the 1D diffusion model (∼1.3 days). Thus, the fast transport of K^+^ by xylem flow shall follow the transpiration-driven 1D convection model.^26^ Given that the M^+^-fOECT reports the average K^+^ concentration in the entire stem, and assuming it quickly follows the change in the xylems due to fast lateral diffusion, the K^+^ concentration in the stem (C) as the function of position (x) and time (t) can be described by (see Figure S8 and Discussion S2)

$$\frac{\partial C\left(x,t\right)}{\partial t}=-\text{ur}\frac{\partial C\left(x,t\right)}{\partial x}\text{,}$$

where u is the xylem flow rate and r is the area percentage of xylem vessels (r = 0.012; Figure S9). The model fits the measurements from both fOCETs well with a u of ∼0.35 cm/s.

We further validated that the M^+^-fOECT can faithfully detect the changes of xylem flow rate upon adjusting transpiration levels by controlling the opening and closure of stomata (Figure 2C). The xylem flow completely halted immediately after blocking stomata by covering both sides of the leaves and was fully restored immediately after removing the covering tapes. Evidently, the fOCET-M^+^ shall enable unprecedented studies of transpiration dynamics and signaling pathways that control stomatal guard cells. As proof-of-concept demonstrations, stomatal dynamics regulated by an environmental stimulus (exposure to simulated sunlight) and plant hormone (abscisic acid [ABA]) were monitored in real time. Interestingly, light-induced stomatal opening (indicated by the sudden acceleration of the xylem flow) exhibited a long delay (∼1,000 s), while ABA-induced stomatal closure (sudden suppression of the xylem flow) showed a shorter delay (∼450 s) and subsequent recovery after an additional ∼640 s (see Figure S10 and Discussion S3). As shown in Figure S11, the 1D convection model can well fit the experimental measurements and derive the xylem flow rates under these distinct transpiration conditions, indicating the generalizability of the model and the reliability of the measurement (Discussion S4).

### Detection of H_2_O_2_ wave induced by different stressors using H_2_O_2_-fOECT

**Figure 3:**
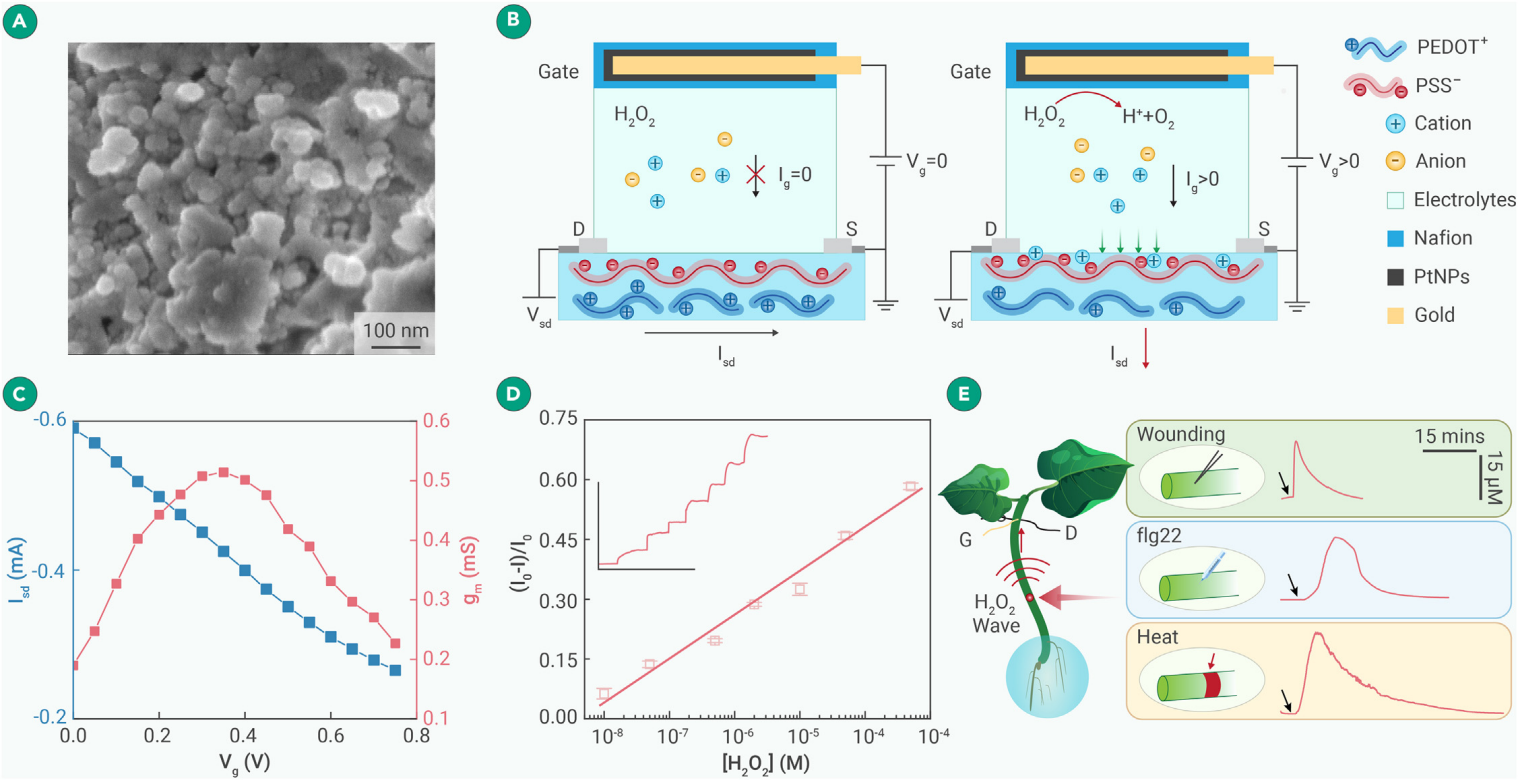
H_2_O_2_-fOECT for continuous *in planta* monitoring (A) SEM image of Pt nanoparticles coated on an Au microfiber. (B) Working mechanism of H_2_O_2_-fOECT. (C) Transconductance curve (red) and transfer curve (black) of H_2_O_2_-fOECT in 10 μM H_2_O_2_ solution. (D) Calibration curve of percentage change of I_sd_ vs. H_2_O_2_ concentration obtained from 4 different fOECTs (error bars represent standard deviations). Inset shows the responses of a fOECT upon the drop-wise addition of H_2_O_2_ solution to reach various defined concentrations (scale bars: vertical = 0.3 mA, horizontal = 1,000 s). (E) Wounding-, heat-, and flg22-induced H_2_O_2_ waves. All stimulations were applied 1 cm away from the fOECT at the time points indicated by the arrows.

To continuously monitor variation of H_2_O_2_ in plants, we devised an H_2_O_2_-responsive fOECT (H_2_O_2_-fOECT) with a PEDOT:PSS fiber as the channel and a flexible Au microfiber (250 μm) functionalized with PtNPs as the electrochemical gate.^14^ PtNPs (∼100 nm; Figure 3A) are stable and efficient electrocatalysts for the oxidation of H_2_O_2_ with high selectivity (Figure S12). Nafion coating secures PtNPs in place and prevents the capacitive accumulation of anions on the positively biased gate electrode.^27^ Otherwise, cations will be driven into the conducting channel, thereby leading to an erroneous signal, particularly when the cation concentration is high. Nafion shielding and the Faradaic reaction at the gate (see Figure 3B and the reaction equations below) allow the fOECT to selectively respond to H_2_O_2_ but not to high concentrations of cations (Figure S4B):

gate: H_2_O_2_ – 2e^−^ = O_2_ + 2H^+^ and

channel: PEDOT^+^PSS^−^ + e^−^ + M^+^ = PEDOT^0^ + PSS^−^M^+^.

In a 10 μM H_2_O_2_ solution, the H_2_O_2_-fOECT reaches the highest g_m_ of 0.53 mS at V_g_ = ∼0.35 V (Figure 3C), which is therefore used for all H_2_O_2_ recording experiments. The device-to-device variation of g_m_ is small (Figure S5B), ensuring consistent measurements. As shown in Figure 3D, the percentage increase of I_sd_ is linearly proportional to the logarithmic increase of the H_2_O_2_ concentration with a sensitivity of 0.109 mA/dec and an extrapolated low detection limit of 20 nM at S/N = 3. Evidently, the H_2_O_2_-fOECT is a reliable, sensitive, and fast device for monitoring H_2_O_2_ variation in plants.

In contrast to the current fluorescence imaging methods, the H_2_O_2_-fOECT can spatiotemporally resolve the generation and propagation of H_2_O_2_ in deep plant tissue. A sharp H_2_O_2_ wave was registered when wounding was applied 1 cm away with a short delay of 2.3 s (Figure 3E). In contrast, mild heating stress (45°C, 1 min) resulted in a relatively slow yet sustained H_2_O_2_ wave (Discussion S5). Lastly, the H_2_O_2_ response triggered by the flg22 peptide^28^ (the most conserved domain of bacterial flagellin) exhibited the slowest kinetics due to the intricate and protracted signaling pathways involved (see Figure S13 and Discussion S5). Evidently, H_2_O_2_-fOCET can serve as a unique tool to scrutinize the H_2_O_2_ signaling kinetics and pathways.

### Interdependence of H_2_O_2_ wave and VP signal and stress-intensity dependence

It is well accepted that H_2_O_2_ production relies on respiratory burst oxidase homolog D (RBOHD), which can be activated by stressors and Ca^2+^.^6^^,^^29^^,^^30^^,^^31^ It is also well recognized that H_2_O_2_ opens Ca^2+^ channels^32^^,^^33^^,^^34^ and Ca signaling is coupled with VP.^4^^,^^31^^,^^35^ Several theories have been proposed to explain the propagation of the H_2_O_2_ wave: (theory I) the H_2_O_2_ burst triggers RBOHDs on neighboring cells to produce the subsequent H_2_O_2_ burst (self-propagating),^6^^,^^36^^,^^37^ (theory II) propagating VP opens voltage-dependent Ca^2+^ channels along the way, and the generated Ca^2+^ signal produces propagating H_2_O_2_ waves,^3^^,^^38^ and (theory III) Ca^2+^ travels through symplastic or apoplastic routes and initiate H_2_O_2_ regeneration in neighboring cells.^3^^,^^39^ The mechanism of VP generation and propagation is also under debate. It has been hypothesized that VP is self-regenerative and propagates similarly to the action potential in neurons (hypothesis A),^40^ the hydraulic pressure wave generated in the stimulated zone activates mechano-sensitive ion channels and drives the regeneration and propagation of VP (hypothesis B),^41^^,^^42^ and the produced stress substances travel through xylem vessels and stimulate the regeneration of VP along the way (hypothesis C).^41^^,^^43^

**Figure 4:**
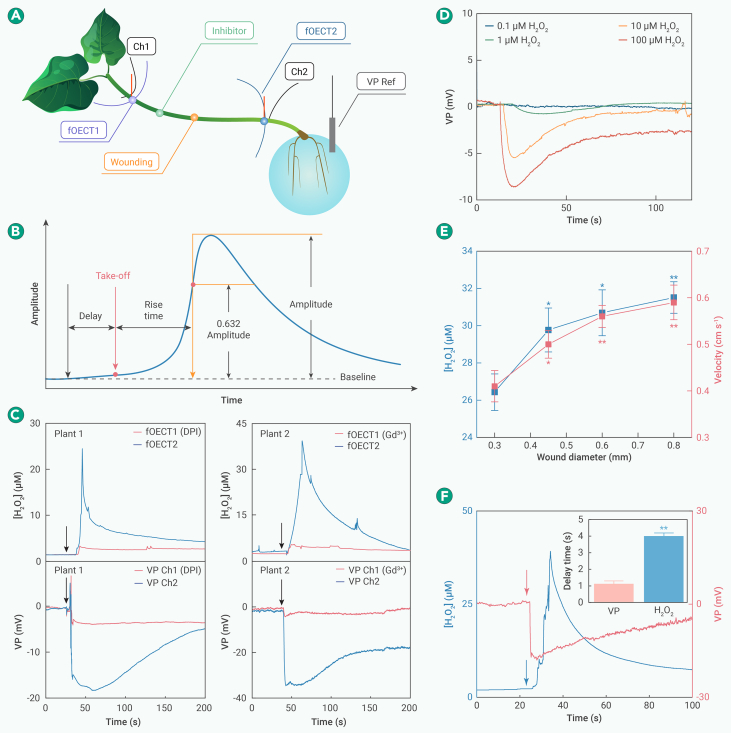
Interdependence of H_2_O_2_ waves and VP (A) H_2_O_2_ and VP waves recorded by two H_2_O_2_-fOECTs (2 cm apart) and two PEDOT:PSS microfibers (Ch1 and Ch2) adjacent to the fOECTs, respectively, in response to wounding inflicted in the middle of the two recording sites after inhibitor (DPI or Gd^3+^) was infused in the middle of wounding site and fOECT1. (B) Definition of kinetic characteristics. Black arrow: stimulation time, red arrow: take-off point (signal = 5× of baseline fluctuation), and yellow arrow: time point when signal reaches 1−1/e (0.632) of the peak. (C) VP and H_2_O_2_ waves simultaneously recorded on the same plant after applying DPI or Gd^3+^. (D) Exogenous H_2_O_2_ (100 μL) induced VP. (E) Amplitude and velocity of H_2_O_2_ waves depend on stress intensity and are positively correlated with each other. H_2_O_2_ waves were recorded by two fOECTs (1 cm apart), responding to wounding (1 cm away from the first fOECT) inflicted by needles of different diameters. The velocity is calculated based on the time difference between the take-off points of H_2_O_2_ waves recorded by the fOECTs. (F) Simultaneous measurement of VP and H_2_O_2_ waves induced by the same wounding that was applied 1 cm away. Inset shows statistics of the delay time, which is defined as the duration from the moment of wounding to the take-off point. The data in (E) and (F) (inset) are presented as mean ± SD (*n* = 3 plants) and analyzed using a one-sample t test (∗*p* < 0.05 and ∗∗*p* < 0.01).

To investigate these conjectures, we selectively blocked proteins that are essential to H_2_O_2_ and VP generation and measured both signals on the same plants with the microfiber electronics. Two H_2_O_2_-fOECTs were threaded through the plant stem, and wounding was inflicted in the middle between the fOECTs after infusing an RBOHD inhibitor (DPI) through a previously created incision halfway between fOECT1 and the wounding site (Figure 4A). The kinetics of H_2_O_2_ and VP waves was analyzed based on the characteristics defined in Figure 4B. fOECT2 registered a strong H_2_O_2_ wave, whereas the propagation of the H_2_O_2_ wave to fOECT1 was blocked because of the inhibition of RBOHD on its pathway. Two PEDOT:PSS microfibers implanted adjacent to the fOECTs were used to simultaneously record VP in response to the same wounding applied in the middle. Similarly, VP was only recorded at fOECT2 but not fOECT1 (Figure 4C), indicating that blocking RBOHD-dependent H_2_O_2_ generation also prevents the generation and propagation of VP.

It is known that Ca^2+^ influx through H_2_O_2_-activable Ca^2+^ channels elicits the internal release of Ca^2+^ through two-pore cation channels (TPC1) on vacuoles.^44^ Subsequently, membrane depolarization is produced because the rapidly increased cytosol Ca^2+^ level inhibits the out pumping of H^+^ by H^+^-ATPase and induces Cl^−^ efflux through anion channels.^31^^,^^45^ The depolarization of many plant cells collectively produces a VP wave. In a similar experiment, Gd^3+^ (a Ca^2+^ channel blocker) was introduced between fOECT1 and the wounding site. It was observed that the propagation of both the H_2_O_2_ wave and VP to fOECT1 was obstructed (Figure 4C), suggesting that blocking VP prevents the propagation of the H_2_O_2_ wave. Note that the infusion of a KCl solution did not prevent the generation and propagation of either the VP or H_2_O_2_ wave (Figure S14), ruling out the negative impact by infusion itself. Further, a VP-mimicking voltage pulse did not affect the wounding-induced H_2_O_2_ wave (Figure S15), suggesting that the accompanying VP does not electrically interfere with the H_2_O_2_ recording by the fOECT (see Discussion S6). A direct comparison of paired recordings on the same plant and the fact that all the observed signals are dynamic responses to a specific physiological context rule out the possibility of false observations due to interferences. Moreover, the simultaneous recording of VP and H_2_O_2_ waves on the same plant unambiguously demonstrates the interdependence and intimate coupling between the two waves.

The observed interdependence of H_2_O_2_ and VP waves denies hypothesis A for VP and theory I for H_2_O_2_, and theory I is also challenged by the fact that the intercellular diffusion of H_2_O_2_ is largely prevented by the abundant existence of antioxidants in apoplast.^46^ Moreover if theory II for H_2_O_2_ were true, then wounding-triggered self-propagating VP should guarantee H_2_O_2_ propagation through the RBOHD-blocked site. Further, the observed critical involvement of Ca^2+^ channels for H_2_O_2_ propagation is not reflected in H_2_O_2_ theory III.

To further investigate whether H_2_O_2_ is sufficient for VP generation, we recorded VP waves when H_2_O_2_ of various amounts was infused at 1 cm away (Figure 4D). Interestingly, exogeneous H_2_O_2_ can not only trigger VP but also determines the amplitude and rising kinetics of VP in a dose-dependent manner, suggesting a stress-intensity dependence. This experiment denies VP hypothesis B because H_2_O_2_ stimulation alone (without the exertion of pressure) can trigger propagating VP.

Conceivably from Figure 4D, a higher H_2_O_2_ concentration leads to wider spreading of H_2_O_2_, hence evoking a stronger VP as the collective response from more plant cells. Also conceivably, the stronger the initially triggered VP, the faster speed it travels because it more potently triggers the regeneration mechanism that involves both RBOHD and Ca^2+^ channels. Viewing the intimate coupling between H_2_O_2_ and VP generation, we further hypothesize that stress intensity also correlates with H_2_O_2_ wave propagation. To investigate our hypothesis, H_2_O_2_ waves were recorded by two fOECTs threaded into the plant (1 cm apart) upon wounding with needles of different diameters. As expected, both the amplitude and propagation velocity positively scale with the stress intensity (Figure 4E). In addition, the amplitude and velocity are positively correlated with each other, suggesting that the propagation kinetics depends on signal intensity.

Subsequently, we conducted simultaneous recordings of wounding-induced VP and H_2_O_2_ waves at the same location to compare their kinetics (Figure 4F). VP and H_2_O_2_ are closely accompanied by each other. The former is always faster than the latter and rises more steeply, presumably because H_2_O_2_ generation is a slower process involving the RBHOD-catalyzed reaction. Both VP and H_2_O_2_ waves have a similar long-lasting tail following the rapid rise. Altogether, our observations suggest that the generations of H_2_O_2_ and VP are interdependent and mutually reinforcing, with the latter having a faster kinetics (faster rise and velocity), and that the amplitude and propagation velocity of both VP and H_2_O_2_ waves positively correlate with the initial stress intensity.

### Influence of xylem flow in H_2_O_2_ and VP propagation

**Figure 5:**
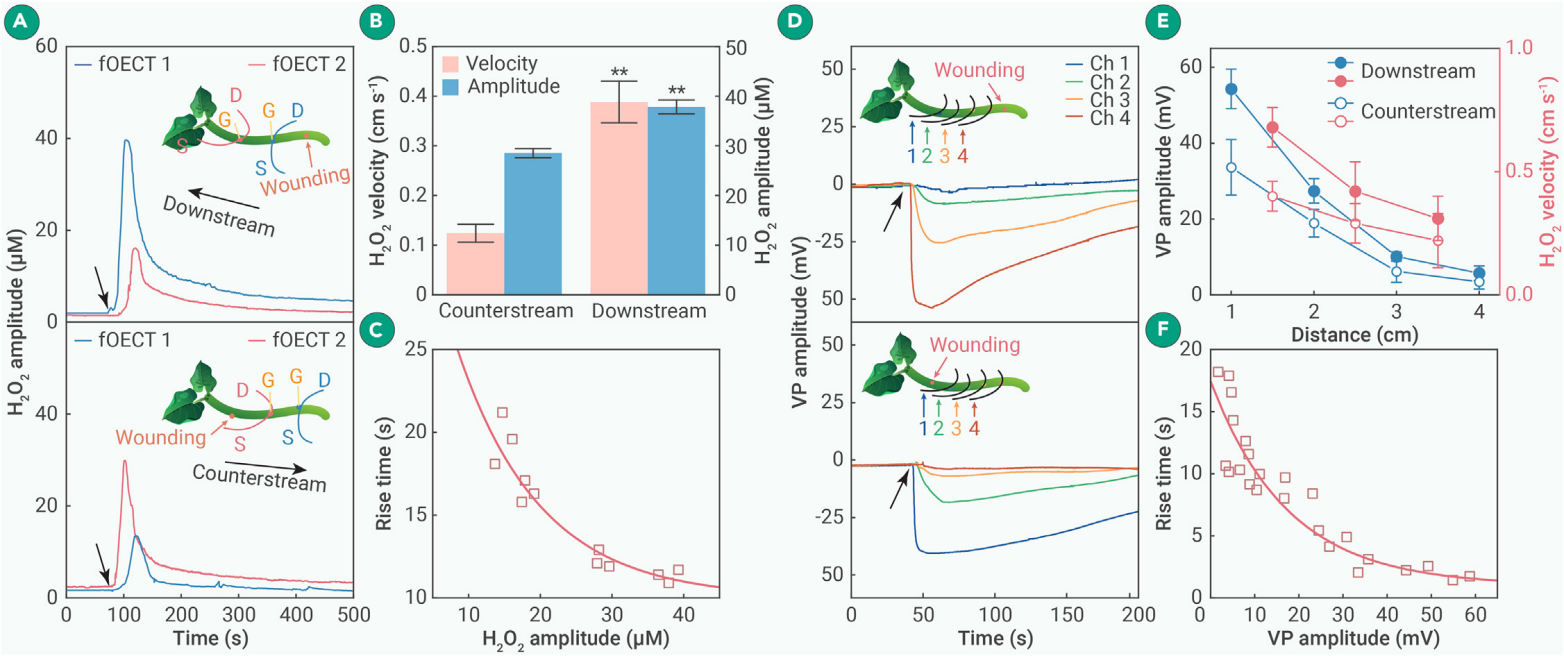
Influence of xylem flow in H_2_O_2_ and VP propagation (A) The H_2_O_2_ waves elicited by wounding applied either 1 cm below or above H_2_O_2_-fOECTs. (B) H_2_O_2_ wave amplitude and velocity in downstream and counterstream directions. (C) Rise time decreases with H_2_O_2_ amplitude. (D) VP waves elicited by wounding applied either 1 cm below or above 4 PEDOT:PSS microfibers. (E) VP amplitude and velocity decay with distance in both directions. (F) Rise time decreases with VP amplitude. The data in (B) are presented as mean ± SD (*n* = 3 plants) and analyzed using a one-sample t test (∗∗*p* < 0.01 vs. counterstream).

To investigate the influence of transpiration-driven xylem flow on the propagation of H_2_O_2_ waves, wounding-induced signals traveling downstream (toward leaves as xylem flow) or counterstream were recorded by two H_2_O_2_-fOECTs implanted 1 and 2 cm away from the wounding site (Figure 5A). The downstream velocity was substantially higher than the counterstream velocity (0.39 vs. 0.16 cm/s; Figure 5B), suggesting that H_2_O_2_ transport by xylem flow greatly promotes the propagation of H_2_O_2_ waves beyond passive diffusion. Even the counterstream velocity is too fast to be explained by H_2_O_2_ theory I which attributes H_2_O_2_ propagation to slow H_2_O_2_ spreading alone. Our observation suggests the importance of VP to reinforce and accelerate H_2_O_2_ propagation. The downstream velocity is similar to the xylem flow rate determined by the M^+^-fOECT, implying the dominating influence of the xylem flow in downstream propagation. The H_2_O_2_ amplitude is also significantly smaller in the counterstream direction, further corroborating the correlation between propagation kinetics and signal intensity. The direction dependence of H_2_O_2_ propagation cannot be explained by H_2_O_2_ theory I, II, or III. Theory III is also questioned by the fact that apoplastic diffusion of Ca^2+^ is largely prevented by its binding with abundant carboxyl groups from pectin on cell wall.^47^ It has been demonstrated that Ca^2+^ can reach adjacent cells by symplastic transportation via plasmodesmata (PDs),^39^^,^^48^^,^^49^ which explains the counterstream regeneration and propagation of H_2_O_2_ waves. The rise time of H_2_O_2_ waves measured at various distances in both directions shows a robust negative correlation with its amplitude (Figure 5C), further strengthening our hypothesis of kinetic dependence on intensity. The exponential extrapolation suggests that the shortest possible rise time is ∼9.9 s, indicating the kinetic limit of H_2_O_2_ generation.

To investigate the influence of xylem flow on VP propagation, four PEDOT:PSS microfibers were threaded through the plant stem with equal spacing of 1 cm. VPs were sequentially registered by these microfibers after wounding being applied first downstream then counterstream at 1 cm away from the nearby fiber (Figure 5D). VP travels notably faster in the downstream direction (Figure 5E), suggesting that VP propagation is greatly facilitated by H_2_O_2_ transport by the xylem flow. The VP velocity (up to 0.68 cm/s) is much slower than a pressure wave (up to 1,500 m/s),^50^ disproving VP hypothesis B. The observed direction-dependent propagation cannot be explained by VP hypothesis A or B. Note that hypothesis C, which explains VP propagation by the transport of stress substances through xylem vessels, is not able to explain its counterstream propagation. Conceivably, it is Ca^2+^ ions transported from the already excited cells to the counterstream cells that are responsible for triggering the H_2_O_2_-VP mutual-reinforcing generation and propagation in the counterstream direction. However, the critical involvement of the Ca^2+^ channel (Figure 4C) suggests that Ca^2+^ ions through PDs are not sufficient to trigger the mutual-reinforcing loop. Plausibly, they activate RBOHDs, which in turn open Ca^2+^ channels to admit more Ca^2+^ ions. Thereby, the abundant release of Ca^2+^ from vacuoles becomes possible, permitting the regeneration of H_2_O_2_ and VP wave events in the counterstream direction.

Both the amplitude and propagation velocity of H_2_O_2_ and VP waves exponentially decrease with travel distance (Figures 5E and S16) with similar decay constants (2.55 vs. 2.54 cm for velocity decay and 1.53 vs. 1.29 cm for amplitude decay). Such similar decay indicates that the regeneration and propagation of both waves are intimately coupled, and the observed distance decay challenges H_2_O_2_ theory II and VP hypothesis A. Similar to H_2_O_2_ waves, the rise time of VP shows a strong negative correlation with its amplitude (Figure 5F). The extrapolated minimum of 0.72 s indicates the kinetic limit of VP generation, which is faster than that of H_2_O_2_.

### The proposed new mechanism

**Figure 6:**
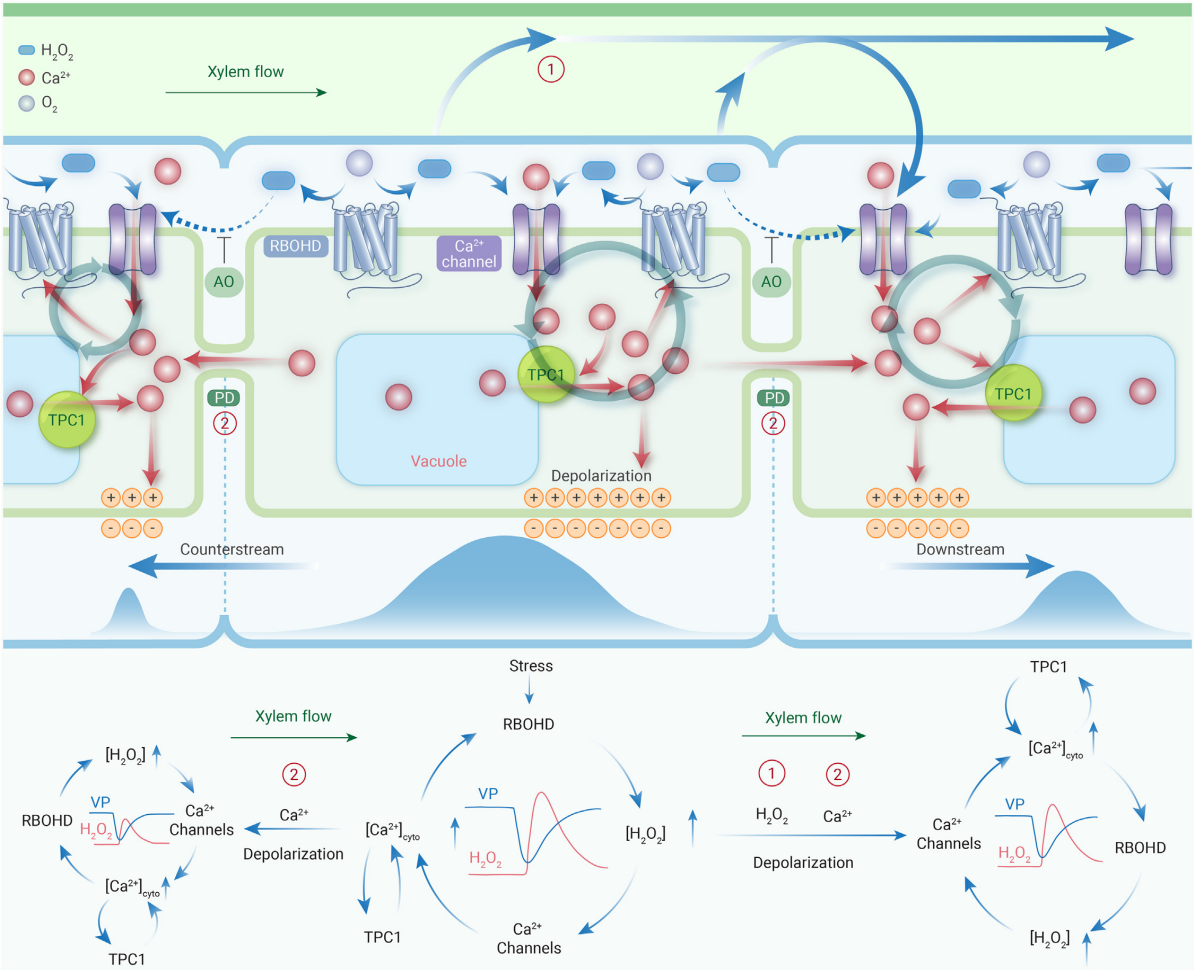
Illustration of transpiration-dependent H_2_O_2_-VP mutual-reinforcing propagation ① and ② indicate xylem transport of H_2_O_2_ and Ca^2+^ transport through PDs, respectively. AO, antioxidant; TPC1, the two-pore channel 1; PDs, plasmodesmata.

Based on our experimental results and some well-recognized notions, we propose a transpiration- and intensity-dependent H_2_O_2_-VP mutual-reinforcing propagation mechanism (Figure 6). Specifically, stress-stimulated RBOHDs produce H_2_O_2_, which subsequently activates Ca^2+^ channels, and the resulting Ca^2+^ influx stimulates internal Ca^2+^ release from vacuoles through TPC1 channels. The increased intracellular Ca^2+^ further stimulates RBOHDs to produce more H_2_O_2_ and causes membrane depolarization by inhibiting the out pumping of H^+^ and inducing Cl^−^ efflux. Therefore, a positive-feedback loop between H_2_O_2_ production and membrane depolarization is established, ensuring their fast development. The synchronized excitation of many cells collectively produces H_2_O_2_ and VP waves. The kinetics of the former slightly lags behind because of the involvement of biochemical reactions. Stronger stress leads to more cells being excited, hence the higher wave amplitudes.

Transported by xylem flow, the produced H_2_O_2_ spreads to the downstream cells to trigger the mutual-reinforcing regeneration of H_2_O_2_ and VP waves. H_2_O_2_ is difficult to reach adjacent cells through apoplastic pathway due to the abundant presence of antioxidants (e.g., ascorbic acid). This disapproves theory I for H_2_O_2_ propagation and suggests that the counterstream propagation of VP and H_2_O_2_ waves is not dependent on the apoplastic diffusion of H_2_O_2_. On the other hand, Ca^2+^ ions can reach both downstream and counterstream cells via PDs, thereby reinforcing the downstream generation of both waves and enabling their counterstream regeneration. Ca^2+^ channels are critical to the H_2_O_2_-VP mutual-reinforcing (positive-feedback) loop. In the counterstream direction, they are opened by PD-transported Ca^2+^ ions through RBOHD mediation. However, further studies are required to unambiguously test this PD-dependent hypothesis.

The higher the amplitude of the H_2_O_2_ or VP wave, the faster its kinetics (faster propagation velocity and rise time) because the mutual-reinforcing loop is more potently evoked and more messengers (H_2_O_2_ and Ca^2+^) reach the neighboring cells. Due to the long-distance H_2_O_2_ signaling enabled by the xylem flow, the downstream waves exhibit higher amplitude and velocity, but even the counterstream velocity of both waves is too fast to be explained by the mechanisms purely relying on the diffusion of H_2_O_2_ (theory I) or Ca^2+^ (theory III), suggesting that H_2_O_2_-VP mutual reinforcement is critical to the fast generation and propagation of both waves. In both directions, the amplitudes and velocities of the regenerated H_2_O_2_ and VP waves concomitantly decay with distance.

By simultaneously recording VP and H_2_O_2_ waves and recording on different locations on the same plant with high temporospatial resolution, we provide strong and direct evidence to show that (1) H_2_O_2_ and VP waves are critically interdependent (i.e., inhibition of one prevents the other) and (2) they are intimately coupled (i.e., coupled kinetics, similar directional dependence, similar decay with distance, amplitude correlation, and H_2_O_2_ alone can stimulate VP in a dose-dependent manner). The herein proposed mechanism can fully explain all of our observations, whereas the previously proposed theories or hypotheses are denied by multiple observations from our experiments.

## Conclusion

The flexible microfiber-shaped OECT offers a versatile platform for the *in situ* study of dynamic physiological processes in plants with high temporospatial resolution. Using two different gate electrodes, cation-responsive and H_2_O_2_-responsive fOECTs were herein devised. The gate can be otherwise engineered to selectively sense a range of chemicals or molecules, for example, incorporating redox enzymes for detecting their substrates. Microfiber electronics presents distinct advantages over conventional imaging-based tools, including sub-second temporal resolution, the capacity for deep-tissue exploration, and a chronically stable bioelectronic interface for longitudinal studies.

Utilizing microfiber electronics to record H_2_O_2_ and VP waves on the same plants, we unveiled the fundamental mechanisms underlying these dynamic processes, revealing an interdependent and mutual-reinforcing positive-feedback loop and intensity-dependent kinetics. Moreover, we discovered that both H_2_O_2_ and VP waves propagate faster toward the leaves than toward the roots because of the directional long-distance transport of H_2_O_2_ in the xylem.

The herein proposed transpiration- and intensity-dependent H_2_O_2_-VP mutual-reinforcing propagation theory shall inspire further research on the coordinated response to stresses and long-distance signaling enabled by xylem flow. In addition, the observed kinetic differences of distinct signaling cascades that control stomatal opening/closing and H_2_O_2_ wave generation are carefully discussed in the supplemental information. However, the observed interesting phenomena warrant further investigation. We envision that by combining microfiber electronics with molecular and genetic approaches, the cascaded signaling pathways underlying various physiological processes in plants can be precisely deciphered.

## Data and code availability

Data are available from the corresponding author upon reasonable request.

## Acknowledgments

H.W. acknowledges the research scholarship awarded by the Institute of Flexible Electronics Technology of Tsinghua, Zhejiang (IFET-THU); 10.13039/501100001475Nanyang Technological University (NTU); and Qiantang Science and Technology Innovation Center, China (QSTIC). This project is supported by the Singapore Indoor Farming System (SingFarm) CREATE Initiative (024574-00005) from the 10.13039/501100001381National Research Foundation of Singapore, and a 10.13039/501100019679National University of Singapore Presidential Young Professorship Award (22-4974-A0003).

## Author contributions

P.C., H.W., and Y.L. conceived the project, designed the study, and wrote the manuscript. H.W. performed the majority of experiments and data analysis. L.K. assisted with the experiments and data analysis. X.Z. and Y.M. provided plant reagents (e.g., *Flg22*) and helped with result discussions. X.S., Y.M., and X.C. assisted with the experimental design and discussions. All authors have revised the manuscript and given their approval of the final version.

## Declaration of interests

The authors declare no conflicts of interest.

## Supplemental information

Document S1. Figures S1–S16 and Discussions S1–S6

Document S2. Article plus supplemental information
