# Supplementary material for: Mutually reinforcing and transpiration-dependent propagation of H2O2 and variation potential in plants revealed by fiber organic electrochemical transistors
Source: Innovation (Camb). 2025 Jan 6;6(5):100800. doi: 10.1016/j.xinn.2025.100800 (PMC12105492; doi:10.1016/j.xinn.2025.100800)
Supplement: Document S1. Figures S1–S16 and Discussions S1–S6 [file mmc1.pdf]

The Innovation, Volume *6*

Supplemental Information

Mutually reinforcing and transpiration-dependent propagation of H_2O_2 and variation potential in plants revealed by fiber organic electrochemical transistors

Hanqi Wen, Lingxuan Kong, Xinlu Zhu, Yansong Miao, Xing Sheng, Xiaodong Chen, Yuxin Liu, and Peng Chen

Supplemental Information

Mutually reinforcing and transpiration-dependent propagation of H_2O_2 and variation potential in plants revealed by fiber organic electrochemical transistors

Hanqi Wen, Lingxuan Kong, Xinlu Zhu, Yansong Miao, Xing Sheng, Xiaodong Chen, Yuxin Liu, Peng Chen

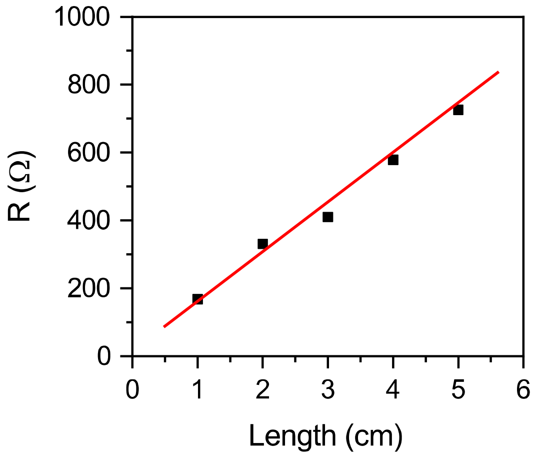

Figure S1. Resistance of the conductive microfiber linearly scales with the length, indicating the uniformity of PEDOT:PSS coating.

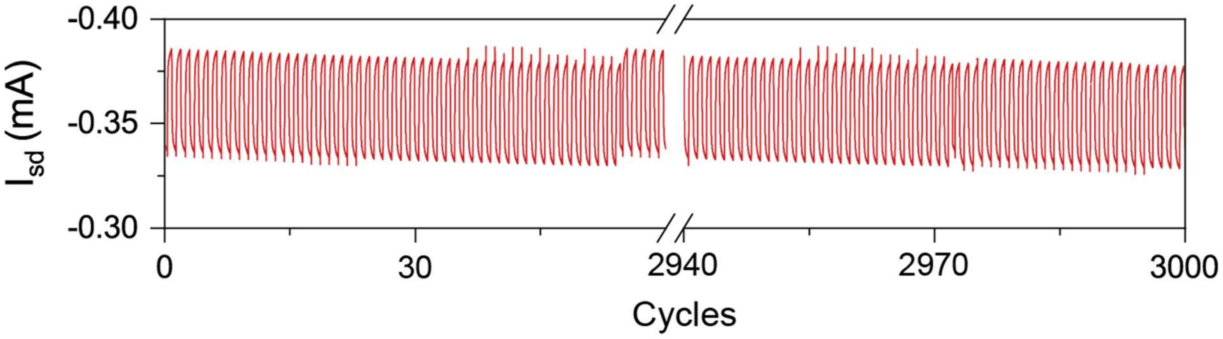

Figure S2. Stability of fOECT-M$^+$ after 3000 working cycles. With V_{sd} = -0.2 V, pulsed V_g (amplitude: 0.2 V; pulse width: 1s) was applied for 3000 times, in 5 mM KCl solution.

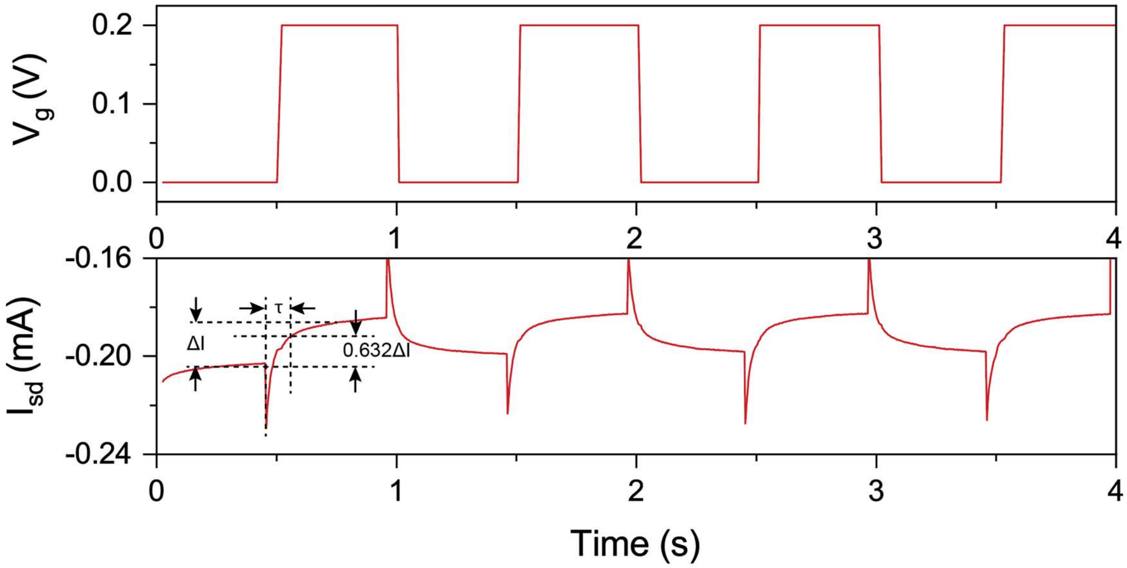

Figure S3. Response time of fOECT-M$^+$. fOECT was tested in 5 mM KCl which is typical cation concentration in plant stem tissue. Upon V_g of 0.2 V being applied, a response time constant τ of 0.11s was observed, which is defined as when signal increases by (1-1/e)*100% or 63.2%.

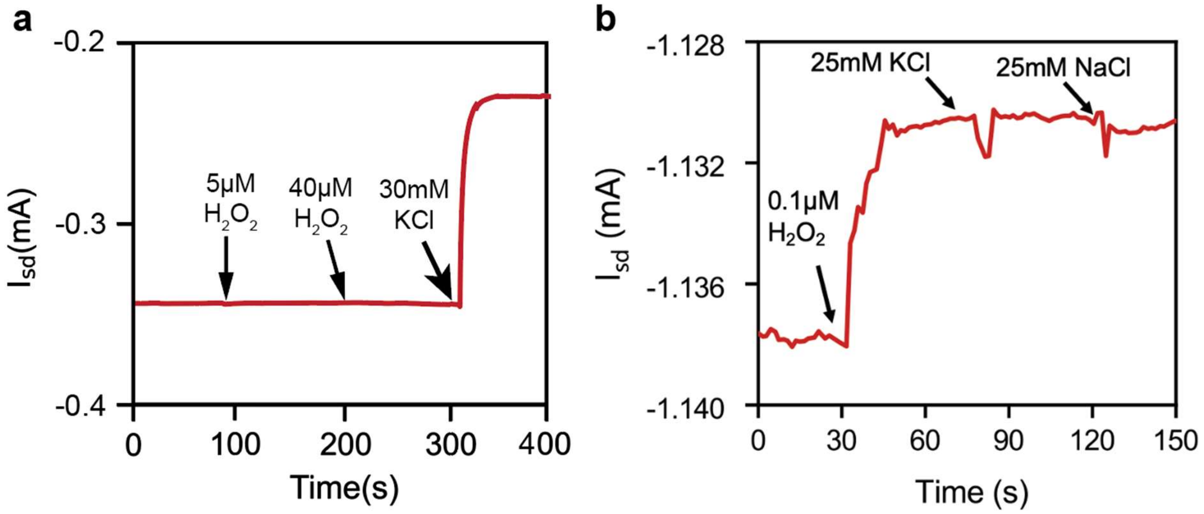

Figure S4. No crosstalk between M^+-fOECT and H_2O_2-fOECT. a, M^+-fOECT is insensitive to H_2O_2**. b,** H_2O_2-fOECT is insensitive to cation.

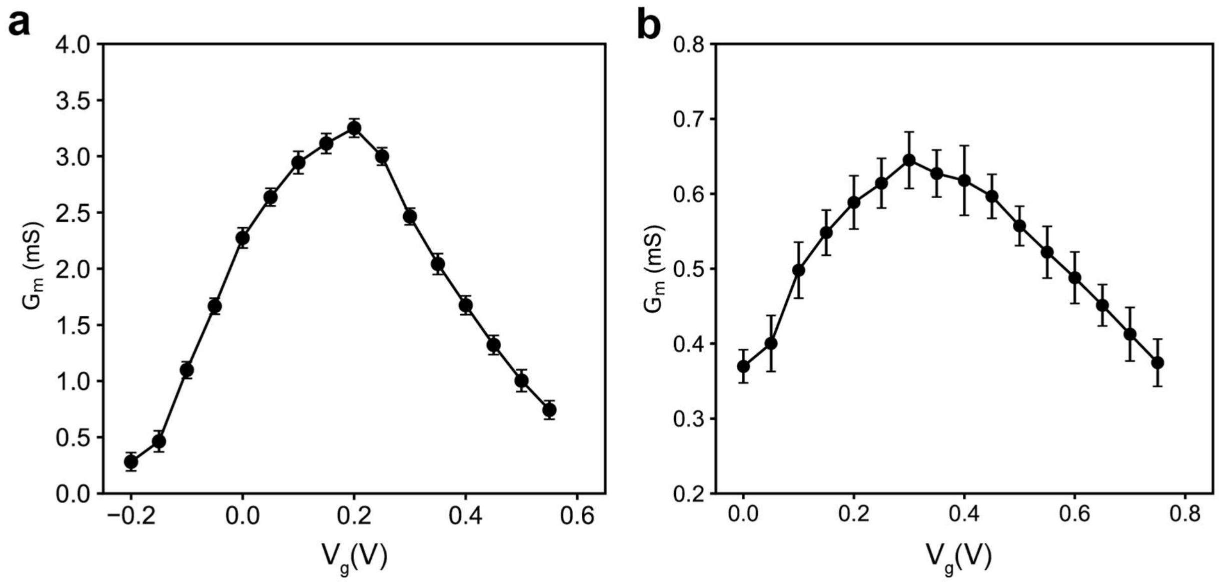

Figure S5. Averaged transconductance curve from five a) M^+-fOECTs and b) H_2O_2-fOECT. For a, fOECTs were tested in 75 mM KCl; for b, fOECTs were tested in 10 µM H_2O_2 solution. The error bars indicate the standard deviations.

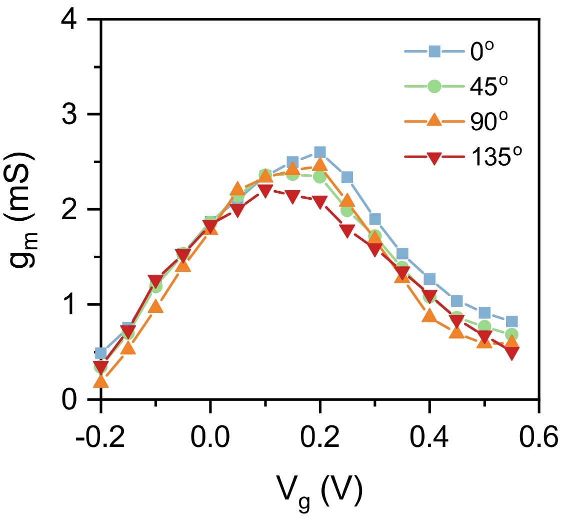

Figure S6. **Bending stability of fOECT-M⁺**. Transconductance of fOECT (g_m) when the source-drain channel fiber was bent at different angles, in 5 mM KCl solution.

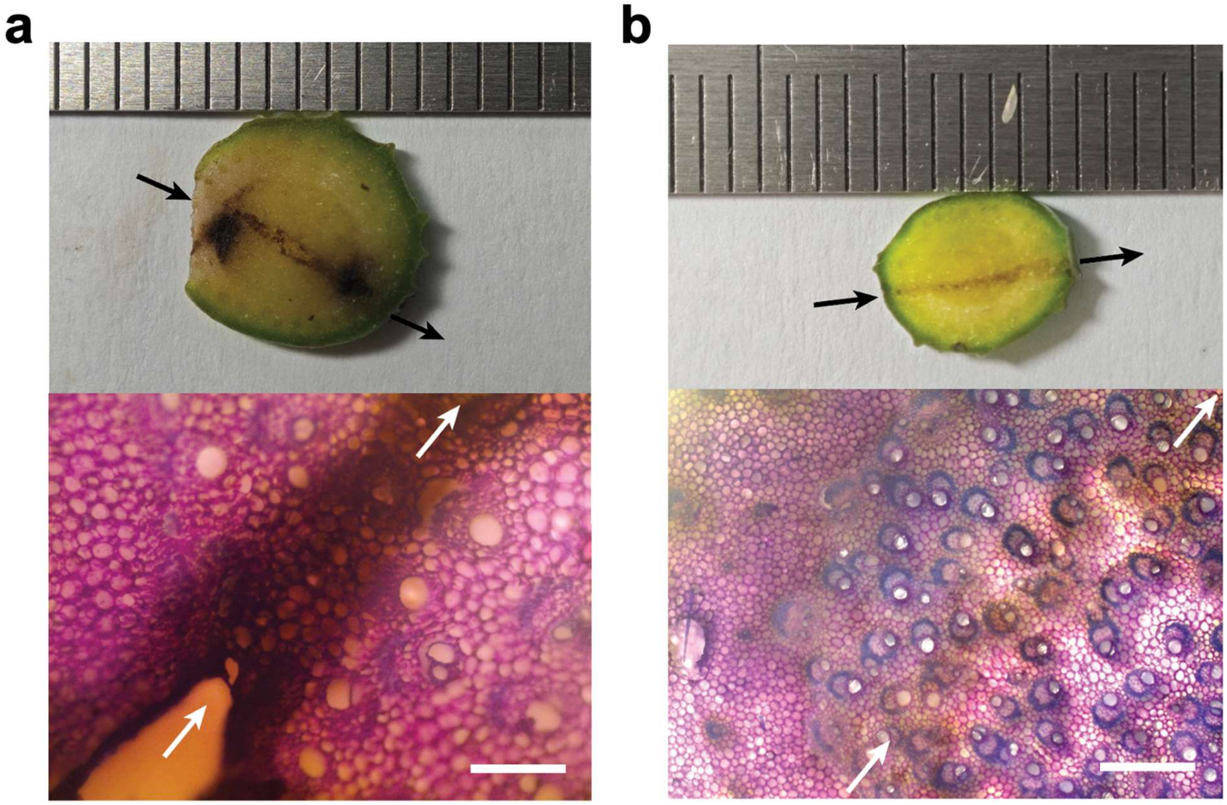

Figure S7. Plant (devil's ivy) reaction to implantation of rigid stainless steel needles (a) and flexible PEDOT:PSS microfibers (b) for 14 days. The cross section of the plan stem where the implantation was applied across it was shown by photograph (top) and micrograph after being stained by toluidine blue O (TBO). Both the microfibers and needlea have a diameter of ~240 μm.

Supplementary discussion 1: Mechanical compatibility of the conductive microfiber is important

Due to the huge mismatch in the Young's modulus (100 GPa scale vs. 10 MPa scale), the plant reacted drastically to the rigid needle as evidenced by necrosis (dark regions in the photograph) and formation of callus (deep purple in TBO staining image). Callus is a mass of undifferentiated plant cells that forms in response to injury or stimulation, serving a protective role by encasing foreign object, sealing the wound, and preventing infection. The serious damage of the plant shall likely lead to false observations. In addition, the

encapsulation of the electrode or device by the callus shall compromise the quality of the signal recording. In contrast, the PEDOT:PSS microfiber, which has a Young’s modulus (580 MPa) comparable to the plant tissue, only caused minimal cell response. Specifically, no necrosis and callus were observed. The pale brown line seen in both the photograph and staining image is due to the oxidation of phenolic compounds which are released from the damaged cells while implanting.

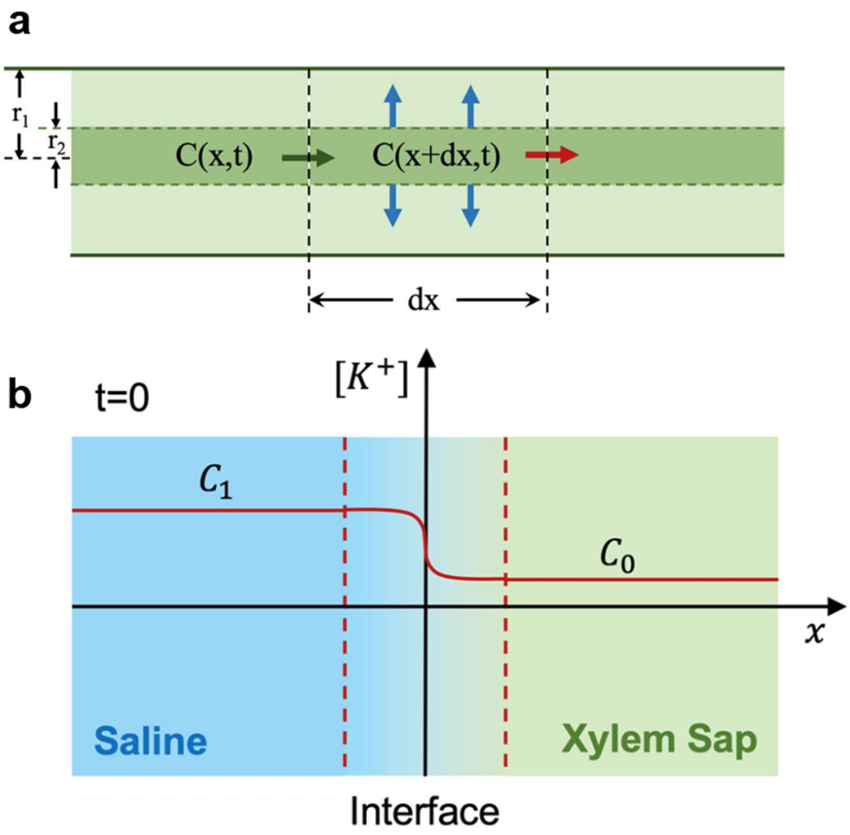

Figure S8. Modelling of xylem flow. a, 1D convection model. **b,** Boundary condition.

Supplementary discussion 2: Modelling of xylem flow

Xylem flow is described by 1D convection flow (Supplementary Figure 8a), where r_1 is the radius of the plant stem, r_2 is the effective radius of all xylem vessels (lumped into one vessel), C is the cation concentration as the function of both position x and time t. At position x+dx, the cation flow into the xylem vessel ([in]) and the cation flow out of the xylem vessel ([out]) are

$$[In] = \pi r_2^2 uC(x,t)dt$$

$$[Out] = \pi r_2^2 uC(x+dx,t)dt$$

Assuming cation concentration in xylem is quickly averaged throughout the entire stem by lateral diffusion, the concentration change at x+dx is:

$$dC = \frac{[In] - [Out]}{\pi r_1^2 dx} = \frac{\pi r_2^2 u C(x,t)dt - \pi r_2^2 u C(x+dx,t)dt}{\pi r_1^2 dx}$$

equivalently:

$$\frac{\partial C}{\partial t} = -u\frac{r_2^2}{r_1^2}\frac{\partial C}{\partial x} \tag{1}$$

At the interface between the stem and 75 mM KCl solution (x = 0):

$$C(x<0, t=0) = 75mM$$

$$C(x>0, t=0) = 2mM\ (typical\ cation\ concentration\ of\ the\ plant)$$

However, the above step function is non-analytic. Therefore, a sigmoid function was adopted to approximate the boundary condition (Figure S8b):

$$C(x,0) = \frac{C_1 - C_0}{1 + e^{\frac{x}{x_0}}} + C_0$$

where, C_0 = 2 mM, C_1 = 75 mM, and x_0 indicates the thickness of the transition region.

The analytic solution of equation (1) is:

$$C(x,t) = \frac{C_1 - C_0}{1 + e^{\frac{x-kut}{x_0}}} + C_0 \tag{2}$$

where k = $r^2{}_2/ r^2{}_1$, i.e., the area percentage of xylem vessels within the stem. This equation was used to fit our experimental data in Fig. 2b to determine xylem flow rate u.

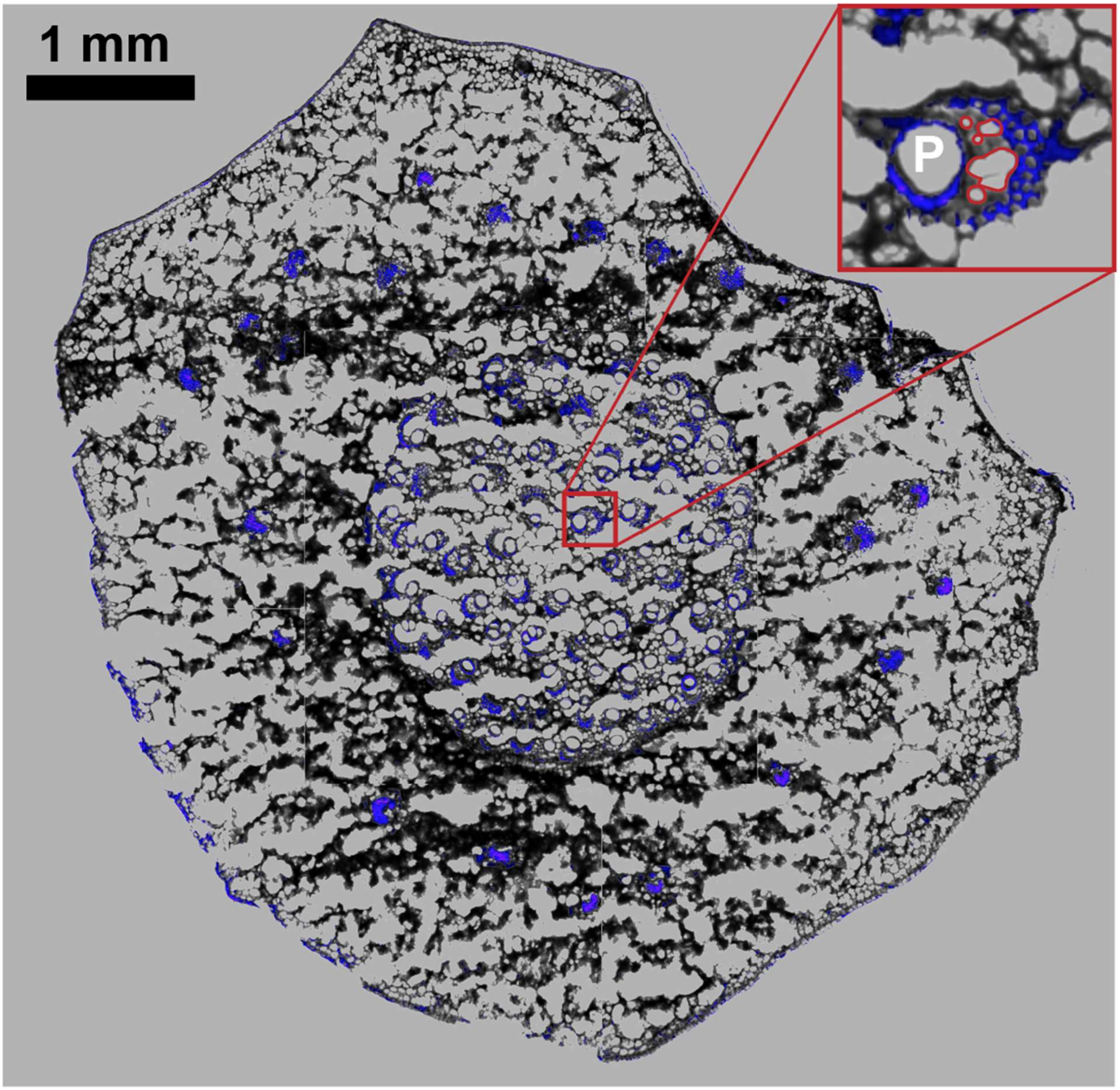

Figure S9. Superimposition of confocal fluorescence image and bright field image of plant stem cross section. Lignin is a hydrocarbon polymer enriched in vascular tissue and epidermis of plant. It exhibits fluorescence with the emission peak around 360 nm. Confocal fluorescence image shows distribution, shape and size of xylem vessels. Inset shows phloem vessel (P) and xylem vessels (red closed curves) in a vascular bundle.

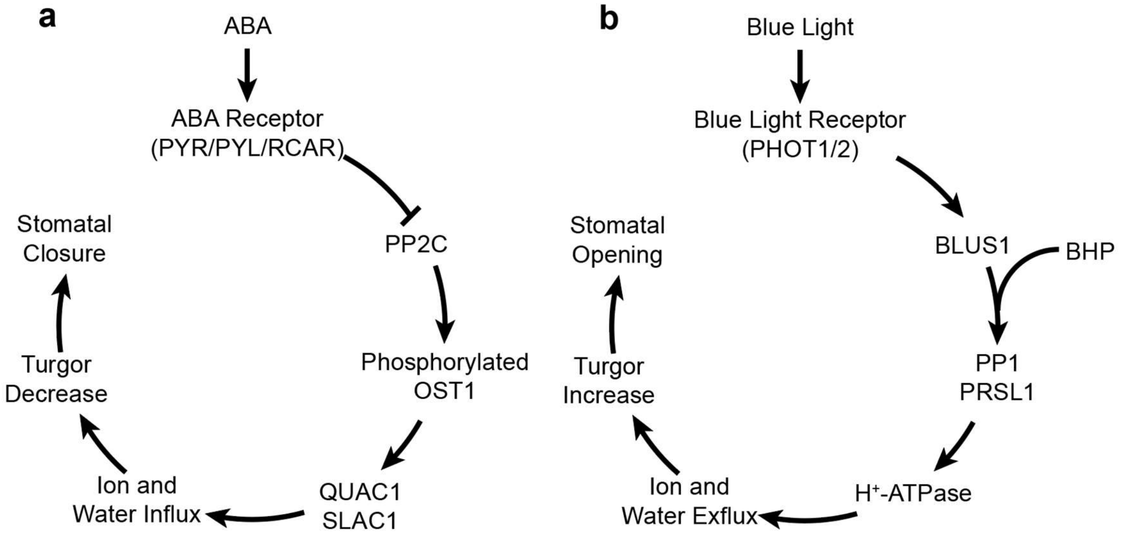

Figure S10. Signaling pathways for stomatal regulation. a, ABA induced stomatal closure. **b,** blue light induced stomatal opening.

Supplementary discussion 3: Light and ABA mediated signaling pathways for transpiration

As shown in Figure 2c, a sudden suppression of xylem flow due to stomatal closure was observed ~450s after infiltration of ABA into leaves. With additional ~640s delay, a rapid recovery of xylem flow due to stomatal re-opening was observed. The significant delay of ABA-induced stomatal closure can be explained by the underlying long signalling cascade involved (Supplementary Fig. S10a). Briefly, binding of ABA with its receptors (PYR/PYL/RCAR) inhibits type 2C protein phosphatases (PP2C). Consequently, oligosaccharyl transferase 1 (OST1) remains phosphorylated to continuously activate cation channels (QUAC1 and SLAC1). The consequent cytosolic loss of cations leads to water efflux, thereby leading to turgor decrease, and finally stomatal closure[1]. The sudden recovery of xylem flow after a long delay reveals the kinetics of the turn-off mechanisms for re-opening of stomata which may involve disabling of ABA due to hydroxylation by

ABA 8'-hydroxylase[2] or glucose conjugation[3] and degradation of OST1 by a recently discovered protein HIGH OSMOTIC STRESS GENE EXPRESSION 15 (HOS15)[4]. We envision that, combining microfiber electronics with specific agonists/antagonists and molecular/genetic approaches, the cascaded signalling pathways underlying stomatal control can be precisely deciphered.

As shown in Figure 2c, a significant delay of light-induced stomatal opening was also observed because of the long signaling cascade (Figure S10b) [5-7]. Specifically, blue light causes auto-phosphorylation of phototropins (PHOT1 and PHOT2), which leads to phosphorylation of the protein kinase BLUE LIGHT SIGNALING 1 (BLUS1) and its binding with a protein kinase - blue light-dependent H^+-ATPase phosphorylation (BHP). The complex then activates phosphatase 1 (PP1) and its regulatory subunit PRSL1, which causes phosphorylation of H^+-ATPase. Consequently, protons are transported outwards, K^+ ions and water flow in, cell turgor increases, and finally stomata open. In addition, the long delay of light-induced stomatal opening can be attributed to ABA accumulation in cytosol during the dark period before light exposure[2]. After termination of light exposure, photosynthesis stops, and consequently ABA accumulates thereby leading to stomatal closure[8]. The long delay of stomatal re-closure after turning off the light as observed in Figure 2c is likely because of the time needed for sufficient accumulation of ABA. Some researchers also pointed out the important role of red light for stomatal regulation, but the underlying mechanism is still unclear[9]. Combing with chemical, molecular, and genetic tools, our microfiber electronics shall allow scrutinization of the still not fully understood signaling pathways and their kinetics.

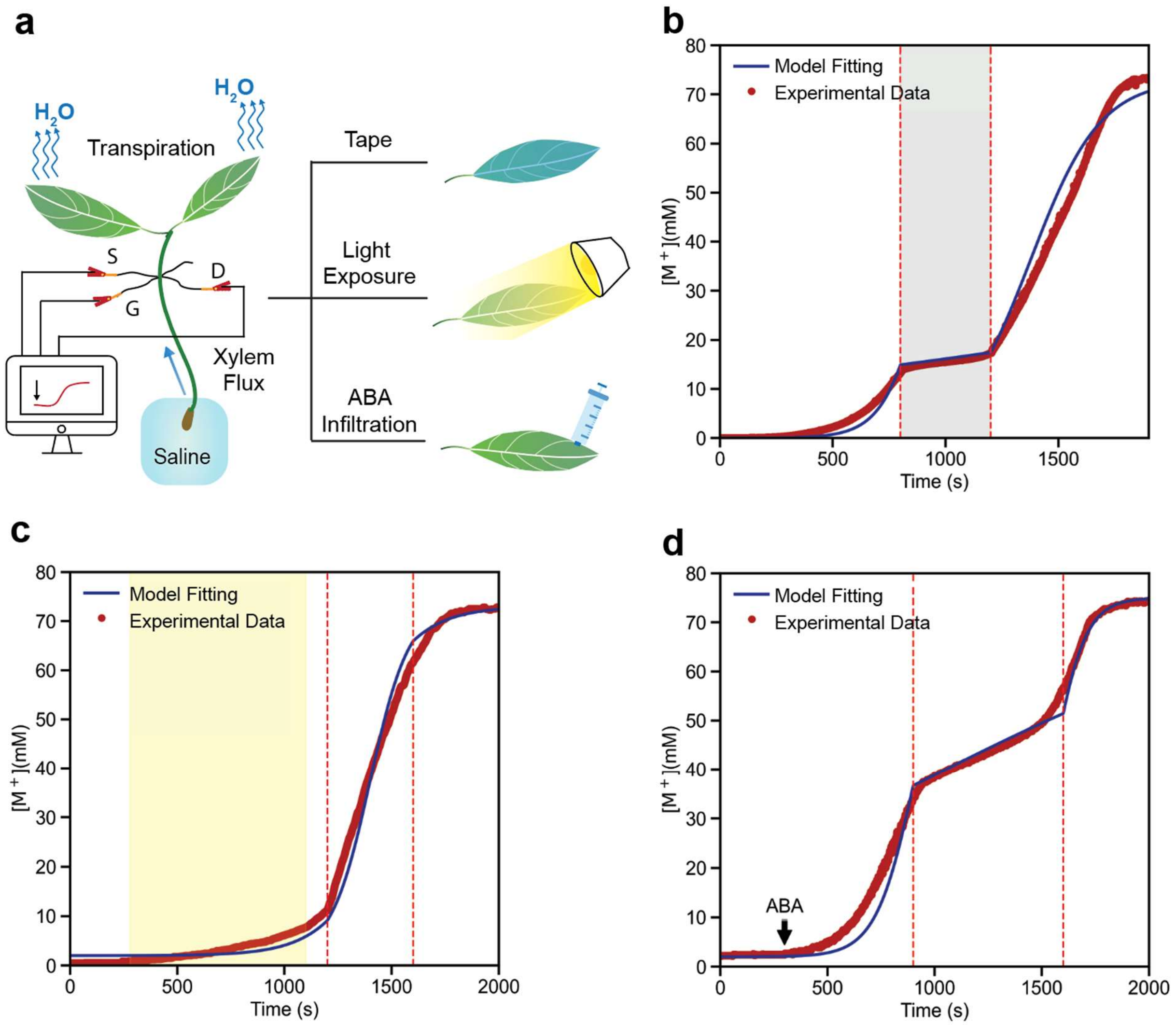

Figure S11. Fitting the experimental data in Figure 2c using our 1D convection model for xylem transport.

Supplementary discussion 4: Applying 1D convection xylem flow model to different transpiration conditions

As seen from Figure S11, our model fits well with the experimental data and can derive the xylem flow rates under various transpiration conditions (i.e., flow rate was deaccelerated by ABA hormone: from 0.094 cm/s to 0.01 cm/s; nearly halted upon total blockage of the stoma by tape: 0.092 cm/s to 0.005 cm/s; accelerated upon light exposure:

0.056 cm/s to 0.11 cm/s). This indicates generalizability of the model and reliability of the measurements. Consistently, a previous study on the same plant species (devil's ivy) also reported similar xylem flow rate, specifically, 0.08 cm/s to 0.4 cm/s, depending on plant size and cultivation conditions[10]. This further corroborates the reliability of our measurement and model. As seen from Figure S11b-c, the turning points of xylem flow rate can be clearly identified as indicated by the dashed lines. Physical blocking of stoma caused immediate change, whereas the change of xylem flow rate was much delayed in response to ABA or light exposure. For the sake of simplicity, we assumed a sudden change in flow rate at the turning points. This assumption led to small discrepancy with the experimental measurements at the turning points.

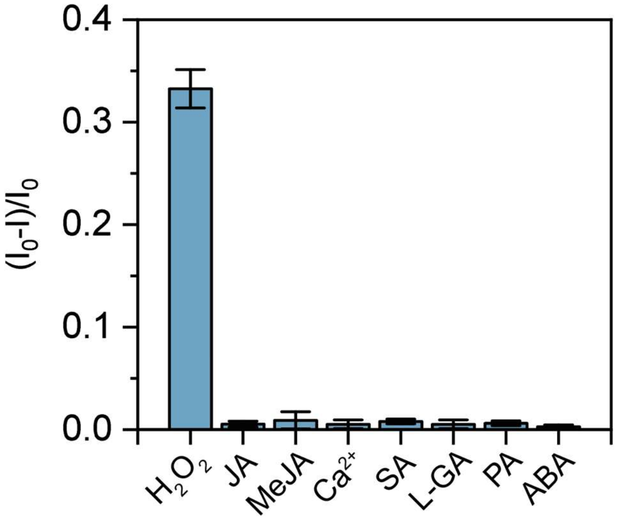

Figure S12. Selectivity of fOECT-H_2O_2 in the presence of possible interferants at their typical concentrations when the plant is stressed, e.g., by wounding (1 mM for Ca^{2+}, 100 μM for others). JA: jasmonic acid; MeJA: methyl jasmonate; SA: salicylic acid; L-GA: L-glutamic acid; PA: phenylacetic acid; ABA: abscisic acid. Data is shown as mean ± s.d. from three independent experiments.

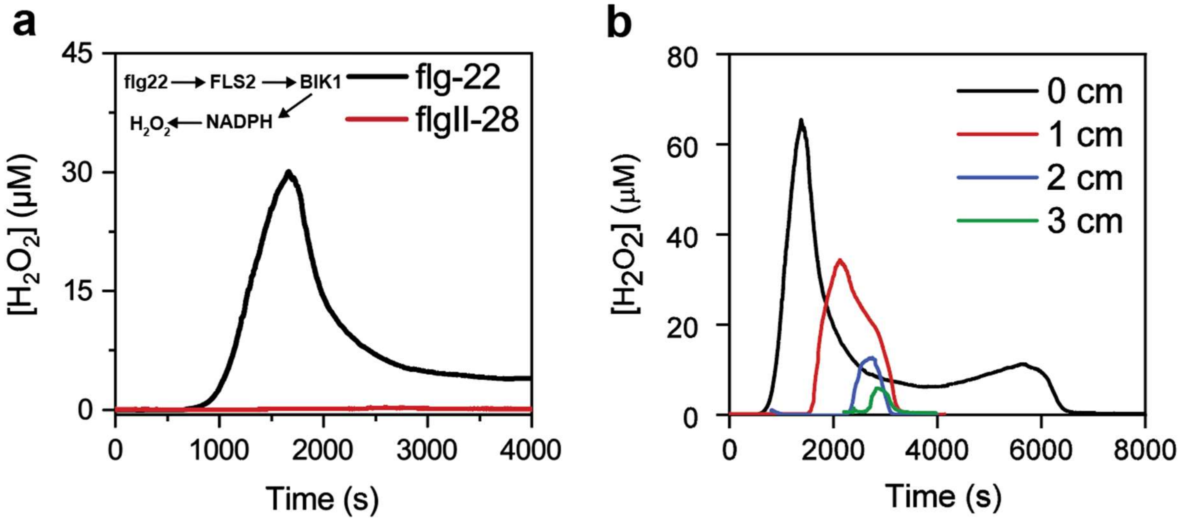

Figure S13. a, Flg22 induced H_2O_2 signal. Due to absence of the specific receptor, flgII-28, another bacterial flagellar filament, cannot induce H_2O_2 signal in plant. flgII-28 and flg-22 were injected into the plant stem 1 cm away from fOECT-H_2O_2. at the same position successively (30 min interval). Inset shows the signaling cascade for flg22 inducing generation of H_2O_2. **b,** flg22 induced H_2O_2 wave recorded at different distances (in the direction towards the leaves).

Supplementary discussion 5: Flg22 and heat induce H_2O_2 signal in plant

As compared to wounding and mild-heating triggered responses (Figure 3e), the kinetics of flg22 induced H_2O_2 wave is slower (longer delay, less steep take-off). It suggests that the underlying signaling kinetic is slow. It involves activation of the plasma membrane-localized receptor FLAGELLIN SENSITIVE2 (FLS2) and its disassociation with BOTRYTIS-INDUCED KINASE1 (BIK1)[11]. BIK1 then phosphorylates NADPH oxidase (NOX), leading to production of O^{2-} and its dismutation to form H_2O_2 by superoxide dismutase (SOD)[12].

As expected, the H_2O_2 wave decay in both amplitude and kinetics over the distance (Figure S13b). It is interesting to note that H_2O_2 concentration recorded at the wounding site rose again ~1 hour after the peak. Similar phenomenon has also been observed

previously[13]. Conceivably, upon binding with flg22, FLS2 is first endocytosed and then recycled back to cell membrane to be activated by flg22 again[14]. Our experiment reveals the kinetics of such recycling. It is also noted, compared to wounding induced wave (Figure 5a, b), the rise time (>532 s) and propagation velocity (<10 μm s^{-1}) of flg22 induced H_2O_2 wave is much slower, presumably at least in part due to the endocytosis and slow recycling of FLS2.

The mechanism of heat stress-induced H_2O_2 generation and propagation is not yet fully understood. It has been suggested that heat disrupts the organization of unsaturated fatty acid chains in membrane lipids, leading to a more fluidic and less stable membrane which, in turn, permits influx of calcium ions[15]. In addition, heat-induced conformational change of Ca^{2+} channels may also cause Ca^{2+} influx[16]. As discussed in the main text, increase of cytosol Ca^{2+} stimulates the generation of H_2O_2 wave. Unsurprisingly, the response of heat-induced H_2O_2 wave is faster than flg22-induced wave because Ca^{2+} influx is quickly caused by physical effect.

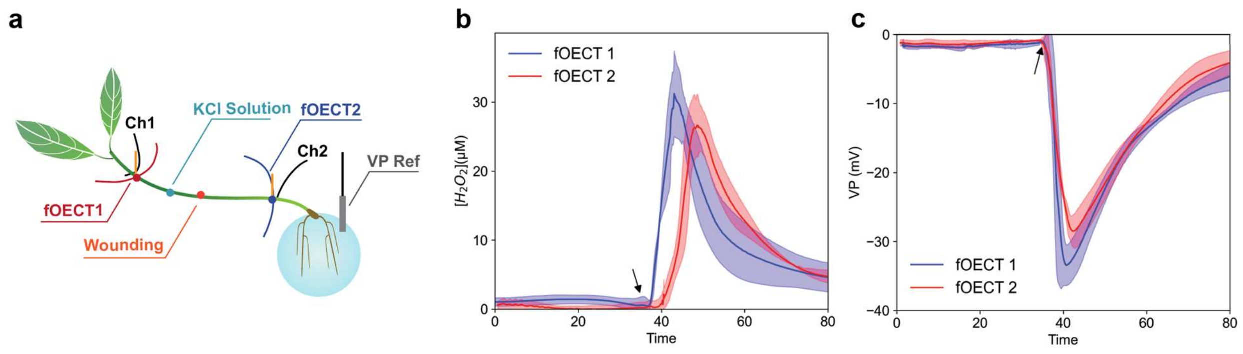

Figure S14. Infusion of KCl solution (5 mM) doesn't prevent propagation of H_2O_2 and VP waves to fOECT1. Each data point is shown as mean ± standard deviations. 3 different plants were used; both H_2O_2 and VP waves were simultaneously recorded on each plant.

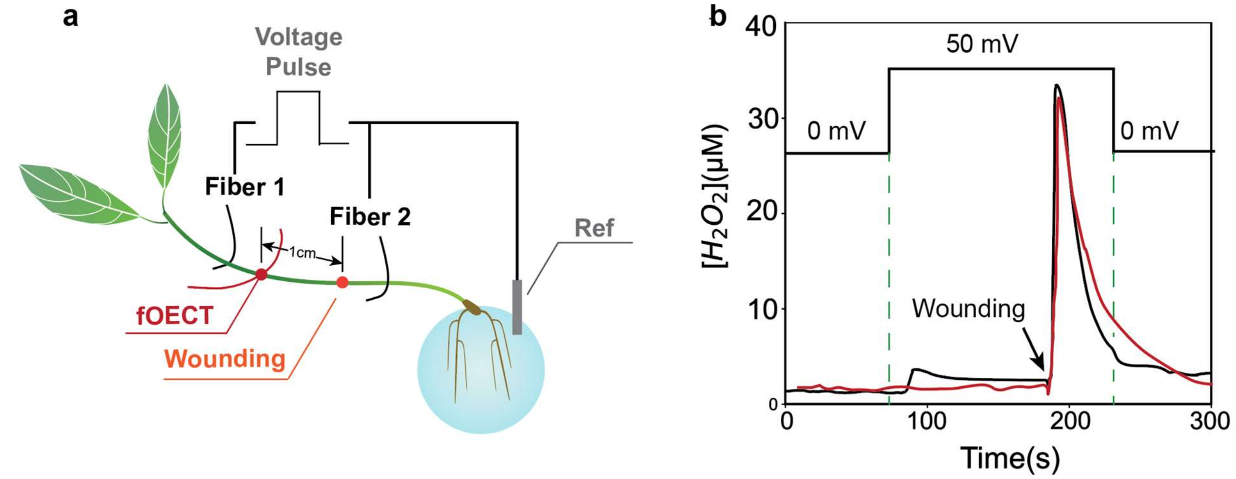

Figure S15. Voltage Influence to H_2O2-fOECT. a, Illustration of the experimental setup. A voltage pulse was applied via two conductive fibers. **b,** Wounding-induced H_2O_2 waves without (red) and with (black) application of the voltage pulse on the same plant.

Supplementary discussion 6: Voltage-induced H_2O_2 response

The possible interference of VP to H_2O_2-fOECT was investigated by applying a voltage pulse with an amplitude similar to that of VP (50 mV). The voltage pulse caused a small and lasting response of H_2O_2-fOECT (Figure S5b). The long delay (>10 s) of this response rules out the possibility of electrical interference to fOECT. Therefore, the response indicates an actual small increase of H_2O_2 (<3 μM). Such voltage-induced H_2O_2 is a new phenomenon requiring further investigation. Note that it is distinct to the rapidly-evoked large H_2O_2 wave triggered by wounding (>30 μM, <4 s delay). And as shown in Figure S5b, the presence of the voltage pulse didn't appreciably affect the amplitude or kinetics of wound-induced H_2O_2 wave. Therefore, VP, which accompanies H_2O_2 wave, doesn't electrically interfere the recording by H_2O_2-fOECT.

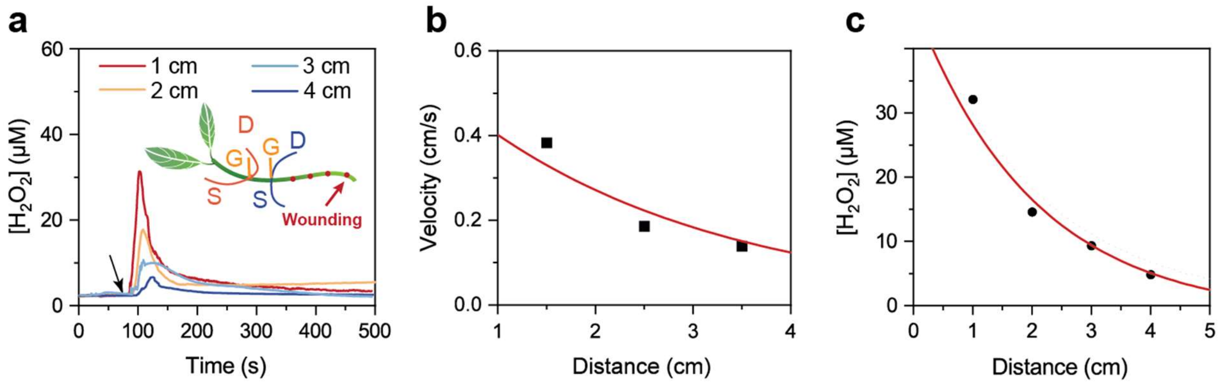

Figure S16. Distance decay of H_2O_2 wave. a, Wound was inflicted 1/2/3/4 cm away from 2 fOECTs (1 cm apart). **b,** Velocity of H_2O_2 wave decays with a constant of 2.55 cm obtained from the exponential fitting (red curve). **c**, Amplitude of H_2O_2 wave decays with a constant of 1.53 cm obtained from the exponential fitting (red curve).

Reference

1. Hsu, P.K., Dubeaux, G., Takahashi, Y., and Schroeder, J.I. (2021). Signaling mechanisms in abscisic acid-mediated stomatal closure. Plant J 105(2):307-321. https://doi.org/10.1111/tpj.15067.
2. Movahedi, M., Zoulias, N., Casson, S.A., et al. (2021). Stomatal responses to carbon dioxide and light require abscisic acid catabolism in Arabidopsis. Interface Focus 11(2):20200036. https://doi.org/10.1098/rsfs.2020.0036.
3. Munemasa, S., Hauser, F., Park, J., et al. (2015). Mechanisms of abscisic acid-mediated control of stomatal aperture. Curr Opin Plant Biol 28:154-162. https://doi.org/10.1016/j.pbi.2015.10.010.
4. Ali, A., Pardo, J.M., and Yun, D.J. (2020). Desensitization of ABA-Signaling: The Swing From Activation to Degradation. Front Plant Sci 11:379. https://doi.org/10.3389/fpls.2020.00379.
5. Yang, J., Li, C., Kong, D., et al. (2020). Light-Mediated Signaling and Metabolic Changes Coordinate Stomatal Opening and Closure. Front Plant Sci 11:601478. https://doi.org/10.3389/fpls.2020.601478.
6. Roelfsema, M.R.G., and Hedrich, R. (2005). In the light of stomatal opening: new insights into 'the Watergate'. New Phytologist 167(3):665-691.
7. Hiyama, A., Takemiya, A., Munemasa, S., et al. (2017). Blue light and CO(2) signals converge to regulate light-induced stomatal opening. Nat Commun 8(1):1284. https://doi.org/10.1038/s41467-017-01237-5.
8. Neill, S., Barros, R., Bright, J., et al. (2008). Nitric oxide, stomatal closure, and abiotic stress. J Exp Bot 59(2):165-176. https://doi.org/10.1093/jxb/erm293.
9. Matthews, J.S.A., Vialet-Chabrand, S., and Lawson, T. (2020). Role of blue and red light in stomatal dynamic behaviour. J Exp Bot 71(7):2253-2269. https://doi.org/10.1093/jxb/erz563.
10. Wistuba, N., Reich, R., Wagner, H.-J., et al. (2000). Xylem flow and its driving forces in a tropical liana: concomitant flow-sensitive NMR imaging and pressure probe measurements. Plant Biology 2(06):579-582. https://doi.org/10.1055/s-2000-16644.
11. Li, L., Li, M., Yu, L., et al. (2014). The FLS2-associated kinase BIK1 directly phosphorylates the NADPH oxidase RbohD to control plant immunity. Cell Host Microbe 15(3):329-338. https://doi.org/10.1016/j.chom.2014.02.009.
12. Kadota, Y., Sklenar, J., Derbyshire, P., et al. (2014). Direct regulation of the NADPH oxidase RBOHD by the PRR-associated kinase BIK1 during plant immunity. Mol Cell 54(1):43-55. https://doi.org/10.1016/j.molcel.2014.02.021.
13. Ngou, B.P.M., Ahn, H.K., Ding, P., and Jones, J.D.G. (2021). Mutual potentiation of plant immunity by cell-surface and intracellular receptors. Nature 592(7852):110-115. https://doi.org/10.1038/s41586-021-03315-7.
14. Robatzek, S., Chinchilla, D., and Boller, T. (2006). Ligand-induced endocytosis of the pattern recognition receptor FLS2 in Arabidopsis. Genes Dev 20(5):537-542. https://doi.org/10.1101/gad.366506.
15. Saidi, Y., Finka, A., Muriset, M., et al. (2009). The heat shock response in moss plants is regulated by specific calcium-permeable channels in the plasma membrane. The Plant Cell 21(9):2829-2843. https://doi.org/10.1105/tpc.108.065318.

16. Mittler, R., Finka, A., and Goloubinoff, P. (2012). How do plants feel the heat? Trends in biochemical sciences 37(3):118-125. https://doi.org/10.1016/j.tibs.2011.11.007.
