# Supplementary material for: Mutually reinforcing and transpiration-dependent propagation of H2O2 and variation potential in plants revealed by fiber organic electrochemical transistors
Source: Innovation (Camb). 2025 Jan 6;6(5):100800. doi: 10.1016/j.xinn.2025.100800 (PMC12105492; doi:10.1016/j.xinn.2025.100800)
Supplement: Document S2. Article plus supplemental information [file mmc2.pdf]

REPORT

OPEN ACCESS

Mutually reinforcing and transpiration-dependent propagation of H_2O_2 and variation potential in plants revealed by fiber organic electrochemical transistors

Hanqi Wen,[1,2] Lingxuan Kong,[1] Xinlu Zhu,[3] Yansong Miao,[3,4] Xing Sheng,[2,5] Xiaodong Chen,[2,4,6] Yuxin Liu,[7,8,9,*] and Peng Chen[1,2,4,*]
*Correspondence: lyx@nus.edu.sg (Y.L.); chenpeng@ntu.edu.sg (P.C.)

Received: July 26, 2024; Accepted: January 5, 2025; Published Online: January 6, 2025; https://doi.org/10.1016/j.xinn.2025.100800

© 2025 The Authors. Published by Elsevier Inc. on behalf of Youth Innovation Co., Ltd. This is an open access article under the CC BY license (http://creativecommons.org/licenses/by/4.0/).

GRAPHICAL ABSTRACT

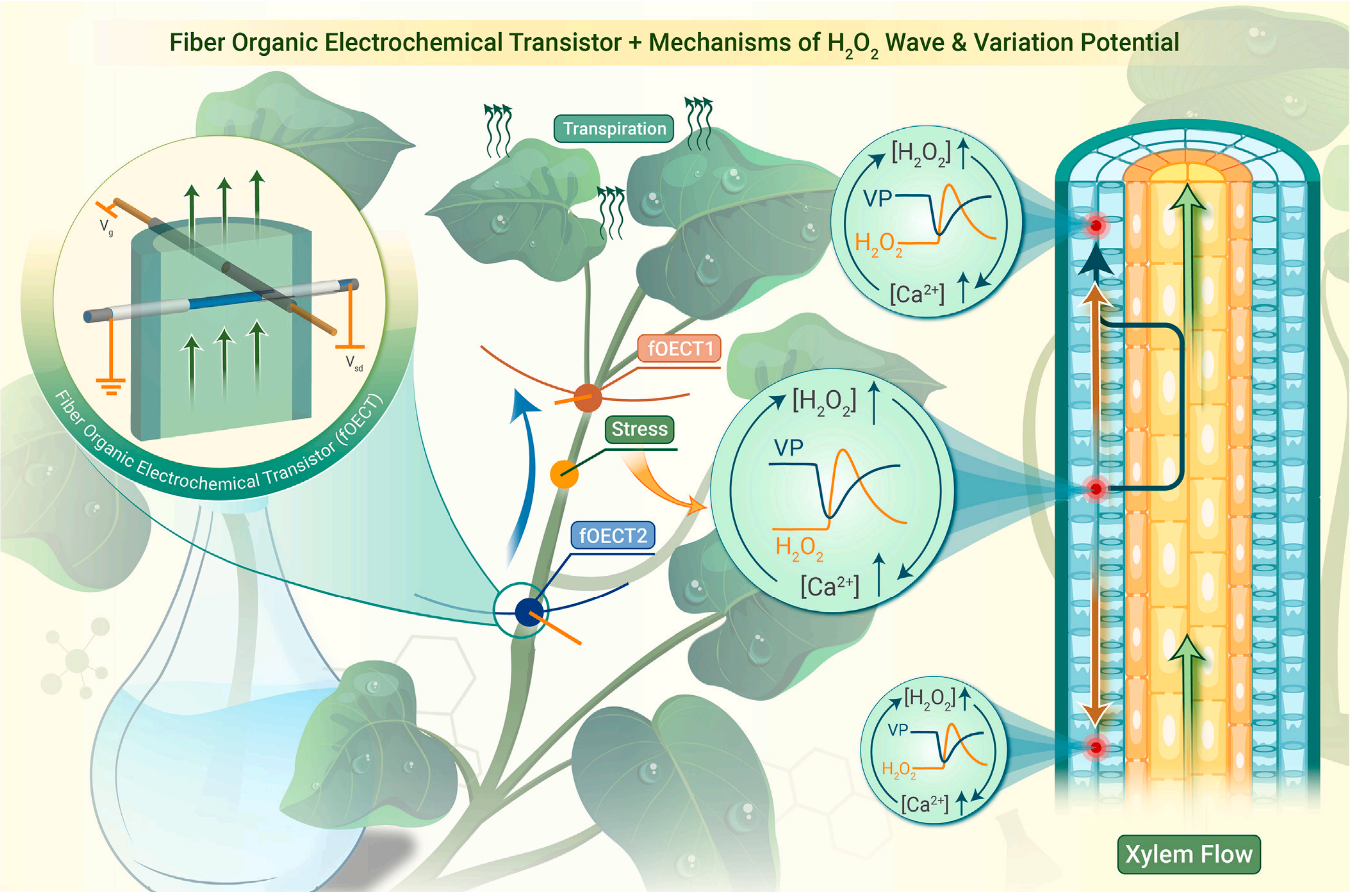

PUBLIC SUMMARY

- Plants use hydrogen peroxide (H_2O_2), variation potential (VP), and xylem flow for cell signaling and adaptation.
- Microfiber-shaped organic electrochemical transistors (fOECTs) reveal interplays among VP, H_2O_2, and xylem flow.
- A transpiration- and intensity-dependent H_2O_2-VP mutual-reinforcing propagation mechanism is proposed.
- Microfiber electronics enable the *in situ* study of plant physiological processes with high temporospatial resolution.

Mutually reinforcing and transpiration-dependent propagation of H_2O_2 and variation potential in plants revealed by fiber organic electrochemical transistors

Hanqi Wen,[1,2] Lingxuan Kong,[1] Xinlu Zhu,[3] Yansong Miao,[3,4] Xing Sheng,[2,5] Xiaodong Chen,[2,4,6] Yuxin Liu,[7,8,9,*] and Peng Chen[1,2,4,*]
[1]School of Chemistry, Chemical Engineering and Biotechnology, Nanyang Technological University, Singapore 637457, Singapore
[2]Institute of Flexible Electronics Technology of THU, Jiaxing 314000, China
[3]School of Biological Sciences, Nanyang Technological University, Singapore 637551, Singapore
[4]Institute for Digital Molecular Analytics and Science (IDMxS), Nanyang Technological University, Singapore 636921, Singapore
[5]Department of Electronic Engineering, Beijing National Research Center for Information Science and Technology, Laboratory of Flexible Electronics Technology, Tsinghua University, Beijing 100084, China
[6]Innovative Center for Flexible Devices (iFLEX), Max Planck-NTU Joint Laboratory for Artificial Senses, School of Materials Science and Engineering (MSE), Nanyang Technological University, Singapore 639798, Singapore
[7]Department of Biomedical Engineering (BME), National University of Singapore, Singapore 117583, Singapore
[8]The N.1 Institute for Health, National University of Singapore, Singapore 117456, Singapore
[9]Institute for Health Innovation and Technology (iHealthtech), National University of Singapore, Singapore 117599, Singapore
*Correspondence: lyx@nus.edu.sg (Y.L.); chenpeng@ntu.edu.sg (P.C.)
Received: July 26, 2024; Accepted: January 5, 2025; Published Online: January 6, 2025; https://doi.org/10.1016/j.xinn.2025.100800
© 2025 The Authors. Published by Elsevier Inc. on behalf of Youth Innovation Co., Ltd. This is an open access article under the CC BY license (http://creativecommons.org/licenses/by/4.0/).
Citation: Wen H., Kong L., Zhu X., et al., (2025). Mutually reinforcing and transpiration-dependent propagation of H_2O_2 and variation potential in plants revealed by fiber organic electrochemical transistors. The Innovation **6(5)**, 100800.

Plants use hydrogen peroxide (H_2O_2) and variation potential (VP) waves as well as chemical transport by transpiration-driven xylem flow to facilitate cell signaling, cell-to-cell communication, and adaptation to environmental stresses. The underlying mechanisms and complex interplay among H_2O_2, VP, and transpiration are not clearly understood because of the lack of bioengineering tools for continuous *in planta* monitoring of the dynamic biological processes. Here, we tackle the challenge by developing microfiber-shaped organic electrochemical transistors (fOECTs) that can be threaded into the plants. The sensorized microfiber revealed that both H_2O_2 and VP waves propagate faster toward the leaves than toward the roots because of the directional long-distance transport of H_2O_2 in the xylem. In addition, the revealed interplays among VP, H_2O_2, and xylem flow strongly suggest a transpiration- and intensity-dependent H_2O_2-VP mutual-reinforcing propagation mechanism. The microfiber electronics offer a versatile platform for the *in situ* study of dynamic physiological processes in plants with high temporospatial resolution.

INTRODUCTION

Initially only considered as the inevitable toxic product of metabolic and enzymatic activities, H_2O_2, the major reactive oxygen species (ROS) in plants, has been increasingly recognized as the crucial signaling molecule playing roles in various plant physiological processes and responses to biotic or abiotic stresses (e.g., wounding), thanks to its long lifespan.[1,2] Some studies[3–5] have suggested that the propagation of the H_2O_2 signal is coupled with Ca^{2+} signaling and propagating variation potential (VP) to produce coordinated responses to stresses through some not-well-understood mechanisms. We further reason that transpiration-driven xylem flow may play a key role in long-distance H_2O_2 signaling for cell-to-cell communication. The mechanisms of these dynamic processes and the coupling between the H_2O_2 wave, VP, and transpiration are still under debate, largely due to the lack of probing tools with high sensitivity, sufficient temporal and spatial resolution, minimal invasiveness, and compatibility with plant tissues.

Currently, H_2O_2 is monitored using fluorescence probes,[6–8] but they are not able to report the H_2O_2 dynamics because their interaction with H_2O_2 is irreversible or slow, and long-term monitoring is not possible due to photobleaching.[9] Additionally, it is difficult to calibrate the exact H_2O_2 concentration because the fluorescence signal can be significantly interfered with by the physiological conditions in plant tissues, such as pH.[10] In the recent seminal study by Strano et al., the wound-induced H_2O_2 wave on the leaves of various plant species was spatiotemporally monitored for the first time using H_2O_2-selective fluorescent single-walled carbon nanotubes.[11] But still, it shares some problems with other fluorescence imaging methods. First, the measurement is compromised by the undefined diffusion and distribution of the fluorescence probe. Secondly, image acquisition is too slow to resolve the fast signaling events. Thirdly, deep tissue monitoring is prevented by the limited light penetration depth.

The xylem flow rate is often determined by the heat dissipation method (HDM) or isotope tracing technique.[12] The former, which involves the insertion of large probes and heating, is highly invasive, inherently only applicable to large woody plants, and of low temporal resolution. The latter requires a special experimental facility and condition. Existing solid-state electrodes for VP recording exhibit high and unstable impedance at the electrode-fluid interface and substantial mechanical mismatch with soft plant tissue, which cause adverse biological reactions, high noise, and motion artifacts.[13]

To address the abovementioned research gaps and technical challenges, we report microfiber-shaped organic electrochemical transistors (fOECTs). The conductive polymer-based devices allow long-term and continuous *in planta* recording with a stable bioelectronic interface.[14] Leveraging its dual electronic-ionic conductivity and excellent biological mechanical compatibility,[15–17] microfiber electronics was used to record the transpiration-driven xylem flow, H_2O_2 wave, and VP with sub-second temporal resolution. The generalizable microfiber electronics platform enables the observation of temporospatial dynamics and interplay between these processes and further leads to a proposed mechanism of transpiration- and intensity-dependent H_2O_2-VP mutual-reinforcing propagation.

MATERIALS AND METHODS

Preparation and characterization of M^+-fOECT and H_2O_2-fOECT

Poly(3,4-ethylenedioxythiophene) polystyrene sulfonate (PEDOT:PSS) solution (PH1000), dimethyl sulfoxide (DMSO), ethylene glycol (EG), 4-dodecylbenzenesulfonic acid (DBSA), 5% Nafion solution, chloroplatinic acid hexahydrate ($H_2PtCl_6 \cdot 6H_2O$), diphenyleneiodonium chloride (DPI), $GdCl_3$, and 3% w/w H_2O_2 solution were purchased from Sigma-Aldrich. 0.2% w/w DBSA, 5% w/w EG, and 5% w/w DMSO were added into the PEDOT:PSS solution with stirring, followed by sonication for 30 min and filtering by a 0.45 μm nylon syringe filter to remove aggregates. Silk fiber was cleaned by sonication in deionized (DI) water, ethanol, and acetone successively and then dried in a 70°C oven. The fiber was then dip coated in the prepared PEDOT:PSS solution and drawn by a tensile meter at a speed of 2 cm s^{-1} to maintain a constant shear rate of 50 s^{-1}, thereby forming a uniform conductive layer on the surface. The coated fiber was dried in an oven at 75°C for 30 min and then further heated at 125°C for 30 min. Silver paste (Dycotech) was applied to both ends of PEDOT:PSS fibers except for the middle (5 mm), which acted as the sensing region. Except for the two contact ends, a layer of Ecoflex (BASF) was coated on the silver paste as an insulating layer.

Two of the obtained conductive microfibers constitute an M^+-fOECT. The H_2O_2-fOECT consists of a conductive microfiber as the source-drain channel and a Pt nanoparticle (PtNP)-coated Au wire as the gate electrode. An Au wire (0.25 mm) was successively cleaned by DI water, isopropyl alcohol, and acetone. After drying in a 70°C oven, it was further cleaned by 10 cycles of cyclic voltammetry (CV) scanning in 0.5 M H_2SO_4 from 0 to 1 V with a scan rate of 100 mV/s. PtNPs were electrochemically deposited on gold wire by CV sweep from 0 to 1.1 V (with a saturated calomel electrode as reference) in an electrolyte containing 0.5 M H_2SO_4 and 1 mM H_2PtCl_6. After being rinsed by DI water, the

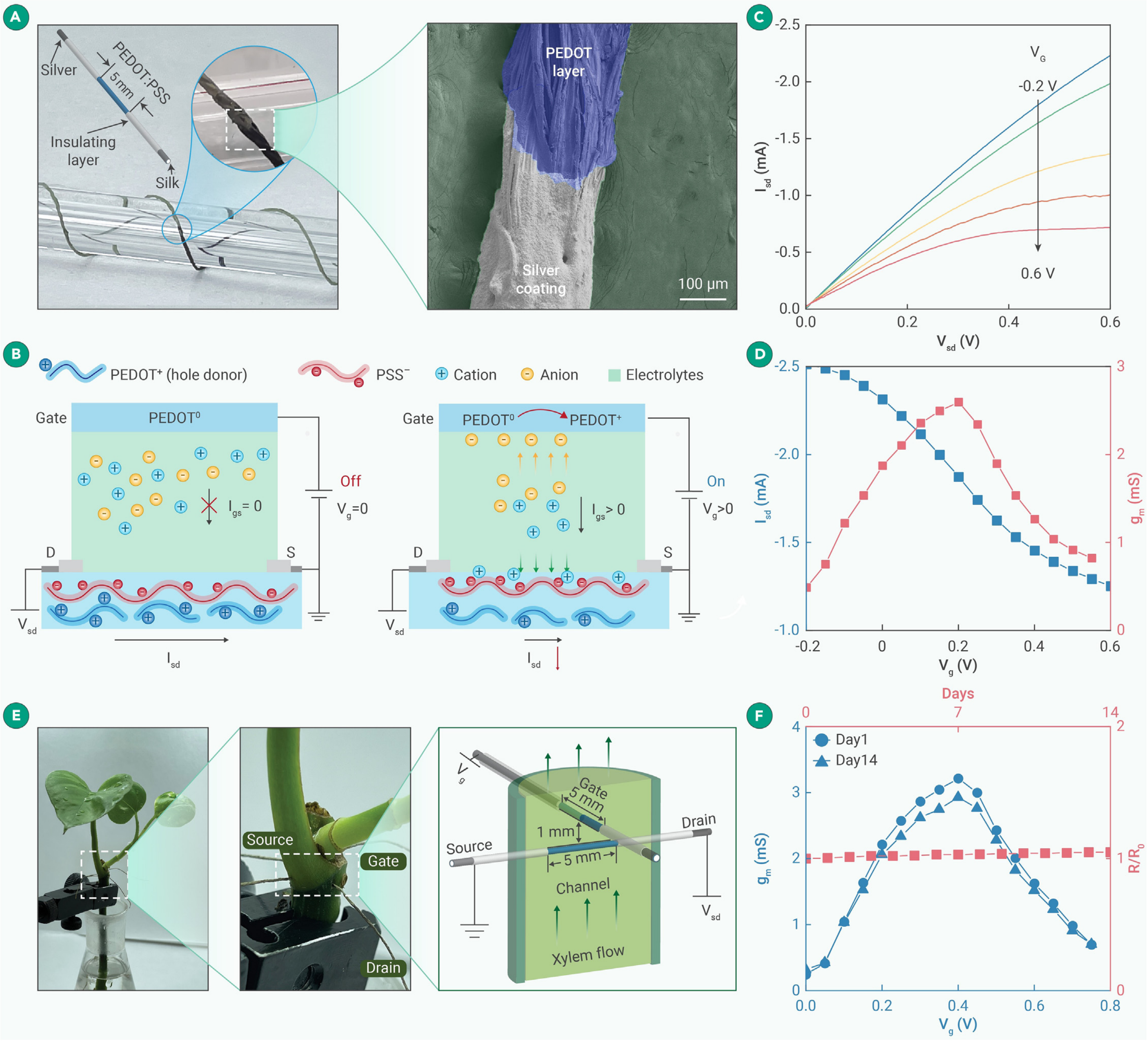

Figure 1. Characterizations of the M$^+$-fOECT (A) Schematic, optical (left), and scanning electron microscopy (SEM; right; pseudo-coloring applied to PEDOT:PSS layer) images of a microfiber. (B) Cation-responsive mechanism of M$^+$-fOECT. (C) Output curve of M$^+$-fOECT in agarose gel containing 75 mM KCl solution. (D) Transfer (black) and transconductance (red) curves of M$^+$-fOECT in agarose gel containing 75 mM KCl solution. (E) fOECT threaded in a devil's ivy. (F) Transconductance curves of M$^+$-fOECT (black) immediately and 14 days after implantation and resistance variation (red) over the course.

gate electrode was immersed in Nafion (5% in ethanal) for 30 min and naturally dried in a fume hood. All fOECT experiments were conducted on a semiconductor analyzer (Keithley 4200 SCS). To obtain the output curve, V_{sd} was swept from 0 to -1 V under various gate voltages (V_g). For the transfer and transconductance (g_m) curves, V_g was swept from -0.2 to 0.6 V with a step size of 0.05 V and V_{sd} at -0.2 V.

Plant material, growth condition, and *in situ* measurements

Devil's ivy (*Epipremnum aureum*) was collected from a campus nursery and grown for 1 week at 21°C and 80% relative humidity under a 14-h-light/10-h-dark photoperiod, 50 μmol s^{-1} m^{-2} light intensity. The plants for experiments have only branches with 2–5 leaves (total surface area of ~100 cm^2). To have convenient control of the cultivation and experimental conditions, the plants were kept hydroponically. Microfibers were threaded through the plant stem using a sewing needle. Wounding was inflicted using a needle (diameter of 0.45 mm unless stated otherwise). To induce heat stress, 50°C water was continuously dropped onto the plant stem for 30 s. The temperature of the heated region was determined to be 45°C by an infrared (IR) camera. VP was recorded by a differential amplifier (BrainVision). For the light stimulation experiment, before the plant was irradiated by a solar light simulator (94023A, Newport), it was kept in a dark room for 12 h. Agents were infused into the plant stem through a previously created small hole (inflicted 30 min earlier). The take-off point is when the signal amplitude reaches 5× of the baseline fluctuation. The rise time is defined as the time gap between the take-off point and when the signal reaches 63.2% of the peak. By measuring the distance and the take-off time-point differences of two sensors, the velocity of signal propagation is calculated.

RESULTS AND DISCUSSION

fOECT device designed for continuous *in planta* monitoring

Conventional OECT devices are fabricated on planar substrates (e.g., silicon).[18] To minimize tissue invasiveness, one-dimensional (1D) microfiber-shaped OECTs can be developed.[19–21] Herein, degummed silk fiber was used as the substrate material due to its small diameter (~240 μm), hydrophilicity, excellent

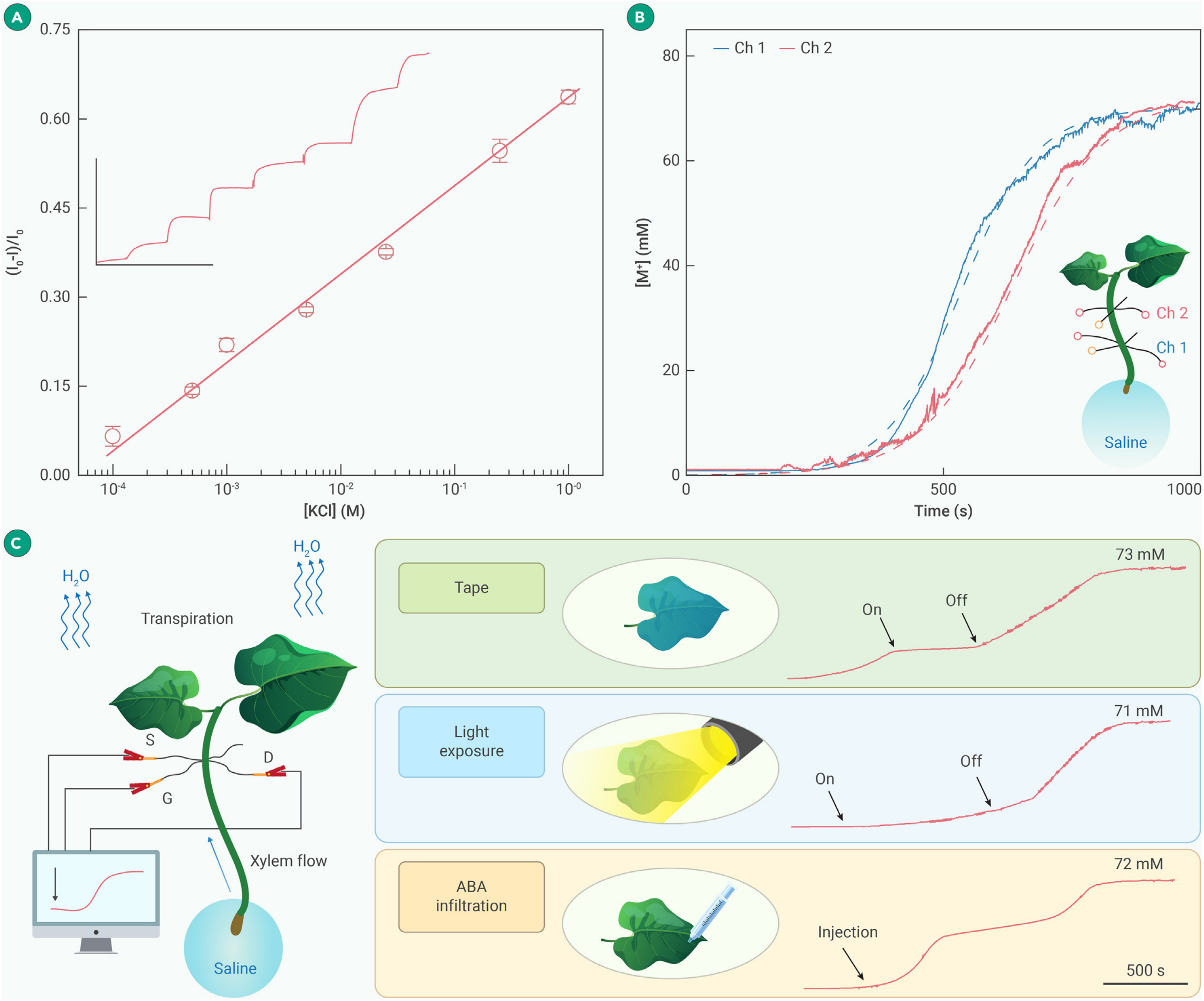

Figure 2. Monitoring cation variation, xylem transport, and transpiration by the M^+-fOECT (A) Calibration curve of percentage change of I_{sd} vs. variation of K^+ concentration obtained from 4 different fOECTs (error bars represent standard deviations). Inset shows the I_{sd} percentage change of a fOCET-M^+ responding to the drop-wise addition of KCl solution to various concentrations (scale bar: vertical = 0.3 mA, horizontal = 500 s). (B) Cation concentration increase due to xylem flow transport after immersing the rootless plant in 75 mM KCl solution, monitored by 2 fOECTs implanted 1 and 2 cm above the solution. Dashed curves are the fittings by the 1D convection model. (C) Cation concentration variation due to transpiration-driven xylem flow measured at 2 cm above the solution responding to covering stomata using tape, exposure to simulated sunlight (200 μmol m^{-2} s^{-1}), and infiltration of abscisic acid (ABA; 10 μM, 1 mL).

biocompatibility, low Young's modulus, good mechanical strength, and high flexibility (Figure 1A).[22] A thin layer of PEDOT:PSS (~5 μm) was dip coated on the fiber to form a conducting channel. Assisted by surfactant DBSA, PEDOT:PSS chains electrostatically interact with the hydrophilic silk fiber, allowing uniform coating along the fiber (as evidenced in Figure S1), and polar molecules (DMSO and EG) were used to further improve the conductivity by weakening the electrostatic interaction between PEDOT and PSS, consequently straightening coiled PEDOT chains to give a lower energy barrier for inter-grain charge hopping. After the silver paste was coated on both ends as the source and drain, the fiber was insulated with Ecoflex polymer except for the middle sensing region (Figure 1A).

A cation-responsive fOECT (M^+-fOECT) was constructed by two PEDOT:PSS microfibers. When the gate fiber is biased by a positive voltage (V_g), it is oxidized, while in the channel fiber, PEDOT is reduced and thus dedoped (Figure 1B).[23] Cations in the electrolyte (M^+) are driven in to neutralize the decoupled polyanion (PSS^-):

$$\text{gate: } PEDOT^0 - e^- = PEDOT^+ \text{ and}$$

$$\text{channel: } PEDOT^+PSS^- + e^- + M^+ = PEDOT^0 + PSS^-M^+.$$

Consequently, the decreased hole density on the channel fiber gives a lower source-drain current (I_{sd}) in a cation-concentration-dependent manner. Owing to the reversible doping/dedoping process,[24] the M^+-fOECT exhibits good cycling stability with <10% current variation over 3,000 cycles (Figure S2), and its small response time constant (0.11 s) ensures the capability to resolve fast dynamic biological processes (Figure S3). Furthermore, the M^+-fOECT is not responsive to H_2O_2 (Figure S4A).

The performance of the M^+-fOECT was first evaluated in an artificial tissue model, i.e., agarose gel (mimicking plant tissue and interstitial fluid) covered with a water-impermeable parafilm (mimicking plant cuticle). With a small V_{sd} (−0.2 V) being applied, the V_g negatively modulates I_{sd} in a depletion mode (Figure 1C). g_m, which is the partial derivative between I_{sd} and V_g, characterizes the sensitivity of an OECT. As shown by the transfer and g_m curves (Figure 1D), the

www.the-innovation.org

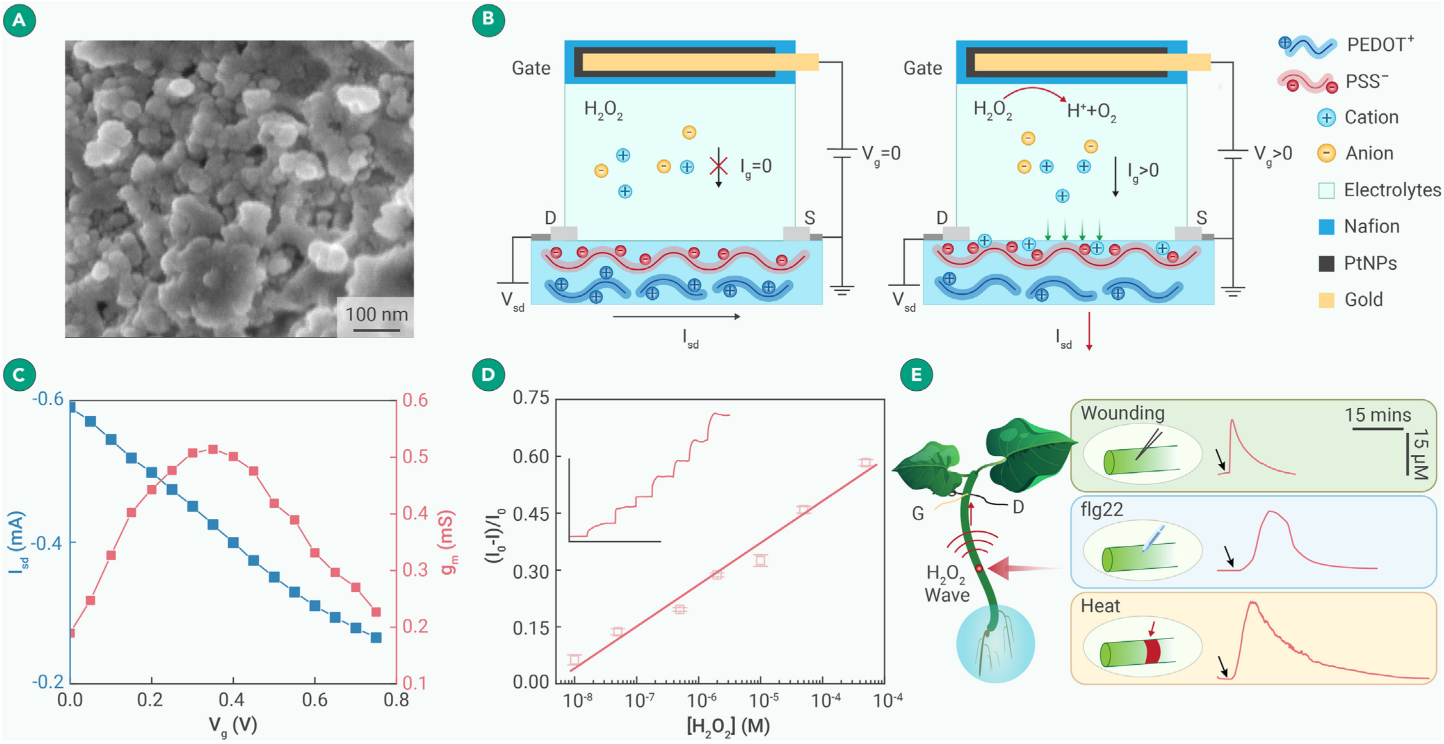

Figure 3. H_2O_2-fOECT for continuous *in planta* monitoring (A) SEM image of Pt nanoparticles coated on an Au microfiber. (B) Working mechanism of H_2O_2-fOECT. (C) Transconductance curve (red) and transfer curve (black) of H_2O_2-fOECT in 10 μM H_2O_2 solution. (D) Calibration curve of percentage change of I_{sd} vs. H_2O_2 concentration obtained from 4 different fOECTs (error bars represent standard deviations). Inset shows the responses of a fOECT upon the drop-wise addition of H_2O_2 solution to reach various defined concentrations (scale bars: vertical = 0.3 mA, horizontal = 1,000 s). (E) Wounding-, heat-, and flg22-induced H_2O_2 waves. All stimulations were applied 1 cm away from the fOECT at the time points indicated by the arrows.

highest g_m (2.6 mS) was achieved at V_g = ~0.2 V, which is thus used for all cation recording experiments. The device-to-device variation in g_m is small, indicating the high fabrication consistency (Figure S5A), and the g_m change of the M^+-fOECT was <10% even at a high bending angle of 135° (Figure S6), indicating its accommodation to tissue bending. The long-term implantation of the M^+-fOECT (Figure 1E) in the plant model of this study (devil's ivy) did not trigger obvious adverse responses (Figure S7; Discussion S1) because of the minimal invasiveness and good biological/mechanical compatibility of the conductive PEDOT:PSS microfiber (Young's modulus of 580 MPa). In contrast, the implantation of a rigid stainless-steel needle of a similar diameter caused serious necrosis and the formation of a callus due to the huge mismatch between the Young's moduli of steel and soft plant tissue (100 GPa scale vs. 10 MPa scale). If a rigid implantable device was used, then the tissue damage would lead to false observations, and the callus surrounding it would comprise the signal quality.[25] The conductivity and g_m of the M^+-fOECT also remained stable, promising long-term *in planta* monitoring (Figure 1F).

Transpiration-driven xylem flow monitored by M^+-fOECT

Currently, the cation concentration is determined using the destructive extraction of plant sap, which is not continuous and thus unable to resolve the rapid physiological fluctuation of cations. In contrast, the M^+-fOECT can quickly respond to cation variation (Figure 2A), with the percentage of I_{ds} change being linearly proportional to the logarithmic increase of the cation concentration with a sensitivity of 116.7 μA/dec.

Plant transpiration involves water transport (together with ions and nutrients) from roots through xylem vessels driven by the negative pressure resulting from water vapor evaporation through stomata on leaves. In order to monitor transpiration-driven xylem flow in a rootless plant cultivated in 75 mM KCl solution, two fOECTs were threaded into the stem (Figure 2B). Cation concentrations at both recording sites reached an equilibrium (~70 mM) slightly below the K^+ concentration in the cultivation solution with a τ of ~355 s, which is much faster than the τ calculated based on the 1D diffusion model (~1.3 days). Thus, the fast transport of K^+ by xylem flow shall follow the transpiration-driven 1D convection model.[26] Given that the M^+-fOECT reports the average K^+ concentration in the entire stem, and assuming it quickly follows the change in the xylems due to fast lateral diffusion, the K^+ concentration in the stem (C) as the function of position (x) and time (t) can be described by (see Figure S8 and Discussion S2)

$$\frac{\partial C(x,t)}{\partial t} = -ur\frac{\partial C(x,t)}{\partial x},$$

where u is the xylem flow rate and r is the area percentage of xylem vessels (r = 0.012; Figure S9). The model fits the measurements from both fOCETs well with a u of ~0.35 cm/s.

We further validated that the M^+-fOECT can faithfully detect the changes of xylem flow rate upon adjusting transpiration levels by controlling the opening and closure of stomata (Figure 2C). The xylem flow completely halted immediately after blocking stomata by covering both sides of the leaves and was fully restored immediately after removing the covering tapes. Evidently, the fOCET-M^+ shall enable unprecedented studies of transpiration dynamics and signaling pathways that control stomatal guard cells. As proof-of-concept demonstrations, stomatal dynamics regulated by an environmental stimulus (exposure to simulated sunlight) and plant hormone (abscisic acid [ABA]) were monitored in real time. Interestingly, light-induced stomatal opening (indicated by the sudden acceleration of the xylem flow) exhibited a long delay (~1,000 s), while ABA-induced stomatal closure (sudden suppression of the xylem flow) showed a shorter delay (~450 s) and subsequent recovery after an additional ~640 s (see Figure S10 and Discussion S3). As shown in Figure S11, the 1D convection model can well fit the experimental measurements and derive the xylem flow rates under these distinct transpiration conditions, indicating the generalizability of the model and the reliability of the measurement (Discussion S4).

Detection of H_2O_2 wave induced by different stressors using H_2O_2-fOECT

To continuously monitor variation of H_2O_2 in plants, we devised an H_2O_2-responsive fOECT (H_2O_2-fOECT) with a PEDOT:PSS fiber as the channel and a flexible Au microfiber (250 μm) functionalized with PtNPs as the electrochemical gate.[14] PtNPs (~100 nm; Figure 3A) are stable and efficient electrocatalysts for the oxidation of H_2O_2 with high selectivity (Figure S12). Nafion coating secures PtNPs in place and prevents the capacitive accumulation of anions on the positively biased gate electrode.[27] Otherwise, cations will be driven into the

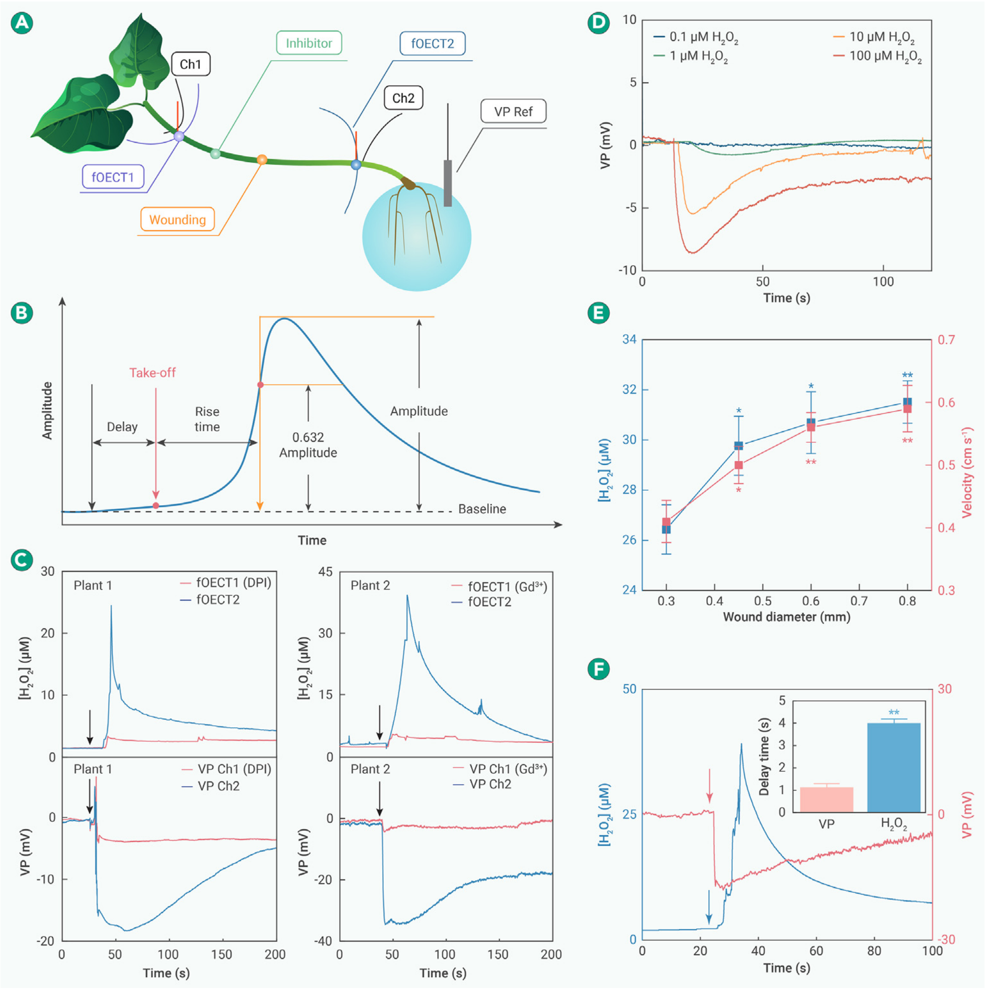

Figure 4. Interdependence of H_2O_2 waves and VP (A) H_2O_2 and VP waves recorded by two H_2O_2-fOECTs (2 cm apart) and two PEDOT:PSS microfibers (Ch1 and Ch2) adjacent to the fOECTs, respectively, in response to wounding inflicted in the middle of the two recording sites after inhibitor (DPI or Gd^{3+}) was infused in the middle of wounding site and fOECT1. (B) Definition of kinetic characteristics. Black arrow: stimulation time, red arrow: take-off point (signal = 5× of baseline fluctuation), and yellow arrow: time point when signal reaches $1-1/e$ (0.632) of the peak. (C) VP and H_2O_2 waves simultaneously recorded on the same plant after applying DPI or Gd^{3+}. (D) Exogenous H_2O_2 (100 μL) induced VP. (E) Amplitude and velocity of H_2O_2 waves depend on stress intensity and are positively correlated with each other. H_2O_2 waves were recorded by two fOECTs (1 cm apart), responding to wounding (1 cm away from the first fOECT) inflicted by needles of different diameters. The velocity is calculated based on the time difference between the take-off points of H_2O_2 waves recorded by the fOECTs. (F) Simultaneous measurement of VP and H_2O_2 waves induced by the same wounding that was applied 1 cm away. Inset shows statistics of the delay time, which is defined as the duration from the moment of wounding to the take-off point. The data in (E) and (F) (inset) are presented as mean ± SD (n = 3 plants) and analyzed using a one-sample t test (*$p < 0.05$ and **$p < 0.01$).

conducting channel, thereby leading to an erroneous signal, particularly when the cation concentration is high. Nafion shielding and the Faradaic reaction at the gate (see Figure 3B and the reaction equations below) allow the fOECT to selectively respond to H_2O_2 but not to high concentrations of cations (Figure S4B):

$$\text{gate: } H_2O_2 - 2e^- = O_2 + 2H^+ \text{ and}$$

$$\text{channel: } PEDOT^+PSS^- + e^- + M^+ = PEDOT^0 + PSS^-M^+.$$

In a 10 μM H_2O_2 solution, the H_2O_2-fOECT reaches the highest g_m of 0.53 mS at V_g = ~0.35 V (Figure 3C), which is therefore used for all H_2O_2 recording experiments. The device-to-device variation of g_m is small (Figure S5B), ensuring consistent measurements. As shown in Figure 3D, the percentage increase of I_{sd} is linearly proportional to the logarithmic increase of the H_2O_2 concentration with a sensitivity of 0.109 mA/dec and an extrapolated low detection limit of 20 nM at S/N = 3. Evidently, the H_2O_2-fOECT is a reliable, sensitive, and fast device for monitoring H_2O_2 variation in plants.

In contrast to the current fluorescence imaging methods, the H_2O_2-fOECT can spatiotemporally resolve the generation and propagation of H_2O_2 in deep plant tissue. A sharp H_2O_2 wave was registered when wounding was applied 1 cm away with a short delay of 2.3 s (Figure 3E). In contrast, mild heating stress (45°C, 1 min) resulted in a relatively slow yet sustained H_2O_2 wave (Discussion S5). Lastly, the H_2O_2 response triggered by the flg22 peptide[28] (the most conserved domain of bacterial flagellin) exhibited the slowest kinetics due to the intricate and protracted signaling pathways involved (see Figure S13 and Discussion S5). Evidently, H_2O_2-fOCET can serve as a unique tool to scrutinize the H_2O_2 signaling kinetics and pathways.

Interdependence of H_2O_2 wave and VP signal and stress-intensity dependence

It is well accepted that H_2O_2 production relies on respiratory burst oxidase homolog D (RBOHD), which can be activated by stressors and Ca^{2+}.[6,29–31] It is also well recognized that H_2O_2 opens Ca^{2+} channels[32–34] and Ca signaling is coupled with VP.[4,31,35] Several theories have been proposed to explain the propagation of the H_2O_2 wave: (theory I) the H_2O_2 burst triggers RBOHDs on neighboring cells to produce the subsequent H_2O_2 burst (self-propagating),[6,36,37] (theory II) propagating VP opens voltage-dependent Ca^{2+} channels along the way, and the generated Ca^{2+} signal produces propagating H_2O_2 waves,[3,38] and (theory III) Ca^{2+} travels through symplastic or apoplastic routes and initiate H_2O_2 regeneration in neighboring cells.[3,39] The mechanism of VP generation and propagation is also under debate. It has been hypothesized that VP is self-regenerative and propagates similarly to the action potential in neurons (hypothesis A),[40] the hydraulic pressure wave generated in the stimulated zone activates mechano-sensitive ion channels and drives the regeneration and propagation of VP (hypothesis B),[41,42] and the produced stress substances travel through xylem vessels and stimulate the regeneration of VP along the way (hypothesis C).[41,43]

To investigate these conjectures, we selectively blocked proteins that are essential to H_2O_2 and VP generation and measured both signals on the same plants with the microfiber electronics. Two H_2O_2-fOECTs were threaded through the plant stem, and wounding was inflicted in the middle between the fOECTs after infusing an RBOHD inhibitor (DPI) through a previously created incision halfway between fOECT1 and the wounding site (Figure 4A). The kinetics of H_2O_2 and VP waves was analyzed based on the characteristics defined in Figure 4B. fOECT2 registered a strong H_2O_2 wave, whereas the propagation of the H_2O_2 wave to fOECT1 was blocked because of the inhibition of RBOHD on its pathway. Two PEDOT:PSS microfibers implanted adjacent to the fOECTs were used to simultaneously record VP in response to the same wounding applied in the middle. Similarly, VP was only recorded at fOECT2 but not fOECT1 (Figure 4C), indicating that blocking RBOHD-dependent H_2O_2 generation also prevents the generation and propagation of VP.

It is known that Ca^{2+} influx through H_2O_2-activable Ca^{2+} channels elicits the internal release of Ca^{2+} through two-pore cation channels (TPC1) on vacuoles.[44] Subsequently, membrane depolarization is produced because the rapidly increased cytosol Ca^{2+} level inhibits the out pumping of H^+ by H^+-ATPase and induces Cl^- efflux through anion channels.[31,45] The depolarization of many plant cells collectively produces a VP wave. In a similar experiment, Gd^{3+} (a Ca^{2+} channel blocker) was introduced between fOECT1 and the wounding site. It was observed that the propagation of both the H_2O_2 wave and VP to fOECT1 was

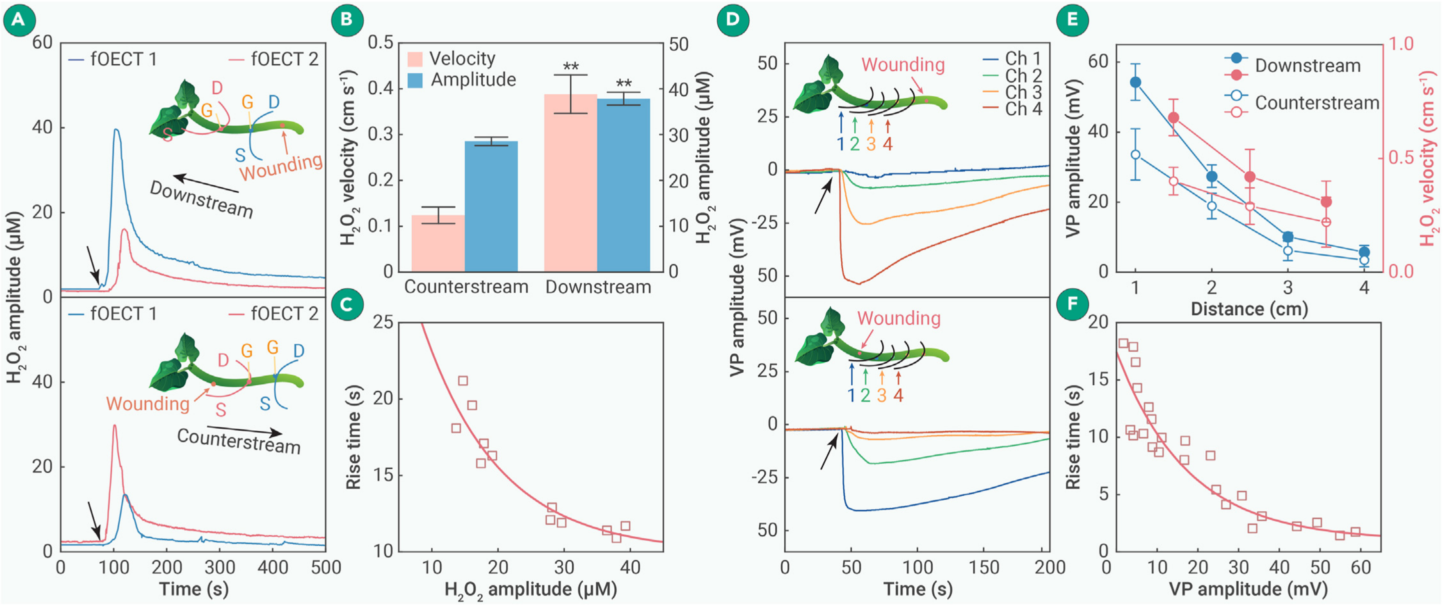

Figure 5. Influence of xylem flow in H_2O_2 and VP propagation (A) The H_2O_2 waves elicited by wounding applied either 1 cm below or above H_2O_2-fOECTs. (B) H_2O_2 wave amplitude and velocity in downstream and counterstream directions. (C) Rise time decreases with H_2O_2 amplitude. (D) VP waves elicited by wounding applied either 1 cm below or above 4 PEDOT:PSS microfibers. (E) VP amplitude and velocity decay with distance in both directions. (F) Rise time decreases with VP amplitude. The data in (B) are presented as mean ± SD (*n* = 3 plants) and analyzed using a one-sample t test (***p* < 0.01 vs. counterstream).

obstructed (Figure 4C), suggesting that blocking VP prevents the propagation of the H_2O_2 wave. Note that the infusion of a KCl solution did not prevent the generation and propagation of either the VP or H_2O_2 wave (Figure S14), ruling out the negative impact by infusion itself. Further, a VP-mimicking voltage pulse did not affect the wounding-induced H_2O_2 wave (Figure S15), suggesting that the accompanying VP does not electrically interfere with the H_2O_2 recording by the fOECT (see Discussion S6). A direct comparison of paired recordings on the same plant and the fact that all the observed signals are dynamic responses to a specific physiological context rule out the possibility of false observations due to interferences. Moreover, the simultaneous recording of VP and H_2O_2 waves on the same plant unambiguously demonstrates the interdependence and intimate coupling between the two waves.

The observed interdependence of H_2O_2 and VP waves denies hypothesis A for VP and theory I for H_2O_2, and theory I is also challenged by the fact that the intercellular diffusion of H_2O_2 is largely prevented by the abundant existence of antioxidants in apoplast.[46] Moreover if theory II for H_2O_2 were true, then wounding-triggered self-propagating VP should guarantee H_2O_2 propagation through the RBOHD-blocked site. Further, the observed critical involvement of Ca^{2+} channels for H_2O_2 propagation is not reflected in H_2O_2 theory III.

To further investigate whether H_2O_2 is sufficient for VP generation, we recorded VP waves when H_2O_2 of various amounts was infused at 1 cm away (Figure 4D). Interestingly, exogeneous H_2O_2 can not only trigger VP but also determines the amplitude and rising kinetics of VP in a dose-dependent manner, suggesting a stress-intensity dependence. This experiment denies VP hypothesis B because H_2O_2 stimulation alone (without the exertion of pressure) can trigger propagating VP.

Conceivably from Figure 4D, a higher H_2O_2 concentration leads to wider spreading of H_2O_2, hence evoking a stronger VP as the collective response from more plant cells. Also conceivably, the stronger the initially triggered VP, the faster speed it travels because it more potently triggers the regeneration mechanism that involves both RBOHD and Ca^{2+} channels. Viewing the intimate coupling between H_2O_2 and VP generation, we further hypothesize that stress intensity also correlates with H_2O_2 wave propagation. To investigate our hypothesis, H_2O_2 waves were recorded by two fOECTs threaded into the plant (1 cm apart) upon wounding with needles of different diameters. As expected, both the amplitude and propagation velocity positively scale with the stress intensity (Figure 4E). In addition, the amplitude and velocity are positively correlated with each other, suggesting that the propagation kinetics depends on signal intensity.

Subsequently, we conducted simultaneous recordings of wounding-induced VP and H_2O_2 waves at the same location to compare their kinetics (Figure 4F). VP and H_2O_2 are closely accompanied by each other. The former is always faster than the latter and rises more steeply, presumably because H_2O_2 generation is a slower process involving the RBHOD-catalyzed reaction. Both VP and H_2O_2 waves have a similar long-lasting tail following the rapid rise. Altogether, our observations suggest that the generations of H_2O_2 and VP are interdependent and mutually reinforcing, with the latter having a faster kinetics (faster rise and velocity), and that the amplitude and propagation velocity of both VP and H_2O_2 waves positively correlate with the initial stress intensity.

Influence of xylem flow in H_2O_2 and VP propagation

To investigate the influence of transpiration-driven xylem flow on the propagation of H_2O_2 waves, wounding-induced signals traveling downstream (toward leaves as xylem flow) or counterstream were recorded by two H_2O_2-fOECTs implanted 1 and 2 cm away from the wounding site (Figure 5A). The downstream velocity was substantially higher than the counterstream velocity (0.39 vs. 0.16 cm/s; Figure 5B), suggesting that H_2O_2 transport by xylem flow greatly promotes the propagation of H_2O_2 waves beyond passive diffusion. Even the counterstream velocity is too fast to be explained by H_2O_2 theory I which attributes H_2O_2 propagation to slow H_2O_2 spreading alone. Our observation suggests the importance of VP to reinforce and accelerate H_2O_2 propagation. The downstream velocity is similar to the xylem flow rate determined by the M^+-fOECT, implying the dominating influence of the xylem flow in downstream propagation. The H_2O_2 amplitude is also significantly smaller in the counterstream direction, further corroborating the correlation between propagation kinetics and signal intensity. The direction dependence of H_2O_2 propagation cannot be explained by H_2O_2 theory I, II, or III. Theory III is also questioned by the fact that apoplastic diffusion of Ca^{2+} is largely prevented by its binding with abundant carboxyl groups from pectin on cell wall.[47] It has been demonstrated that Ca^{2+} can reach adjacent cells by symplastic transportation via plasmodesmata (PDs),[39,48,49] which explains the counterstream regeneration and propagation of H_2O_2 waves. The rise time of H_2O_2 waves measured at various distances in both directions shows a robust negative correlation with its amplitude (Figure 5C), further strengthening our hypothesis of kinetic dependence on intensity. The exponential extrapolation suggests that the shortest possible rise time is ~9.9 s, indicating the kinetic limit of H_2O_2 generation.

To investigate the influence of xylem flow on VP propagation, four PEDOT:PSS microfibers were threaded through the plant stem with equal spacing of 1 cm. VPs were sequentially registered by these microfibers after wounding being applied first downstream then counterstream at 1 cm away from the nearby fiber (Figure 5D). VP travels notably faster in the downstream direction (Figure 5E),

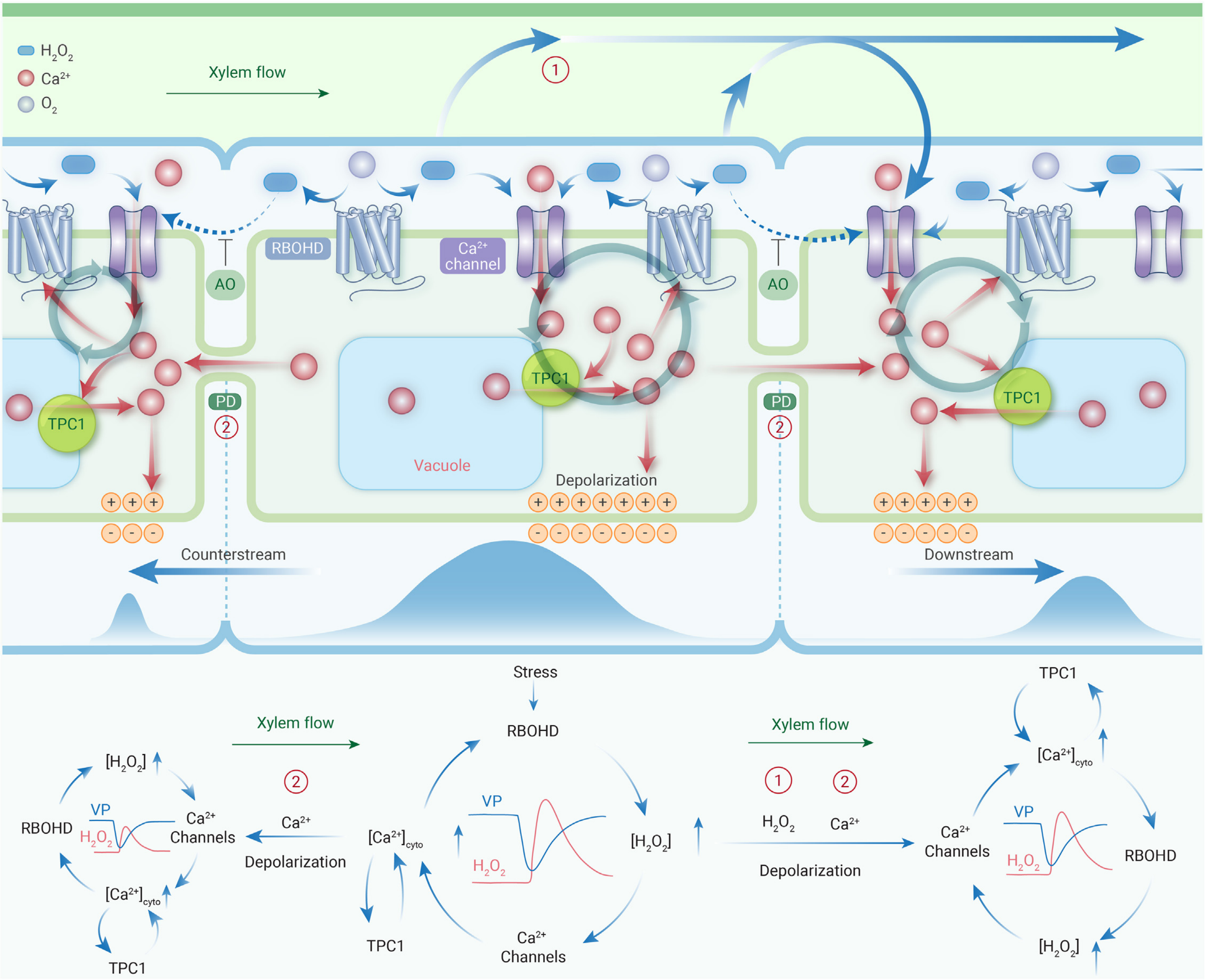

Figure 6. Illustration of transpiration-dependent H_2O_2-VP mutual-reinforcing propagation ① and ② indicate xylem transport of H_2O_2 and Ca^{2+} transport through PDs, respectively. AO, antioxidant; TPC1, the two-pore channel 1; PDs, plasmodesmata.

suggesting that VP propagation is greatly facilitated by H_2O_2 transport by the xylem flow. The VP velocity (up to 0.68 cm/s) is much slower than a pressure wave (up to 1,500 m/s),[50] disproving VP hypothesis B. The observed direction-dependent propagation cannot be explained by VP hypothesis A or B. Note that hypothesis C, which explains VP propagation by the transport of stress substances through xylem vessels, is not able to explain its counterstream propagation. Conceivably, it is Ca^{2+} ions transported from the already excited cells to the counterstream cells that are responsible for triggering the H_2O_2-VP mutual-reinforcing generation and propagation in the counterstream direction. However, the critical involvement of the Ca^{2+} channel (Figure 4C) suggests that Ca^{2+} ions through PDs are not sufficient to trigger the mutual-reinforcing loop. Plausibly, they activate RBOHDs, which in turn open Ca^{2+} channels to admit more Ca^{2+} ions. Thereby, the abundant release of Ca^{2+} from vacuoles becomes possible, permitting the regeneration of H_2O_2 and VP wave events in the counterstream direction.

Both the amplitude and propagation velocity of H_2O_2 and VP waves exponentially decrease with travel distance (Figures 5E and S16) with similar decay constants (2.55 vs. 2.54 cm for velocity decay and 1.53 vs. 1.29 cm for amplitude decay). Such similar decay indicates that the regeneration and propagation of both waves are intimately coupled, and the observed distance decay challenges H_2O_2 theory II and VP hypothesis A. Similar to H_2O_2 waves, the rise time of VP shows a strong negative correlation with its amplitude (Figure 5F). The extrapolated minimum of 0.72 s indicates the kinetic limit of VP generation, which is faster than that of H_2O_2.

The proposed new mechanism

Based on our experimental results and some well-recognized notions, we propose a transpiration- and intensity-dependent H_2O_2-VP mutual-reinforcing propagation mechanism (Figure 6). Specifically, stress-stimulated RBOHDs produce H_2O_2, which subsequently activates Ca^{2+} channels, and the resulting Ca^{2+} influx stimulates internal Ca^{2+} release from vacuoles through TPC1 channels. The increased intracellular Ca^{2+} further stimulates RBOHDs to produce more H_2O_2 and causes membrane depolarization by inhibiting the out pumping of H^+ and inducing Cl^- efflux. Therefore, a positive-feedback loop between H_2O_2 production and membrane depolarization is established, ensuring their fast development. The synchronized excitation of many cells collectively produces H_2O_2 and VP waves. The kinetics of the former slightly lags behind because of the involvement of biochemical reactions. Stronger stress leads to more cells being excited, hence the higher wave amplitudes.

Transported by xylem flow, the produced H_2O_2 spreads to the downstream cells to trigger the mutual-reinforcing regeneration of H_2O_2 and VP waves. H_2O_2 is difficult to reach adjacent cells through apoplastic pathway due to the abundant presence of antioxidants (e.g., ascorbic acid). This disapproves theory

I for H_2O_2 propagation and suggests that the counterstream propagation of VP and H_2O_2 waves is not dependent on the apoplastic diffusion of H_2O_2. On the other hand, Ca^{2+} ions can reach both downstream and counterstream cells via PDs, thereby reinforcing the downstream generation of both waves and enabling their counterstream regeneration. Ca^{2+} channels are critical to the H_2O_2-VP mutual-reinforcing (positive-feedback) loop. In the counterstream direction, they are opened by PD-transported Ca^{2+} ions through RBOHD mediation. However, further studies are required to unambiguously test this PD-dependent hypothesis.

The higher the amplitude of the H_2O_2 or VP wave, the faster its kinetics (faster propagation velocity and rise time) because the mutual-reinforcing loop is more potently evoked and more messengers (H_2O_2 and Ca^{2+}) reach the neighboring cells. Due to the long-distance H_2O_2 signaling enabled by the xylem flow, the downstream waves exhibit higher amplitude and velocity, but even the counterstream velocity of both waves is too fast to be explained by the mechanisms purely relying on the diffusion of H_2O_2 (theory I) or Ca^{2+} (theory III), suggesting that H_2O_2-VP mutual reinforcement is critical to the fast generation and propagation of both waves. In both directions, the amplitudes and velocities of the regenerated H_2O_2 and VP waves concomitantly decay with distance.

By simultaneously recording VP and H_2O_2 waves and recording on different locations on the same plant with high temporospatial resolution, we provide strong and direct evidence to show that (1) H_2O_2 and VP waves are critically interdependent (i.e., inhibition of one prevents the other) and (2) they are intimately coupled (i.e., coupled kinetics, similar directional dependence, similar decay with distance, amplitude correlation, and H_2O_2 alone can stimulate VP in a dose-dependent manner). The herein proposed mechanism can fully explain all of our observations, whereas the previously proposed theories or hypotheses are denied by multiple observations from our experiments.

CONCLUSION

The flexible microfiber-shaped OECT offers a versatile platform for the *in situ* study of dynamic physiological processes in plants with high temporospatial resolution. Using two different gate electrodes, cation-responsive and H_2O_2-responsive fOECTs were herein devised. The gate can be otherwise engineered to selectively sense a range of chemicals or molecules, for example, incorporating redox enzymes for detecting their substrates. Microfiber electronics presents distinct advantages over conventional imaging-based tools, including sub-second temporal resolution, the capacity for deep-tissue exploration, and a chronically stable bioelectronic interface for longitudinal studies.

Utilizing microfiber electronics to record H_2O_2 and VP waves on the same plants, we unveiled the fundamental mechanisms underlying these dynamic processes, revealing an interdependent and mutual-reinforcing positive-feedback loop and intensity-dependent kinetics. Moreover, we discovered that both H_2O_2 and VP waves propagate faster toward the leaves than toward the roots because of the directional long-distance transport of H_2O_2 in the xylem.

The herein proposed transpiration- and intensity-dependent H_2O_2-VP mutual-reinforcing propagation theory shall inspire further research on the coordinated response to stresses and long-distance signaling enabled by xylem flow. In addition, the observed kinetic differences of distinct signaling cascades that control stomatal opening/closing and H_2O_2 wave generation are carefully discussed in the supplemental information. However, the observed interesting phenomena warrant further investigation. We envision that by combining microfiber electronics with molecular and genetic approaches, the cascaded signaling pathways underlying various physiological processes in plants can be precisely deciphered.

DATA AND CODE AVAILABILITY

Data are available from the corresponding author upon reasonable request.

REFERENCES

1. Smirnoff, N. and Arnaud, D. (2019). Hydrogen peroxide metabolism and functions in plants. *New Phytol.* **221**:1197–1214. DOI:https://doi.org/10.1111/nph.15488.
2. Baxter, A., Mittler, R. and Suzuki, N. (2014). ROS as key players in plant stress signalling. *J. Exp. Bot.* **65**:1229–1240. DOI:https://doi.org/10.1093/jxb/ert375.
3. Choi, W.G., Miller, G., Wallace, I. et al. (2017). Orchestrating rapid long-distance signaling in plants with Ca2+ , ROS and electrical signals. *Plant J.* **90**:698–707. DOI:https://doi.org/10.1111/tpj.13492.
4. Choi, W.G., Hilleary, R., Swanson, S.J. et al. (2016). Rapid, Long-Distance Electrical and Calcium Signaling in Plants. *Annu. Rev. Plant Biol.* **67**:287–307. DOI:https://doi.org/10.1146/annurev-arplant-043015-112130.
5. Fichman, Y. and Mittler, R. (2021). Integration of electric, calcium, reactive oxygen species and hydraulic signals during rapid systemic signaling in plants. *Plant J.* **107**:7–20. DOI:https://doi.org/10.1111/tpj.15360.
6. Miller, G., Schlauch, K., Tam, R. et al. (2009). The plant NADPH oxidase RBOHD mediates rapid systemic signaling in response to diverse stimuli. *Sci. Signal.* **2**:ra45. DOI:https://doi.org/10.1126/scisignal.2000448.
7. Orozco-Cardenas, M. and Ryan, C.A. (1999). Hydrogen peroxide is generated systemically in plant leaves by wounding and systemin via the octadecanoid pathway. *Plant J.* **96**:6553–6557. DOI:https://doi.org/10.1073/pnas.96.11.6553.
8. Fichman, Y., Miller, G. and Mittler, R. (2019). Whole-Plant Live Imaging of Reactive Oxygen Species. *Mol. Plant* **12**:1203–1210. DOI:https://doi.org/10.1016/j.molp.2019.06.003.
9. Zheng, D.-J., Yang, Y.-S. and Zhu, H.-L. (2019). Recent progress in the development of small-molecule fluorescent probes for the detection of hydrogen peroxide. *TrAC, Trends Anal. Chem.* **118**:625–651. DOI:https://doi.org/10.1016/j.trac.2019.06.031.
10. Swanson, S.J., Choi, W.-G., Chanoca, A. et al. (2011). In vivo imaging of Ca2+, pH, and reactive oxygen species using fluorescent probes in plants. *Annu. Rev. Plant Biol.* **62**:273–297. DOI:https://doi.org/10.1146/annurev-arplant-042110-103832.
11. Lew, T.T.S., Koman, V.B., Silmore, K.S. et al. (2020). Real-time detection of wound-induced H2O2 signalling waves in plants with optical nanosensors. *Nat. Plants* **6**:404–415. DOI:https://doi.org/10.1038/s41477-020-0632-4.
12. Meinzer, F.C., Brooks, J.R., Domec, J.C. et al. (2006). Dynamics of water transport and storage in conifers studied with deuterium and heat tracing techniques. *Plant Cell Environ.* **29**:105–114. DOI:https://doi.org/10.1111/j.1365-3040.2005.01404.x.
13. Luo, Y., Li, W., Lin, Q. et al. (2021). A Morphable Ionic Electrode Based on Thermogel for Non-Invasive Hairy Plant Electrophysiology. *Adv. Mater.* **33**:2007848. DOI:https://doi.org/10.1002/adma.202007848.
14. Yao, Y., Huang, W., Chen, J. et al. (2023). Flexible and stretchable organic electrochemical transistors for physiological sensing devices. *Adv. Mater.* **35**:2209906. DOI:https://doi.org/10.1002/adma.202209906.
15. Paulsen, B.D., Tybrandt, K., Stavrinidou, E. et al. (2020). Organic mixed ionic–electronic conductors. *Nat. Mater.* **19**:13–26. DOI:https://doi.org/10.1038/s41563-019-0435-z.
16. Fratini, S., Nikolka, M., Salleo, A. et al. (2020). Charge transport in high-mobility conjugated polymers and molecular semiconductors. *Nat. Mater.* **19**:491–502. DOI:https://doi.org/10.1038/s41563-020-0647-2.
17. Wang, Y., Wustoni, S., Surgailis, J. et al. (2024). Designing organic mixed conductors for electrochemical transistor applications. *Nat. Rev. Mater.* **9**:249–265. DOI:https://doi.org/10.1038/s41578-024-00652-7.
18. Rivnay, J., Inal, S., Salleo, A. et al. (2018). Organic electrochemical transistors. *Nat. Rev. Mater.* **3**:1–14. DOI:https://doi.org/10.1038/natrevmats.2017.86.
19. Saleh, A., Koklu, A., Uguz, I. et al. (2024). Bioelectronic interfaces of organic electrochemical transistors. *Nat. Rev. Bioeng.* **2**:559–574. DOI:https://doi.org/10.1038/s44222-024-00180-7.
20. Yang, A., Li, Y., Yang, C. et al. (2018). Fabric organic electrochemical transistors for biosensors. *Adv. Mater.* **30**:1800051. DOI:https://doi.org/10.1002/adma.201800051.
21. Kim, Y., Lim, T., Kim, C.H. et al. (2018). Organic electrochemical transistor-based channel dimension-independent single-strand wearable sweat sensors. *NPG Asia Mater.* **10**:1086–1095. DOI:https://doi.org/10.1038/s41427-018-0097-3.
22. Wang, C., Xia, K., Zhang, Y. et al. (2019). Silk-based advanced materials for soft electronics. *Acc. Chem. Res.* **52**:2916–2927. DOI:https://doi.org/10.1021/acs.accounts.9b00333.
23. Marks, A., Griggs, S., Gasparini, N. et al. (2022). Organic Electrochemical Transistors: An Emerging Technology for Biosensing. *Adv. Mater. Interfaces* **9**:2102039. DOI:https://doi.org/10.1002/admi.202102039.
24. Groenendaal, L., Zotti, G., Aubert, P.H. et al. (2003). Electrochemistry of poly (3, 4-alkylenedioxythiophene) derivatives. *Adv. Mater.* **15**:855–879. DOI:https://doi.org/10.1002/adma.200300376.
25. Kong, L., Wen, H., Luo, Y. et al. (2023). Dual-Conductive and Stiffness-Morphing Microneedle Patch Enables Continuous In Planta Monitoring of Electrophysiological Signal and Ion Fluctuation. *ACS Appl. Mater. Interfaces* **15**:43515–43523. DOI:https://doi.org/10.1021/acsami.3c08783.
26. Jensen, K.H., Berg-Sørensen, K., Bruus, H. et al. (2016). Sap flow and sugar transport in plants. *Rev. Mod. Phys.* **88**:035007. DOI:https://doi.org/10.1103/RevModPhys.88.035007.
27. Nawaz, A., Liu, Q., Leong, W.L. et al. (2021). Organic electrochemical transistors for *in vivo* bioelectronics. *Adv. Mater.* **33**:2101874. DOI:https://doi.org/10.1002/adma.202101874.
28. Li, L., Li, M., Yu, L. et al. (2014). The FLS2-associated kinase BIK1 directly phosphorylates the NADPH oxidase RbohD to control plant immunity. *Cell Host Microbe* **15**:329–338. DOI:https://doi.org/10.1016/j.chom.2014.02.009.
29. Choudhury, F.K., Rivero, R.M., Blumwald, E. et al. (2017). Reactive oxygen species, abiotic stress and stress combination. *Plant J.* **90**:856–867. DOI:https://doi.org/10.1111/tpj.13299.
30. Kadota, Y., Shirasu, K. and Zipfel, C. (2015). Regulation of the NADPH oxidase RBOHD during plant immunity. *Plant Cell Physiol.* **56**:1472–1480. DOI:https://doi.org/10.1093/pcp/pcv063.
31. Tian, W., Wang, C., Gao, Q. et al. (2020). Calcium spikes, waves and oscillations in plant development and biotic interactions. *Nat. Plants* **6**:750–759. DOI:https://doi.org/10.1038/s41477-020-0667-6.
32. Mori, I.C. and Schroeder, J.I. (2004). Reactive oxygen species activation of plant Ca2+ channels. A signaling mechanism in polar growth, hormone transduction, stress signaling, and hypothetically mechanotransduction. *Plant Physiol.* **135**:702–708. DOI:https://doi.org/10.1104/pp.104.042069.

33. Xu, G., Moeder, W., Yoshioka, K. et al. (2022). A tale of many families: calcium channels in plant immunity. *Plant Cell* **34**:1551–1567. DOI:https://doi.org/10.1093/plcell/koac033.
34. Laohavisit, A., Shang, Z., Rubio, L. et al. (2012). Arabidopsis annexin1 mediates the radical-activated plasma membrane Ca2+-and K+-permeable conductance in root cells. *Plant Cell* **24**:1522–1533. DOI:https://doi.org/10.1105/tpc.112.097881.
35. Nguyen, C.T., Kurenda, A., Stolz, S. et al. (2018). Identification of cell populations necessary for leaf-to-leaf electrical signaling in a wounded plant. *Proc. Natl. Acad. Sci. USA* **115**:10178–10183. DOI:https://doi.org/10.1073/pnas.1807049115.
36. Mittler, R., Vanderauwera, S., Suzuki, N. et al. (2011). ROS signaling: the new wave? *Trends Plant Sci.* **16**:300–309. DOI:https://doi.org/10.1016/j.tplants.2011.03.007.
37. Suzuki, N., Miller, G., Salazar, C. et al. (2013). Temporal-spatial interaction between reactive oxygen species and abscisic acid regulates rapid systemic acclimation in plants. *Plant Cell* **25**:3553–3569. DOI:https://doi.org/10.1105/tpc.113.114595.
38. Suda, H. and Toyota, M. (2022). Integration of long-range signals in plants: A model for wound-induced Ca2+, electrical, ROS, and glutamate waves. *Curr. Opin. Plant Biol.* **69**:102270. DOI:https://doi.org/10.1016/j.pbi.2022.102270.
39. Fichman, Y., Myers, R.J., Jr., Grant, D.G. et al. (2021). Plasmodesmata-localized proteins and ROS orchestrate light-induced rapid systemic signaling in Arabidopsis. *Sci. Signal.* **14**:eabf0322. DOI:https://doi.org/10.1126/scisignal.abf0322.
40. Vodeneev, V., Akinchits, E. and Sukhov, V. (2015). Variation potential in higher plants: Mechanisms of generation and propagation. *Plant Signal. Behav.* **10**:e1057365. DOI:https://doi.org/10.1080/15592324.2015.1057365.
41. Brenner, E.D., Stahlberg, R., Mancuso, S. et al. (2006). Plant neurobiology: an integrated view of plant signaling. *Trends Plant Sci.* **11**:413–419. DOI:https://doi.org/10.1016/j.tplants.2006.06.009.
42. Farmer, E.E., Gao, Y.Q., Lenzoni, G. et al. (2020). Wound- and mechanostimulated electrical signals control hormone responses. *New Phytol.* **227**:1037–1050. DOI:https://doi.org/10.1111/nph.16646.
43. Evans, M.J. and Morris, R.J. (2017). Chemical agents transported by xylem mass flow propagate variation potentials. *Plant J.* **91**:1029–1037. DOI:https://doi.org/10.1111/tpj.13624.
44. Peiter, E., Maathuis, F.J.M., Mills, L.N. et al. (2005). The vacuolar Ca2+-activated channel TPC1 regulates germination and stomatal movement. *Nature* **434**:404–408. DOI:https://doi.org/10.1038/nature03381.
45. Kumari, A., Chételat, A., Nguyen, C.T. et al. (2019). Arabidopsis H+-ATPase AHA1 controls slow wave potential duration and wound-response jasmonate pathway activation. *Proc. Natl. Acad. Sci. USA* **116**:20226–20231. DOI:https://doi.org/10.1073/pnas.1907379116.
46. Pignocchi, C. and Foyer, C.H. (2003). Apoplastic ascorbate metabolism and its role in the regulation of cell signalling. *Curr. Opin. Plant Biol.* **6**:379–389. DOI:https://doi.org/10.1016/s1369-5266(03)00069-4.
47. Hepler, P.K. and Winship, L.J. (2010). Calcium at the cell wall-cytoplast interface. *J. Integr. Plant Biol.* **52**:147–160. DOI:https://doi.org/10.1111/j.1744-7909.2010.00923.x.
48. Evans, M.J., Choi, W.G., Gilroy, S. et al. (2016). A ROS-Assisted Calcium Wave Dependent on the AtRBOHD NADPH Oxidase and TPC1 Cation Channel Propagates the Systemic Response to Salt Stress. *Plant Physiol.* **171**:1771–1784. DOI:https://doi.org/10.1104/pp.16.00215.
49. Lee, J.-Y., Wang, X., Cui, W. et al. (2011). A plasmodesmata-localized protein mediates cross-talk between cell-to-cell communication and innate immunity in Arabidopsis. *Plant Cell* **23**:3353–3373. DOI:https://doi.org/10.1105/tpc.111.087742.
50. Johns, S., Hagihara, T., Toyota, M. et al. (2021). The fast and the furious: rapid long-range signaling in plants. *Plant Physiol.* **185**:694–706. DOI:https://doi.org/10.1093/plphys/kiaa098.

ACKNOWLEDGMENTS

H.W. acknowledges the research scholarship awarded by the Institute of Flexible Electronics Technology of Tsinghua, Zhejiang (IFET-THU); Nanyang Technological University (NTU); and Qiantang Science and Technology Innovation Center, China (QSTIC). This project is supported by the Singapore Indoor Farming System (SingFarm) CREATE Initiative (024574-00005) from the National Research Foundation of Singapore, and a National University of Singapore Presidential Young Professorship Award (22-4974-A0003).

AUTHOR CONTRIBUTIONS

P.C., H.W., and Y.L. conceived the project, designed the study, and wrote the manuscript. H.W. performed the majority of experiments and data analysis. L.K. assisted with the experiments and data analysis. X.Z. and Y.M. provided plant reagents (e.g., *Flg22*) and helped with result discussions. X.S., Y.M., and X.C. assisted with the experimental design and discussions. All authors have revised the manuscript and given their approval of the final version.

DECLARATION OF INTERESTS

The authors declare no conflicts of interest.

SUPPLEMENTAL INFORMATION

It can be found online at https://doi.org/10.1016/j.xinn.2025.100800.

The Innovation, Volume *6*

Supplemental Information

Mutually reinforcing and transpiration-dependent propagation of H_2O_2 and variation potential in plants revealed by fiber organic electrochemical transistors

Hanqi Wen, Lingxuan Kong, Xinlu Zhu, Yansong Miao, Xing Sheng, Xiaodong Chen, Yuxin Liu, and Peng Chen

Supplemental Information

Mutually reinforcing and transpiration-dependent propagation of H_2O_2 and variation potential in plants revealed by fiber organic electrochemical transistors

Hanqi Wen, Lingxuan Kong, Xinlu Zhu, Yansong Miao, Xing Sheng, Xiaodong Chen, Yuxin Liu, Peng Chen

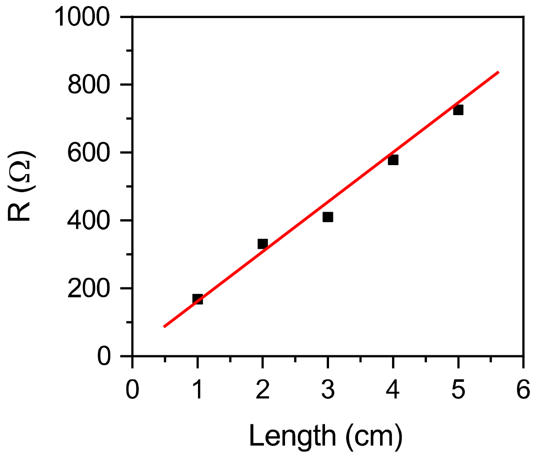

Figure S1. Resistance of the conductive microfiber linearly scales with the length, indicating the uniformity of PEDOT:PSS coating.

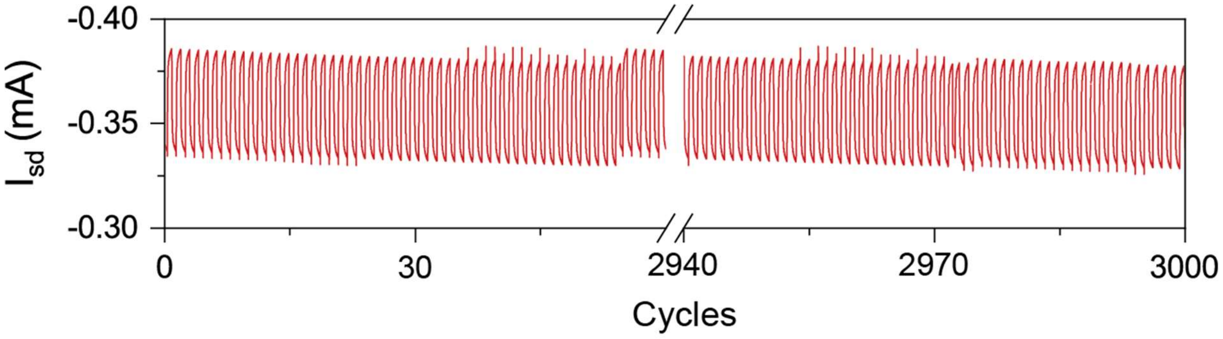

Figure S2. Stability of fOECT-M⁺ after 3000 working cycles. With V_{sd} = -0.2 V, pulsed V_g (amplitude: 0.2 V; pulse width: 1s) was applied for 3000 times, in 5 mM KCl solution.

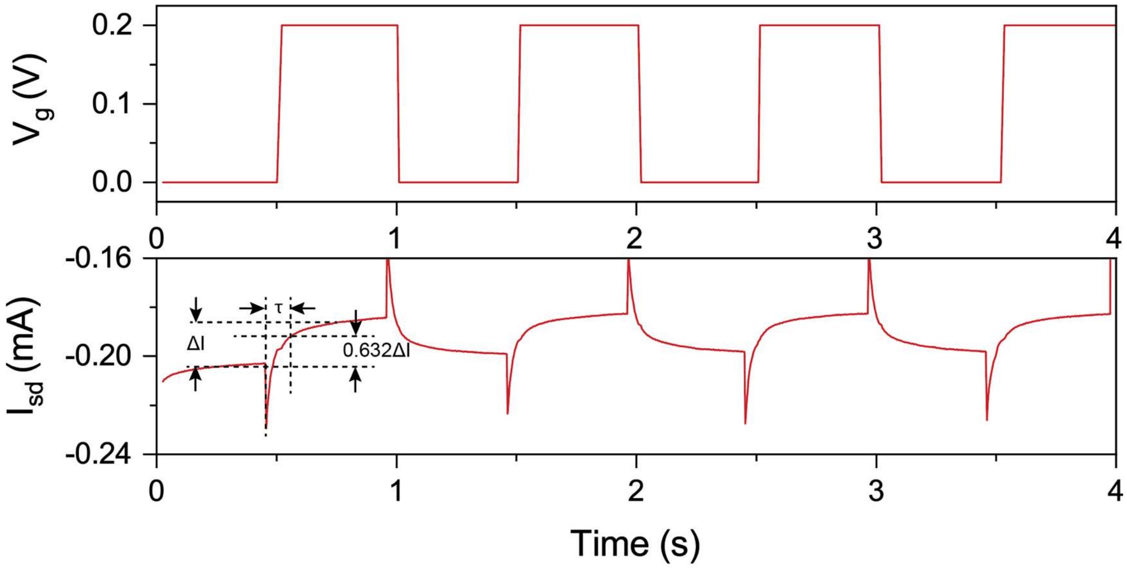

Figure S3. Response time of fOECT-M⁺. fOECT was tested in 5 mM KCl which is typical cation concentration in plant stem tissue. Upon V_g of 0.2 V being applied, a response time constant τ of 0.11s was observed, which is defined as when signal increases by (1-1/e)*100% or 63.2%.

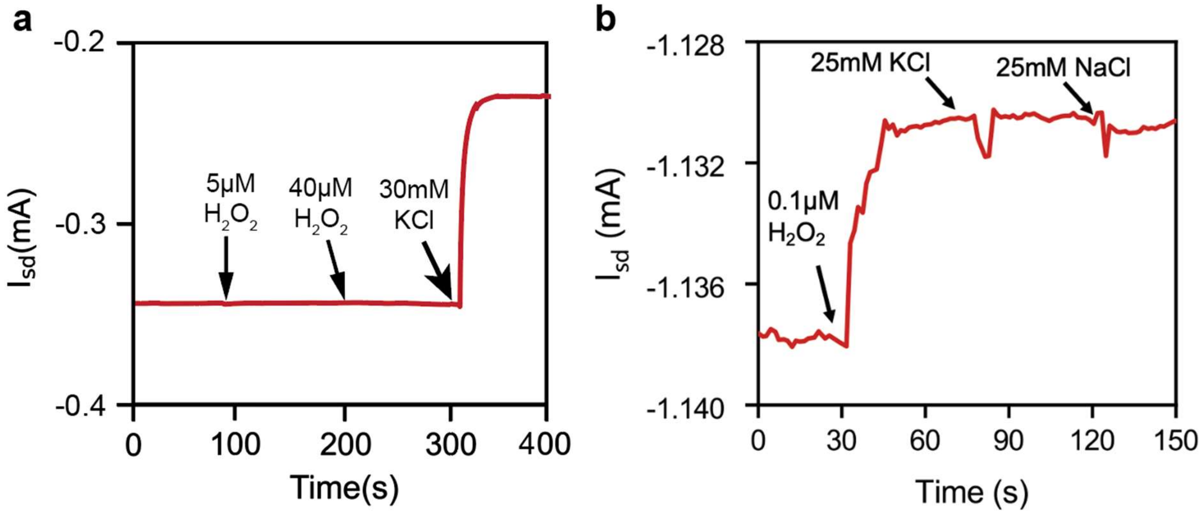

Figure S4. No crosstalk between M^+-fOECT and H_2O_2-fOECT. a, M^+-fOECT is insensitive to H_2O_2**. b,** H_2O_2-fOECT is insensitive to cation.

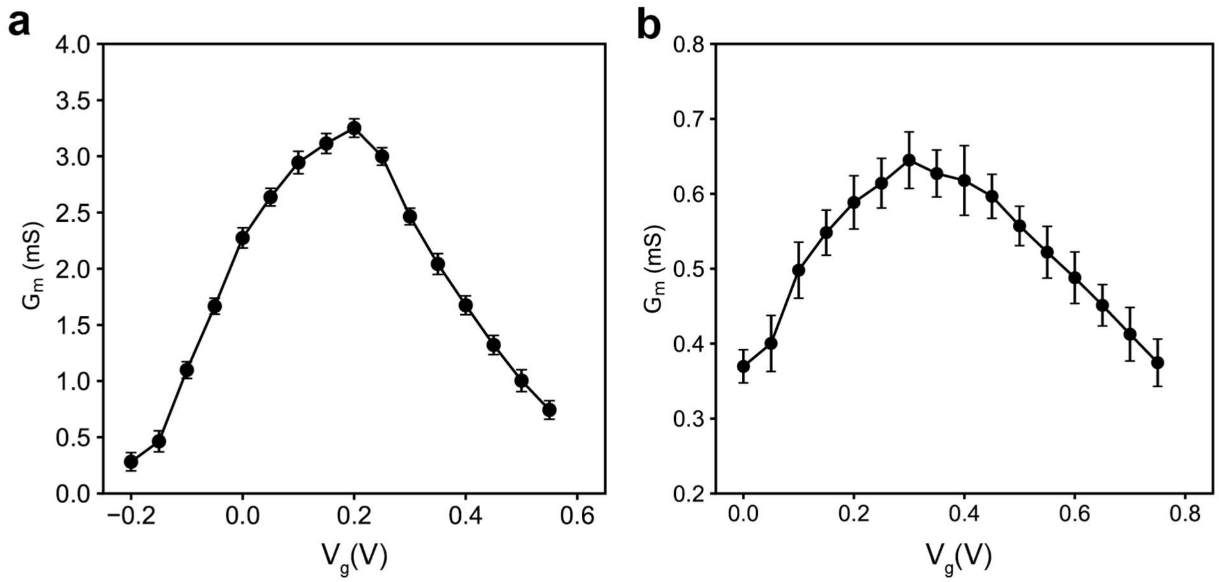

Figure S5. Averaged transconductance curve from five a) M^+-fOECTs and b) H_2O_2-fOECT. For a, fOECTs were tested in 75 mM KCl; for b, fOECTs were tested in 10 μM H_2O_2 solution. The error bars indicate the standard deviations.

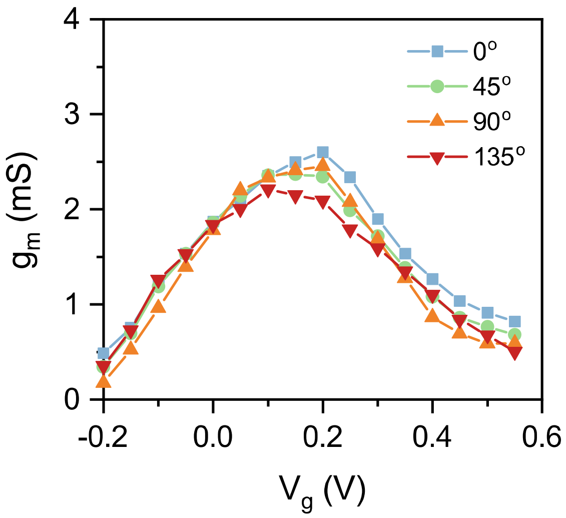

Figure S6. **Bending stability of fOECT-M$^+$**. Transconductance of fOECT (g_m) when the source-drain channel fiber was bent at different angles, in 5 mM KCl solution.

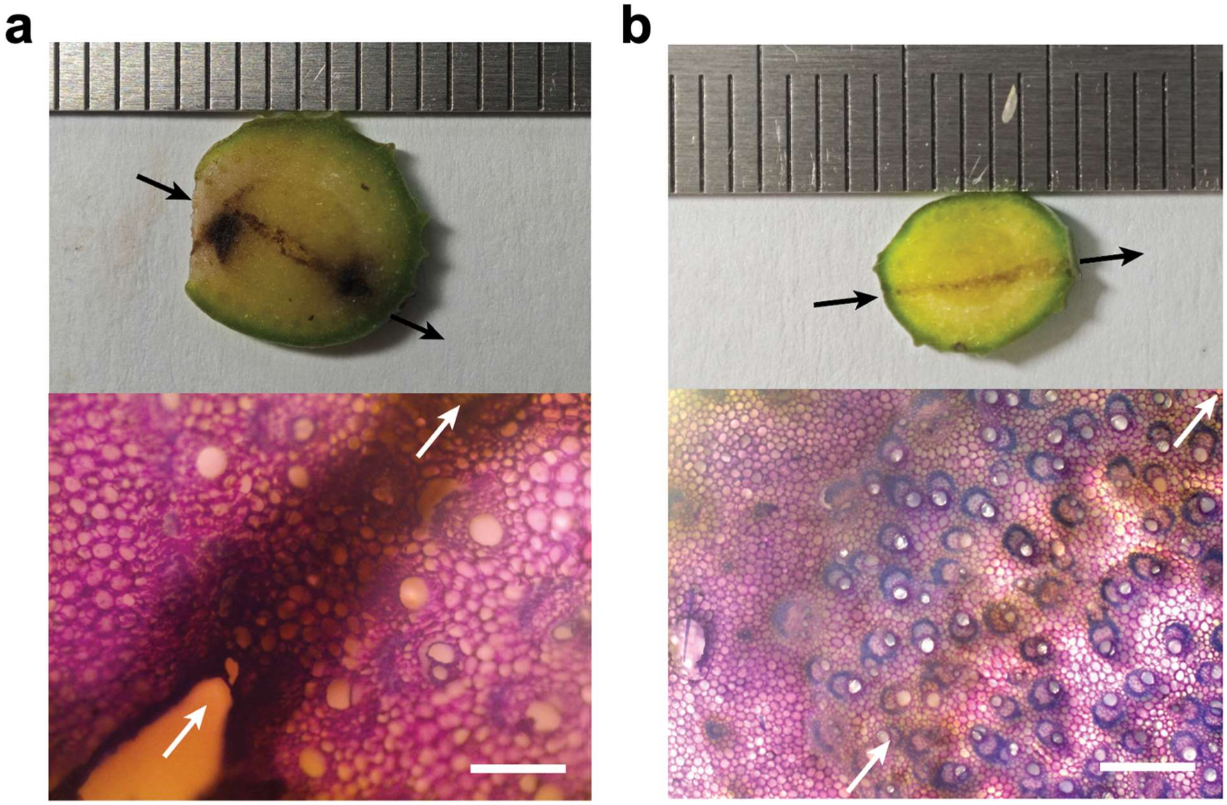

Figure S7. Plant (devil's ivy) reaction to implantation of rigid stainless steel needles (a) and flexible PEDOT:PSS microfibers (b) for 14 days. The cross section of the plan stem where the implantation was applied across it was shown by photograph (top) and micrograph after being stained by toluidine blue O (TBO). Both the microfibers and needlea have a diameter of ~240 μm.

Supplementary discussion 1: Mechanical compatibility of the conductive microfiber is important

Due to the huge mismatch in the Young's modulus (100 GPa scale vs. 10 MPa scale), the plant reacted drastically to the rigid needle as evidenced by necrosis (dark regions in the photograph) and formation of callus (deep purple in TBO staining image). Callus is a mass of undifferentiated plant cells that forms in response to injury or stimulation, serving a protective role by encasing foreign object, sealing the wound, and preventing infection. The serious damage of the plant shall likely lead to false observations. In addition, the

encapsulation of the electrode or device by the callus shall compromise the quality of the signal recording. In contrast, the PEDOT:PSS microfiber, which has a Young's modulus (580 MPa) comparable to the plant tissue, only caused minimal cell response. Specifically, no necrosis and callus were observed. The pale brown line seen in both the photograph and staining image is due to the oxidation of phenolic compounds which are released from the damaged cells while implanting.

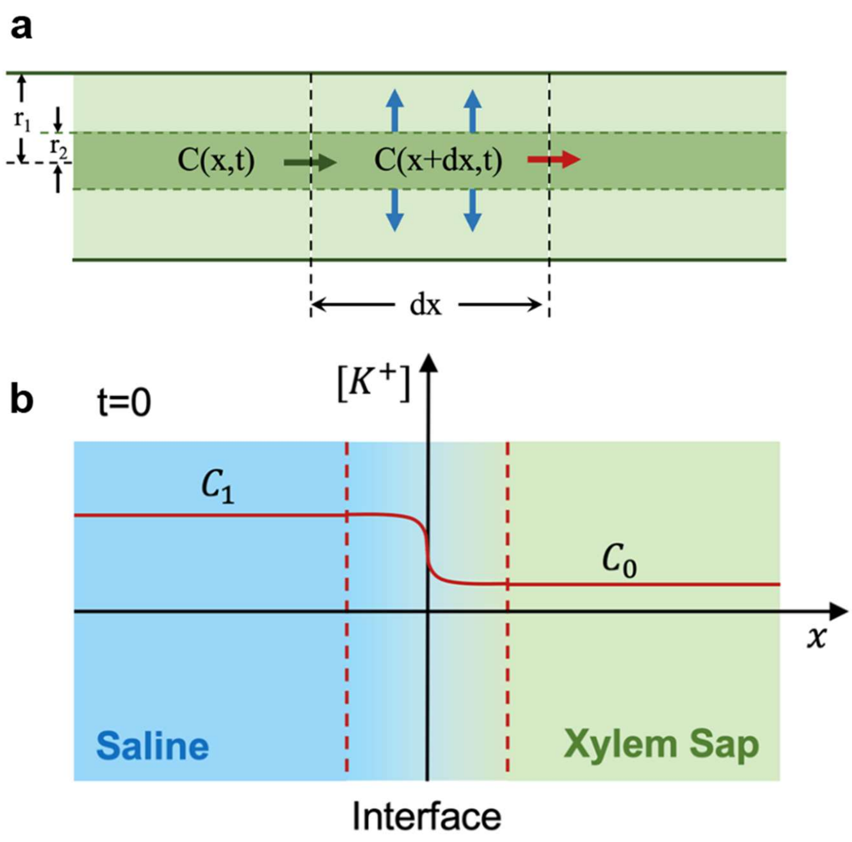

Figure S8. Modelling of xylem flow. a, 1D convection model. **b,** Boundary condition.

Supplementary discussion 2: Modelling of xylem flow

Xylem flow is described by 1D convection flow (Supplementary Figure 8a), where r_1 is the radius of the plant stem, r_2 is the effective radius of all xylem vessels (lumped into one vessel), C is the cation concentration as the function of both position x and time t. At position x+dx, the cation flow into the xylem vessel ([in]) and the cation flow out of the xylem vessel ([out]) are

$$[In] = \pi r_2^2 u C(x,t) dt$$

$$[Out] = \pi r_2^2 u C(x+dx,t) dt$$

Assuming cation concentration in xylem is quickly averaged throughout the entire stem by lateral diffusion, the concentration change at x+dx is:

$$dC = \frac{[In] - [Out]}{\pi r_1^2 dx} = \frac{\pi r_2^2 u C(x,t)dt - \pi r_2^2 u C(x+dx,t)dt}{\pi r_1^2 dx}$$

equivalently:

$$\frac{\partial C}{\partial t} = -u \frac{r_2^2}{r_1^2} \frac{\partial C}{\partial x} \tag{1}$$

At the interface between the stem and 75 mM KCl solution (x = 0):

$$C(x < 0, t = 0) = 75mM$$

$$C(x > 0, t = 0) = 2mM \ (typical\ cation\ concentration\ of\ the\ plant)$$

However, the above step function is non-analytic. Therefore, a sigmoid function was adopted to approximate the boundary condition (Figure S8b):

$$C(x,0) = \frac{C_1 - C_0}{1 + e^{\frac{x}{x_0}}} + C_0$$

where, C_0 = 2 mM, C_1 = 75 mM, and x_0 indicates the thickness of the transition region.

The analytic solution of equation (1) is:

$$C(x,t) = \frac{C_1 - C_0}{1 + e^{\frac{x-kut}{x_0}}} + C_0 \tag{2}$$

where k = $r^2{}_2$/ $r^2{}_1$, i.e., the area percentage of xylem vessels within the stem. This equation was used to fit our experimental data in Fig. 2b to determine xylem flow rate u.

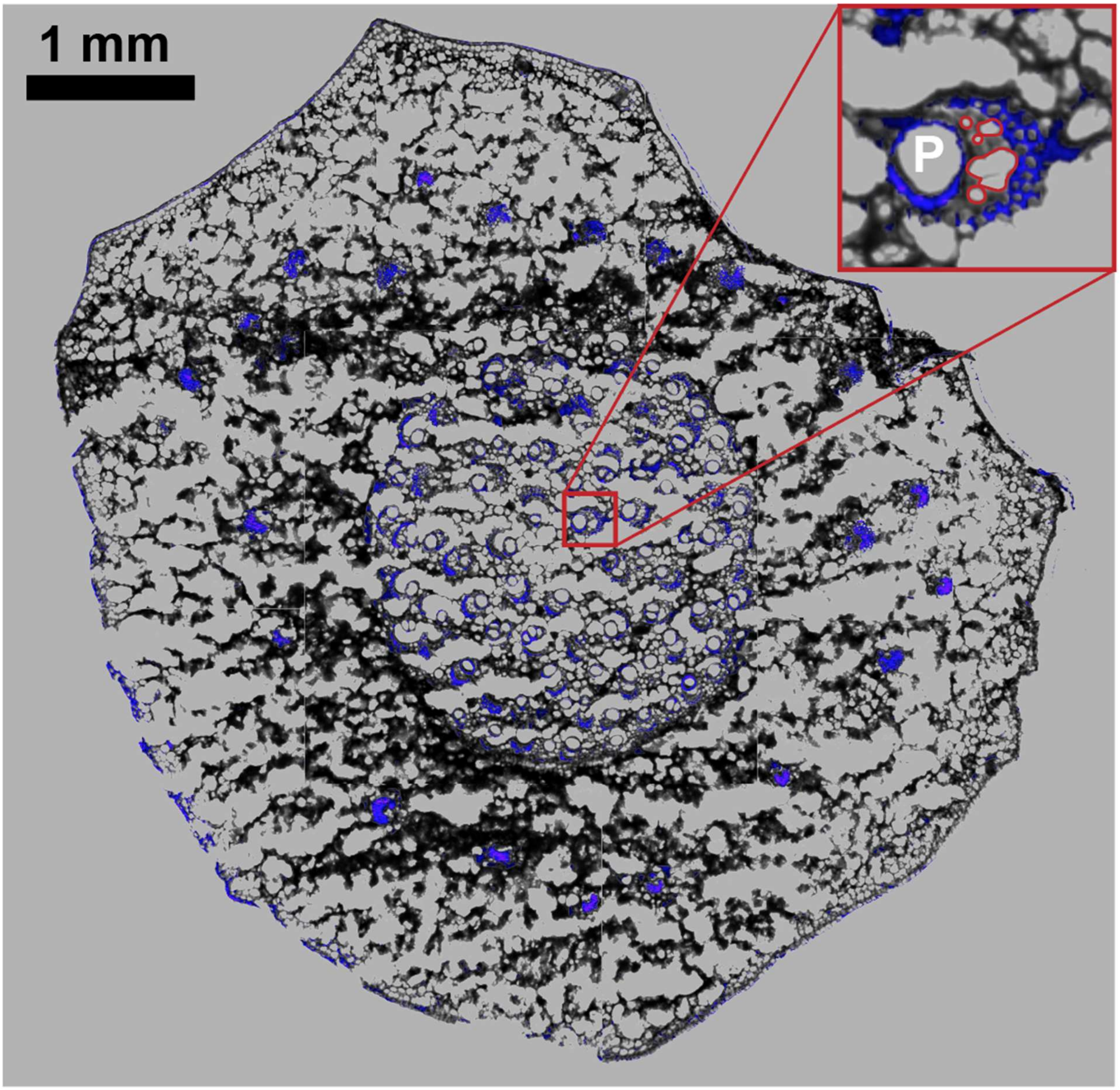

Figure S9. Superimposition of confocal fluorescence image and bright field image of plant stem cross section. Lignin is a hydrocarbon polymer enriched in vascular tissue and epidermis of plant. It exhibits fluorescence with the emission peak around 360 nm. Confocal fluorescence image shows distribution, shape and size of xylem vessels. Inset shows phloem vessel (P) and xylem vessels (red closed curves) in a vascular bundle.

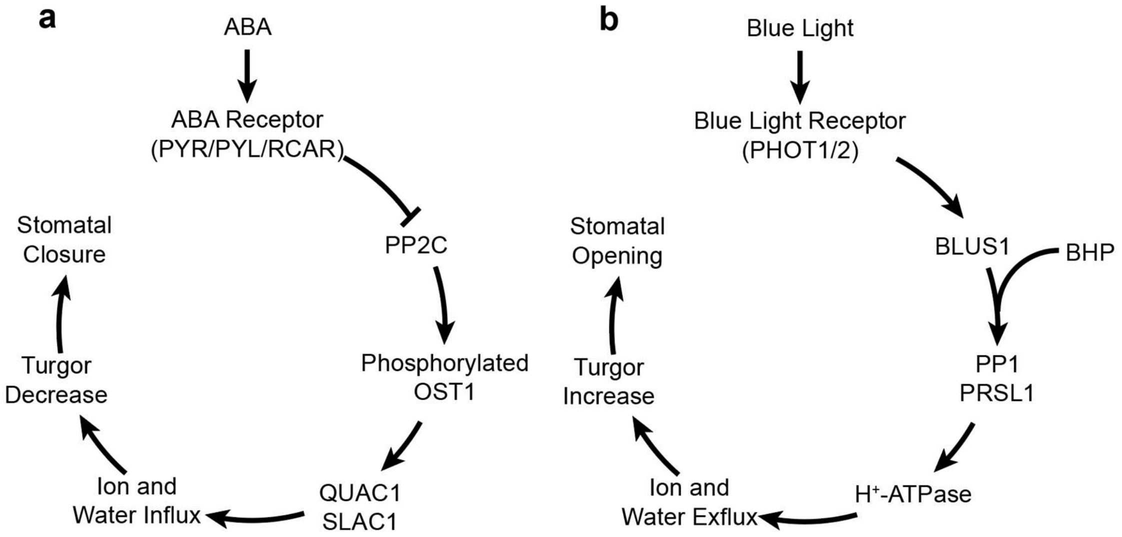

Figure S10. Signaling pathways for stomatal regulation. a, ABA induced stomatal closure. **b,** blue light induced stomatal opening.

Supplementary discussion 3: Light and ABA mediated signaling pathways for transpiration

As shown in Figure 2c, a sudden suppression of xylem flow due to stomatal closure was observed ~450s after infiltration of ABA into leaves. With additional ~640s delay, a rapid recovery of xylem flow due to stomatal re-opening was observed. The significant delay of ABA-induced stomatal closure can be explained by the underlying long signalling cascade involved (Supplementary Fig. S10a). Briefly, binding of ABA with its receptors (PYR/PYL/RCAR) inhibits type 2C protein phosphatases (PP2C). Consequently, oligosaccharyl transferase 1 (OST1) remains phosphorylated to continuously activate cation channels (QUAC1 and SLAC1). The consequent cytosolic loss of cations leads to water efflux, thereby leading to turgor decrease, and finally stomatal closure[1]. The sudden recovery of xylem flow after a long delay reveals the kinetics of the turn-off mechanisms for re-opening of stomata which may involve disabling of ABA due to hydroxylation by

ABA 8'-hydroxylase[2] or glucose conjugation[3] and degradation of OST1 by a recently discovered protein HIGH OSMOTIC STRESS GENE EXPRESSION 15 (HOS15)[4]. We envision that, combining microfiber electronics with specific agonists/antagonists and molecular/genetic approaches, the cascaded signalling pathways underlying stomatal control can be precisely deciphered.

As shown in Figure 2c, a significant delay of light-induced stomatal opening was also observed because of the long signaling cascade (Figure S10b) [5-7]. Specifically, blue light causes auto-phosphorylation of phototropins (PHOT1 and PHOT2), which leads to phosphorylation of the protein kinase BLUE LIGHT SIGNALING 1 (BLUS1) and its binding with a protein kinase - blue light-dependent H^+-ATPase phosphorylation (BHP). The complex then activates phosphatase 1 (PP1) and its regulatory subunit PRSL1, which causes phosphorylation of H^+-ATPase. Consequently, protons are transported outwards, K^+ ions and water flow in, cell turgor increases, and finally stomata open. In addition, the long delay of light-induced stomatal opening can be attributed to ABA accumulation in cytosol during the dark period before light exposure[2]. After termination of light exposure, photosynthesis stops, and consequently ABA accumulates thereby leading to stomatal closure[8]. The long delay of stomatal re-closure after turning off the light as observed in Figure 2c is likely because of the time needed for sufficient accumulation of ABA. Some researchers also pointed out the important role of red light for stomatal regulation, but the underlying mechanism is still unclear[9]. Combing with chemical, molecular, and genetic tools, our microfiber electronics shall allow scrutinization of the still not fully understood signaling pathways and their kinetics.

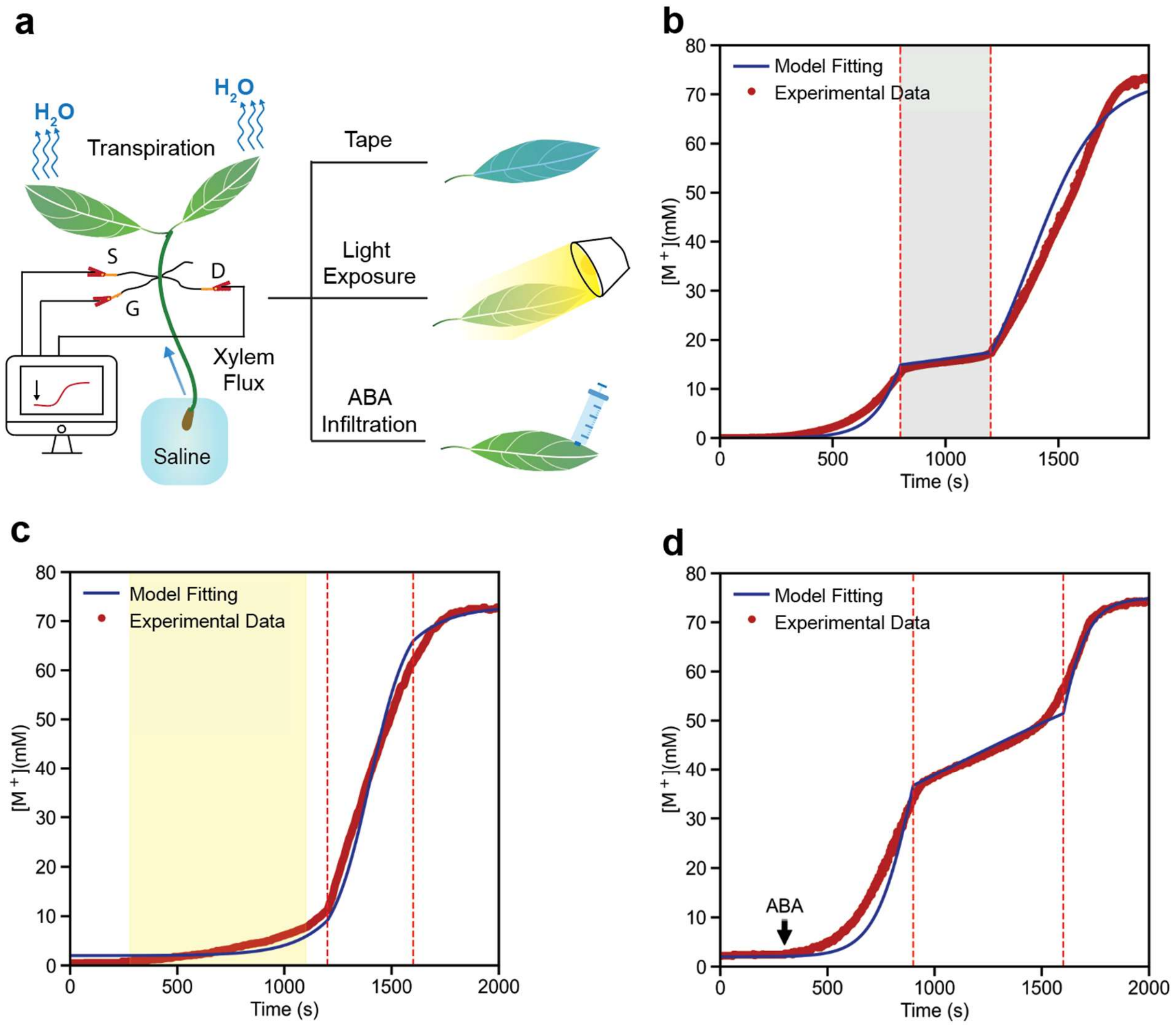

Figure S11. Fitting the experimental data in Figure 2c using our 1D convection model for xylem transport.

Supplementary discussion 4: Applying 1D convection xylem flow model to different transpiration conditions

As seen from Figure S11, our model fits well with the experimental data and can derive the xylem flow rates under various transpiration conditions (i.e., flow rate was deaccelerated by ABA hormone: from 0.094 cm/s to 0.01 cm/s; nearly halted upon total blockage of the stoma by tape: 0.092 cm/s to 0.005 cm/s; accelerated upon light exposure:

0.056 cm/s to 0.11 cm/s). This indicates generalizability of the model and reliability of the measurements. Consistently, a previous study on the same plant species (devil's ivy) also reported similar xylem flow rate, specifically, 0.08 cm/s to 0.4 cm/s, depending on plant size and cultivation conditions[10]. This further corroborates the reliability of our measurement and model. As seen from Figure S11b-c, the turning points of xylem flow rate can be clearly identified as indicated by the dashed lines. Physical blocking of stoma caused immediate change, whereas the change of xylem flow rate was much delayed in response to ABA or light exposure. For the sake of simplicity, we assumed a sudden change in flow rate at the turning points. This assumption led to small discrepancy with the experimental measurements at the turning points.

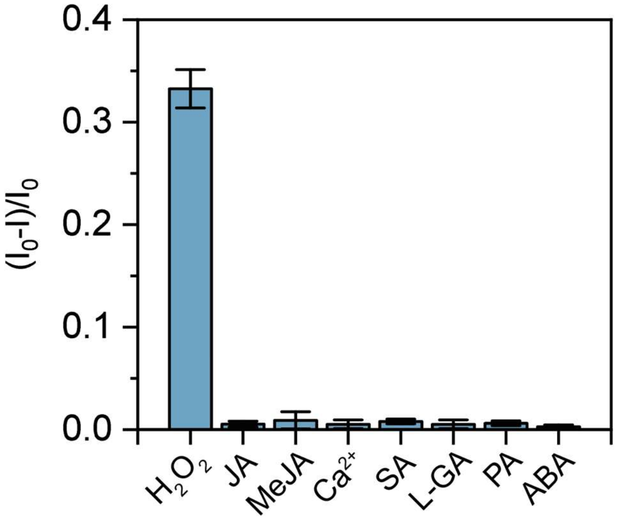

Figure S12. Selectivity of fOECT-H_2O_2 in the presence of possible interferants at their typical concentrations when the plant is stressed, e.g., by wounding (1 mM for Ca^{2+}, 100 μM for others). JA: jasmonic acid; MeJA: methyl jasmonate; SA: salicylic acid; L-GA: L-glutamic acid; PA: phenylacetic acid; ABA: abscisic acid. Data is shown as mean ± s.d. from three independent experiments.

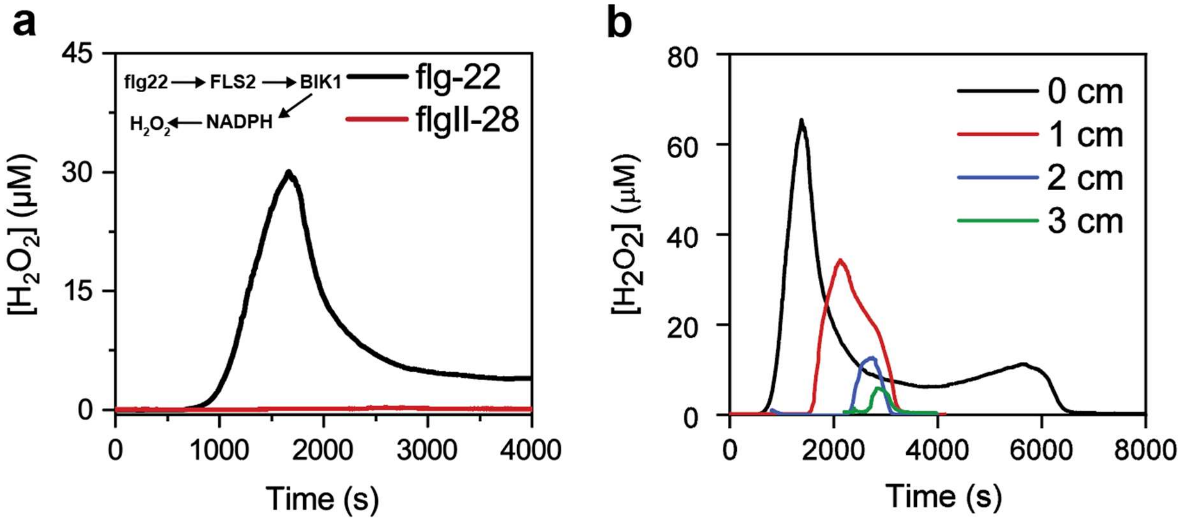

Figure S13. a, Flg22 induced H_2O_2 signal. Due to absence of the specific receptor, flgII-28, another bacterial flagellar filament, cannot induce H_2O_2 signal in plant. flgII-28 and flg-22 were injected into the plant stem 1 cm away from fOECT-H_2O_2. at the same position successively (30 min interval). Inset shows the signaling cascade for flg22 inducing generation of H_2O_2. **b,** flg22 induced H_2O_2 wave recorded at different distances (in the direction towards the leaves).

Supplementary discussion 5: Flg22 and heat induce H_2O_2 signal in plant

As compared to wounding and mild-heating triggered responses (Figure 3e), the kinetics of flg22 induced H_2O_2 wave is slower (longer delay, less steep take-off). It suggests that the underlying signaling kinetic is slow. It involves activation of the plasma membrane-localized receptor FLAGELLIN SENSITIVE2 (FLS2) and its disassociation with BOTRYTIS-INDUCED KINASE1 (BIK1)[11]. BIK1 then phosphorylates NADPH oxidase (NOX), leading to production of O^{2-} and its dismutation to form H_2O_2 by superoxide dismutase (SOD)[12].

As expected, the H_2O_2 wave decay in both amplitude and kinetics over the distance (Figure S13b). It is interesting to note that H_2O_2 concentration recorded at the wounding site rose again ~1 hour after the peak. Similar phenomenon has also been observed

previously[13]. Conceivably, upon binding with flg22, FLS2 is first endocytosed and then recycled back to cell membrane to be activated by flg22 again[14]. Our experiment reveals the kinetics of such recycling. It is also noted, compared to wounding induced wave (Figure 5a, b), the rise time (>532 s) and propagation velocity (<10 μm s^{-1}) of flg22 induced H_2O_2 wave is much slower, presumably at least in part due to the endocytosis and slow recycling of FLS2.

The mechanism of heat stress-induced H_2O_2 generation and propagation is not yet fully understood. It has been suggested that heat disrupts the organization of unsaturated fatty acid chains in membrane lipids, leading to a more fluidic and less stable membrane which, in turn, permits influx of calcium ions[15]. In addition, heat-induced conformational change of Ca^{2+} channels may also cause Ca^{2+} influx[16]. As discussed in the main text, increase of cytosol Ca^{2+} stimulates the generation of H_2O_2 wave. Unsurprisingly, the response of heat-induced H_2O_2 wave is faster than flg22-induced wave because Ca^{2+} influx is quickly caused by physical effect.

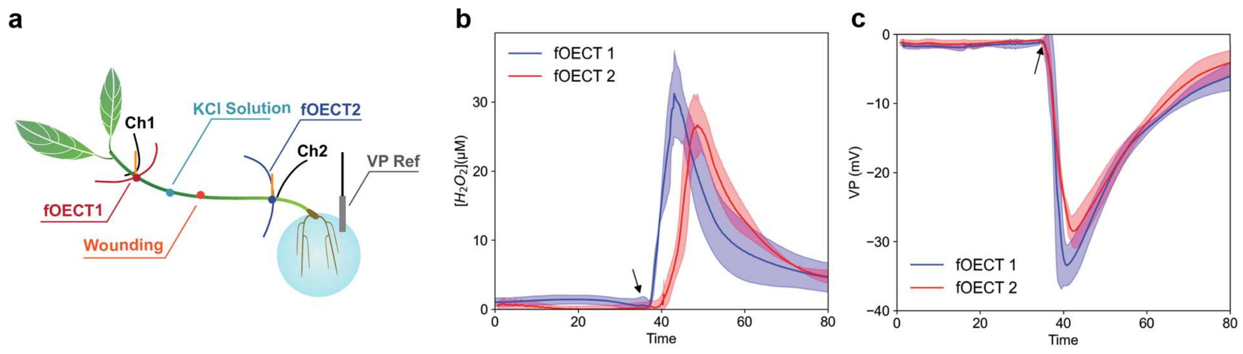

Figure S14. Infusion of KCl solution (5 mM) doesn't prevent propagation of H_2O_2 and VP waves to fOECT1. Each data point is shown as mean ± standard deviations. 3 different plants were used; both H_2O_2 and VP waves were simultaneously recorded on each plant.

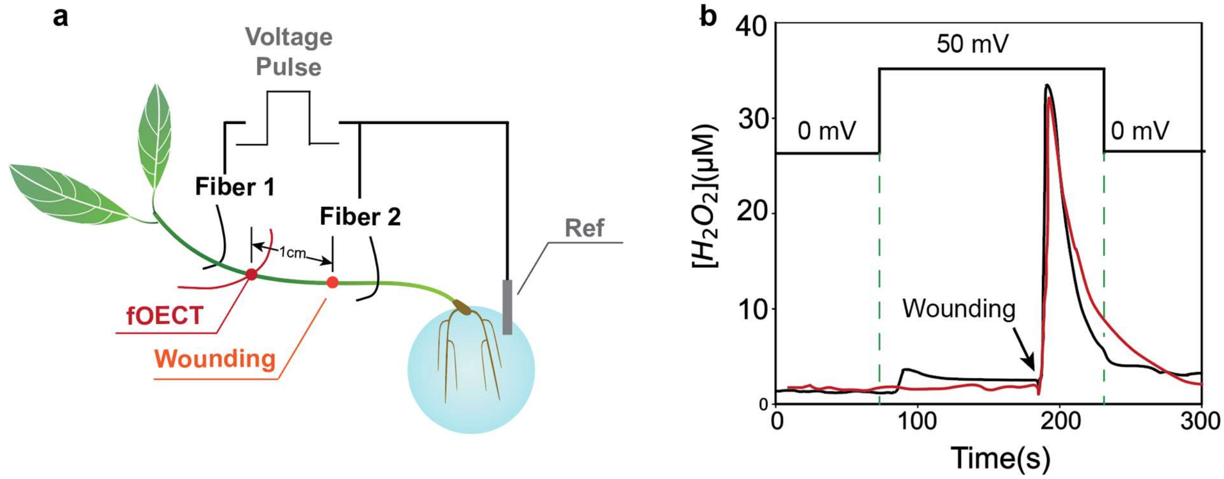

Figure S15. Voltage Influence to H_2O2-fOECT. a, Illustration of the experimental setup. A voltage pulse was applied via two conductive fibers. **b,** Wounding-induced H_2O_2 waves without (red) and with (black) application of the voltage pulse on the same plant.

Supplementary discussion 6: Voltage-induced H_2O_2 response

The possible interference of VP to H_2O_2-fOECT was investigated by applying a voltage pulse with an amplitude similar to that of VP (50 mV). The voltage pulse caused a small and lasting response of H_2O_2-fOECT (Figure S5b). The long delay (>10 s) of this response rules out the possibility of electrical interference to fOECT. Therefore, the response indicates an actual small increase of H_2O_2 (<3 μM). Such voltage-induced H_2O_2 is a new phenomenon requiring further investigation. Note that it is distinct to the rapidly-evoked large H_2O_2 wave triggered by wounding (>30 μM, <4 s delay). And as shown in Figure S5b, the presence of the voltage pulse didn't appreciably affect the amplitude or kinetics of wound-induced H_2O_2 wave. Therefore, VP, which accompanies H_2O_2 wave, doesn't electrically interfere the recording by H_2O_2-fOECT.

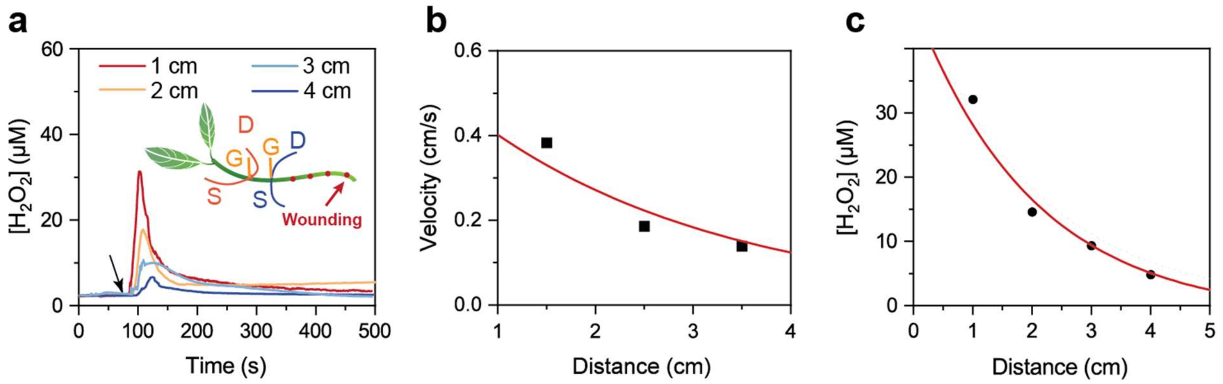

Figure S16. Distance decay of H_2O_2 wave. a, Wound was inflicted 1/2/3/4 cm away from 2 fOECTs (1 cm apart). **b,** Velocity of H_2O_2 wave decays with a constant of 2.55 cm obtained from the exponential fitting (red curve). **c**, Amplitude of H_2O_2 wave decays with a constant of 1.53 cm obtained from the exponential fitting (red curve).

Reference

1. Hsu, P.K., Dubeaux, G., Takahashi, Y., and Schroeder, J.I. (2021). Signaling mechanisms in abscisic acid-mediated stomatal closure. Plant J 105(2):307-321. https://doi.org/10.1111/tpj.15067.
2. Movahedi, M., Zoulias, N., Casson, S.A., et al. (2021). Stomatal responses to carbon dioxide and light require abscisic acid catabolism in Arabidopsis. Interface Focus 11(2):20200036. https://doi.org/10.1098/rsfs.2020.0036.
3. Munemasa, S., Hauser, F., Park, J., et al. (2015). Mechanisms of abscisic acid-mediated control of stomatal aperture. Curr Opin Plant Biol 28:154-162. https://doi.org/10.1016/j.pbi.2015.10.010.
4. Ali, A., Pardo, J.M., and Yun, D.J. (2020). Desensitization of ABA-Signaling: The Swing From Activation to Degradation. Front Plant Sci 11:379. https://doi.org/10.3389/fpls.2020.00379.
5. Yang, J., Li, C., Kong, D., et al. (2020). Light-Mediated Signaling and Metabolic Changes Coordinate Stomatal Opening and Closure. Front Plant Sci 11:601478. https://doi.org/10.3389/fpls.2020.601478.
6. Roelfsema, M.R.G., and Hedrich, R. (2005). In the light of stomatal opening: new insights into 'the Watergate'. New Phytologist 167(3):665-691.
7. Hiyama, A., Takemiya, A., Munemasa, S., et al. (2017). Blue light and CO(2) signals converge to regulate light-induced stomatal opening. Nat Commun 8(1):1284. https://doi.org/10.1038/s41467-017-01237-5.
8. Neill, S., Barros, R., Bright, J., et al. (2008). Nitric oxide, stomatal closure, and abiotic stress. J Exp Bot 59(2):165-176. https://doi.org/10.1093/jxb/erm293.
9. Matthews, J.S.A., Vialet-Chabrand, S., and Lawson, T. (2020). Role of blue and red light in stomatal dynamic behaviour. J Exp Bot 71(7):2253-2269. https://doi.org/10.1093/jxb/erz563.
10. Wistuba, N., Reich, R., Wagner, H.-J., et al. (2000). Xylem flow and its driving forces in a tropical liana: concomitant flow-sensitive NMR imaging and pressure probe measurements. Plant Biology 2(06):579-582. https://doi.org/10.1055/s-2000-16644.
11. Li, L., Li, M., Yu, L., et al. (2014). The FLS2-associated kinase BIK1 directly phosphorylates the NADPH oxidase RbohD to control plant immunity. Cell Host Microbe 15(3):329-338. https://doi.org/10.1016/j.chom.2014.02.009.
12. Kadota, Y., Sklenar, J., Derbyshire, P., et al. (2014). Direct regulation of the NADPH oxidase RBOHD by the PRR-associated kinase BIK1 during plant immunity. Mol Cell 54(1):43-55. https://doi.org/10.1016/j.molcel.2014.02.021.
13. Ngou, B.P.M., Ahn, H.K., Ding, P., and Jones, J.D.G. (2021). Mutual potentiation of plant immunity by cell-surface and intracellular receptors. Nature 592(7852):110-115. https://doi.org/10.1038/s41586-021-03315-7.
14. Robatzek, S., Chinchilla, D., and Boller, T. (2006). Ligand-induced endocytosis of the pattern recognition receptor FLS2 in Arabidopsis. Genes Dev 20(5):537-542. https://doi.org/10.1101/gad.366506.
15. Saidi, Y., Finka, A., Muriset, M., et al. (2009). The heat shock response in moss plants is regulated by specific calcium-permeable channels in the plasma membrane. The Plant Cell 21(9):2829-2843. https://doi.org/10.1105/tpc.108.065318.

16. Mittler, R., Finka, A., and Goloubinoff, P. (2012). How do plants feel the heat? Trends in biochemical sciences 37(3):118-125. https://doi.org/10.1016/j.tibs.2011.11.007.
